# MiR-200b in heme oxygenase-1-modified bone marrow mesenchymal stem cell-derived exosomes alleviates inflammatory injury of intestinal epithelial cells by targeting high mobility group box 3

**DOI:** 10.1038/s41419-020-2685-8

**Published:** 2020-06-25

**Authors:** Dong Sun, Huan Cao, Liu Yang, Ling Lin, Bin Hou, Weiping Zheng, Zhongyang Shen, Hongli Song

**Affiliations:** 1 Tianjin First Central Hospital Clinic Institute, Tianjin Medical University, 300070 Tianjin, P.R. China; 2 Department of Organ Transplantation, Tianjin First Central Hospital, 300192 Tianjin, P.R. China; 3 NHC Key Laboratory of Critical Care Medicine, 300192 Tianjin, P.R. China; 4 Tianjin Clinical Research Center for Organ Transplantation, Tianjin, P.R. China; 5 Key Laboratory of Transplant Medicine, Chinese Academy of Medical Sciences, Tianjin, P.R. China; 6 Tianjin Key Laboratory of Organ Transplantation, Tianjin, P.R. China

**Keywords:** Apoptosis, Mesenchymal stem cells

## Abstract

Heme Oxygen-1 (HO-1)-modified bone marrow mesenchymal stem cells (BMMSCs) are effective to protect and repair transplanted small bowel and intestinal epithelial cells (IECs); however, the mechanism and the role of HO-1/BMMSCs-derived exosomes is unclear. In the present study, we aimed to verify that exosomes from a HO-1/BMMSCs and IEC-6 cells (IEC-6s) co-culture system could reduce the apoptosis of IEC-6s and decrease the expression of the tight junction protein, zona occludens 1, in the inflammatory environment. Using mass spectrometry, we revealed that high mobility group box 3 (HMGB3) and phosphorylated c-Jun NH2-terminal kinase (JNK), under the influence of differentially abundant proteins identified through proteomic analysis, play critical roles in the mechanism. Further studies indicated that microRNA miR-200b, which was upregulated in exosomes derived from the co-culture of HO-1/BMMSCs and IEC-6s, exerted its role by targeting the 3′ untranslated region of *Hmgb3* in this biological process. Functional experiments confirmed that miR-200b overexpression could reduce the inflammatory injury of IEC-6s, while intracellular miR-200b knockdown could significantly block the protective effect of HO-1/BMMSCs exosomes on the inflammatory injury of IEC-6s. In addition, the level of miR-200b in cells and exosomes derived from HO-1/BMMSCs stimulated by tumor necrosis factor alpha was significantly upregulated. In a rat small bowel transplantation model of allograft rejection treated with HO-1/BMMSCs, we confirmed that the level of miR-200b in the transplanted small bowel tissue was increased significantly, while the level of HMGB3/JNK was downregulated significantly. In conclusion, we identified that exosomes derived from HO-1/BMMSCs play an important role in alleviating the inflammatory injury of IECs. The mechanism is related to miR-200b targeting the abnormally increased expression of the *Hmgb3* gene in IECs induced by inflammatory injury. The reduced level of HMGB3 then decreases the inflammatory injury.

## Introduction

Small bowel transplantation (SBTx) has become the most effective treatments for certain intestinal diseases, such as short bowel syndrome and intestinal failure^1,2^. However, because of its inherent characteristics, including high immunogenicity and susceptibility to infection, the development of SBTx has been severely limited^3,4^. Intestinal epithelial cells (IECs) and tight junction (TJ) structures are the basis for the maintenance of normal intestinal function and barrier structure^5^. The complex inflammatory environment and immune rejection in the small bowel tissue after SBTx seriously impair the activity of IECs and the integrity of TJs, which directly affect the structure and function of the transplanted small bowel. Therefore, further exploration of the occurrence and mechanism of alleviating graft IEC damage after SBTx is very important to improve the integrity of the structure and function of transplanted small bowels and ultimately prolong the survival of patients receiving SBTx. Under the poor environment resulting from a series of factors, such as post SBTx surgery and inflammatory bowel disease (IBD), the level of tumor necrosis factor alpha (TNF-α) in the intestinal micro-environment increases significantly^6–9^. TNF-α binds to TNF receptor associated factors in IECs and activates the downstream c-Jun NH2-terminal kinase (JNK) pathway, which promotes the cleavage of Caspase proteins and then induces IECs injury and apoptosis^10–12^. Targeting the JNK pathway is critical to treat IBD and other intestinal injuries^13,14^.

Mesenchymal stem cells (MSCs) are a group of pluripotent stem cells that are capable of self-renewal and multi-directional differentiation, which have anti-inflammation and immune regulation functions^15,16^. However, because of the complex inner-environment, such as ischemia and hypoxia, hypo-inflammatory infiltration, infection, and other problems, the colonization and survival rates of MSCs transplanted into the intestines are insufficient, thus limiting their clinical application^17,18^. Heme oxygenase-1 (HO-1) is a multi-functional microsomal oxidase that can increase the activity of MSCs and enhance their protective and reparative effect on damaged cells and tissues via anti-oxidative damage, anti-inflammation, inhibiting apoptosis, and facilitating cell proliferation. In our previous study, we confirmed that HO-1-modified rat bone marrow MSCs (BMMSCs) are more effective in protecting and repairing the transplanted small bowel and IECs than natural BMMSCs^6,7,19,20^. However, the mechanism by which HO-1 optimizes the activity of BMMSCs and enhances their protective effect on IECs of small bowel allografts has not been fully determined. In addition, the problems with the clinical application of MSCs remain incompletely solved by *Ho-1* gene modification, such as the heterogeneity of MSCs, the complexity of cell components, the uncertainty of their viability and differentiation, and the unpredictability of cell fate and clinical outcome after transplantation. These are still the key obstacles that limit the clinical application of MSCs. Therefore, further research on the function and mechanism of HO-1-overexpressing BMMSCs (HO-1/BMMSCs) has important basic research value and clinical significance for the ultimate clinical application of MSCs in SBTx and the transplantation other organs.

The mechanism of MSCs’ effects depends largely on their paracrine function^21^. In addition to directly secreting cytokines, MSCs also achieve their biological function by releasing exosomes (exo) outside cells. Exosomes participate in the formation of the microenvironment of cell growth, which mediates the functions of cells in the microenvironment, including immune regulation, inflammatory response, cell proliferation and differentiation, cell migration, information exchange and substance transfer between cells^21–23^. Based on the strong biological functions and broad application prospects of exosomes, some experts regard cell-free therapy as a new direction of stem cell therapy^24,25^. In the present study, we established an inflammation-injured IEC model in vitro. By extracting and purifying BMMSC-derived exosomes, we identified the protective effect of BMMSCs and HO-1/BMMSCs-derived exosomes on inflammation-injured IECs and the mechanism involved.

## Results

### Extraction, identification, and HO-1 modification of BMMSCs

**Fig. 1:**
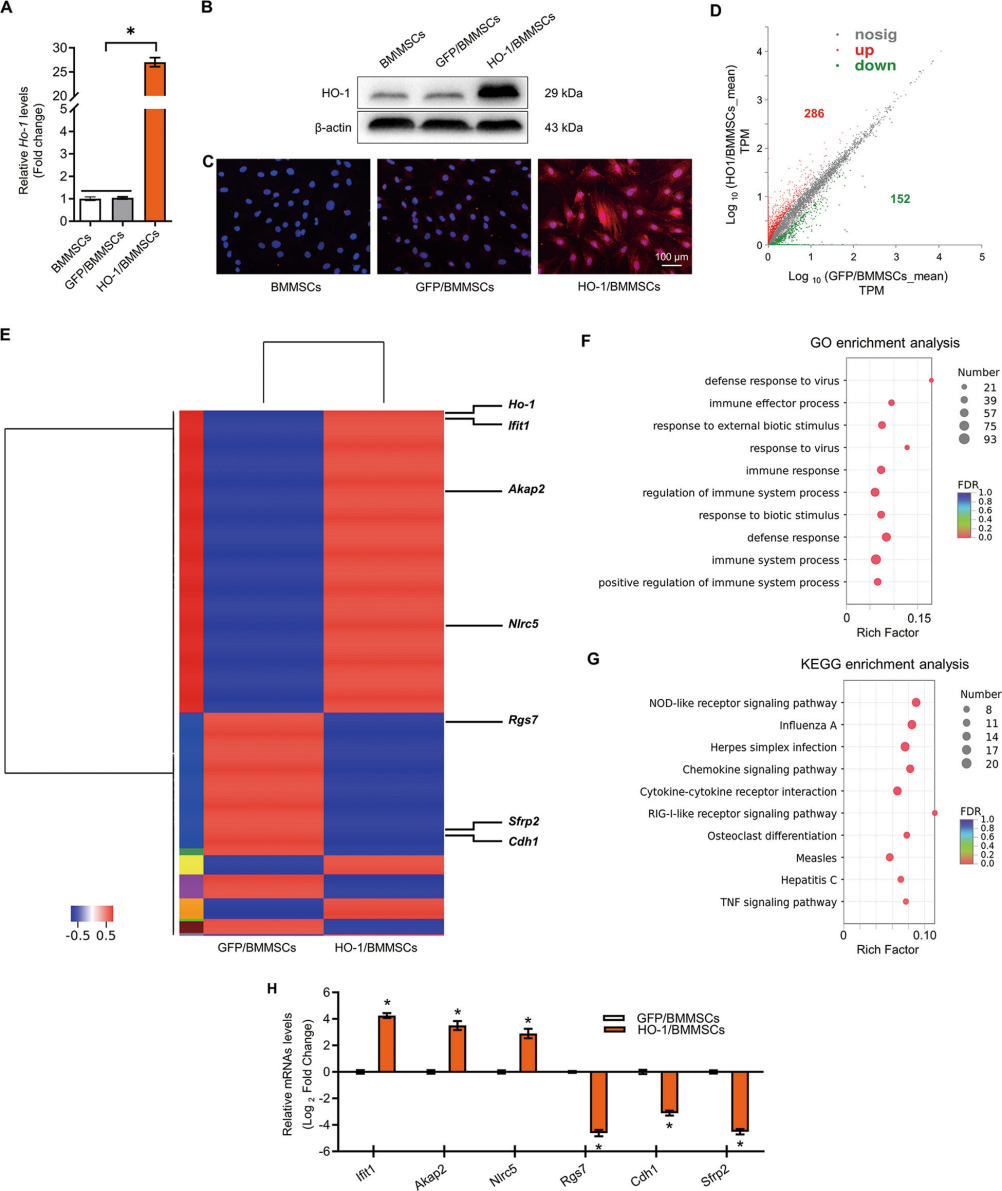
*Ho-1* gene overexpression changes the transcriptional expression profile of BMMSCs and improves immune regulation and stress tolerance abilities of BMMSCs. **a**–**c** HO-1/BMMSCs and GFP/BMMSCs were established by transfection of Adenovirus-*Ho-1* and Adenovirus-*Gfp*. The relative expression levels of the mRNA and protein of HO-1 in HO-1/BMMSCs were verified using qRT-PCR (**a**, fold change relative to BMMSCs, *n* = 4). Western blotting (**b**, *n* = 3) and immunofluorescence (**c**, *n* = 3). **d** The number of DEGs between HO-1/BMMSCs and GFP/BMMSCs. **e** A heatmap was used to represent the DEGs in HO-1/BMMSCs compared with GFP/BMMSCs. **f**, **g** Go analysis (**f**) and KEGG analysis (**g**) of the DEGs showed the biological function and signal pathways of BMMSCs regulation after *Ho-1* modification. **h** QRT-PCR validation of the mRNA levels of selected DEGs to verify the results of RNA-sequencing, including *Ifit1*, *Akap2*, *Nlrc5*, *Rgs7*, *Cdh1*, and *Snip1* (fold change relative to BMMSCs, *n* = 3). *P < 0.05. Akap2 A-kinase anchoring protein 2; BMMSCs Bone marrow mesenchymal stem cells, Cdh1 Cadherin 1; DEGs differentially expressed genes; FDR false discovery rate; GFP green fluorescent protein; GO Gene ontology; HO-1 heme oxygenase-1; Ifit1 Interferon-induced protein with tetratricopeptide repeats 1; KEGG Kyoto Encyclopedia of Genes and Genomes; Nlrc5 NLR family, CARD domain containing 5; Snip1 Smad nuclear interacting protein 1; TPM transcripts per million reads; qRT-PCR quantitative real-time reverse transcription polymerase chain reaction.

BMMSCs were isolated and cultured to the 3rd generation. Under light microscopy they showed a long fusiform morphology (Fig. S1A), expressed specific biological markers, and could be induced to differentiate into osteoblasts and adipoblasts (Fig. S1B, C). Flow cytometry (FCM) results showed the presence of integrin subunit beta 1 (CD29), Thy-1 cell surface antigen (CD90) and soluble MHC class I protein A (RT1-A) as positive markers, while the BMMSCs lacked the negative markers CD34 molecule (CD34), protein tyrosine phosphatase receptor type C (CD45), and soluble MHC class I protein B (RT1-B)^26^ (Fig. S1D). After adenovirus transfection, BMMSCs overexpressing HO-1 (HO-1/BMMSCs) and BMMSCs overexpressing green fluorescent protein (GFP/BMMSCs) were established successfully, which was verified by observation of GFP (Fig. S1E), and the expression of the HO-1 protein and mRNA, as detected by quantitative real-time reverse transcription polymerase chain reaction (qRT-PCR, Fig. 1a), western blotting (Figs. 1b and S8A), and immunofluorescence (IF, Fig. 1c).

### RNA-sequencing analysis of differentially expressed genes between HO-1/BMMSCs and GFP/BMMSCs

The results of RNA-sequencing (RNA-seq) showed that compared with GFP/BMMSCs (as negative control), there were 286 upregulated and 152 downregulated mRNAs in HO-1/BMMSCs (Fig. 1d, e, and Table S4). Gene ontology (GO) analysis showed that the differentially expressed genes (DEGs) were enriched in immune effector process, response to external biotic stimulus, regulation of immune system process, immune response, defense response, immune system process, and other biological processes (Figs. 1f and S2, Table S5). Kyoto Encyclopedia of Genes and Genomes (KEGG) analysis showed that the DEGs was enriched in NOD-like receptor signaling, chemokine signaling, cytokine-cytokine receptor interaction, RIG-I-like receptor signaling, osteoclast differentiation, and TNF-α signaling (Fig. 1g and Table S6). We examined the relative expression of six genes in HO-1/BMMSCs and GFP/BMMSCs, including Interferon-induced protein with tetratricopeptide repeats 1 (*Ifit1*, 19.18 ± 1.99-fold, *P* < 0.05) A-kinase anchoring protein 2 (*Akap2*, 11.54 ± 2.06-fold, *P* < 0.05), NLR family, CARD domain containing 5 (*Nlrc5*, 7.62 ± 1.54-fold, *P* < 0.05), Regulator of G-protein signaling 7 (*Rgs7*, 0.049 ± 0.006-fold, *P* < 0.05), Cadherin 1 (*Cdh1*, 0.100 ± 0.012-fold, *P* < 0.05), and Smad nuclear interacting protein 1 (*Snip1*, 0.044 ± 0.005-fold, *P* < 0.05), the results of which were similar to those of RNA-seq (Fig. 1h).The raw data have been submitted to the Sequence Read Archive (SRA) database (SRA accession # PRJNA600604).

### The protective effect of HO-1/BMMSCs on the inflammatory injury of IEC-6 cells

**Fig. 2:**
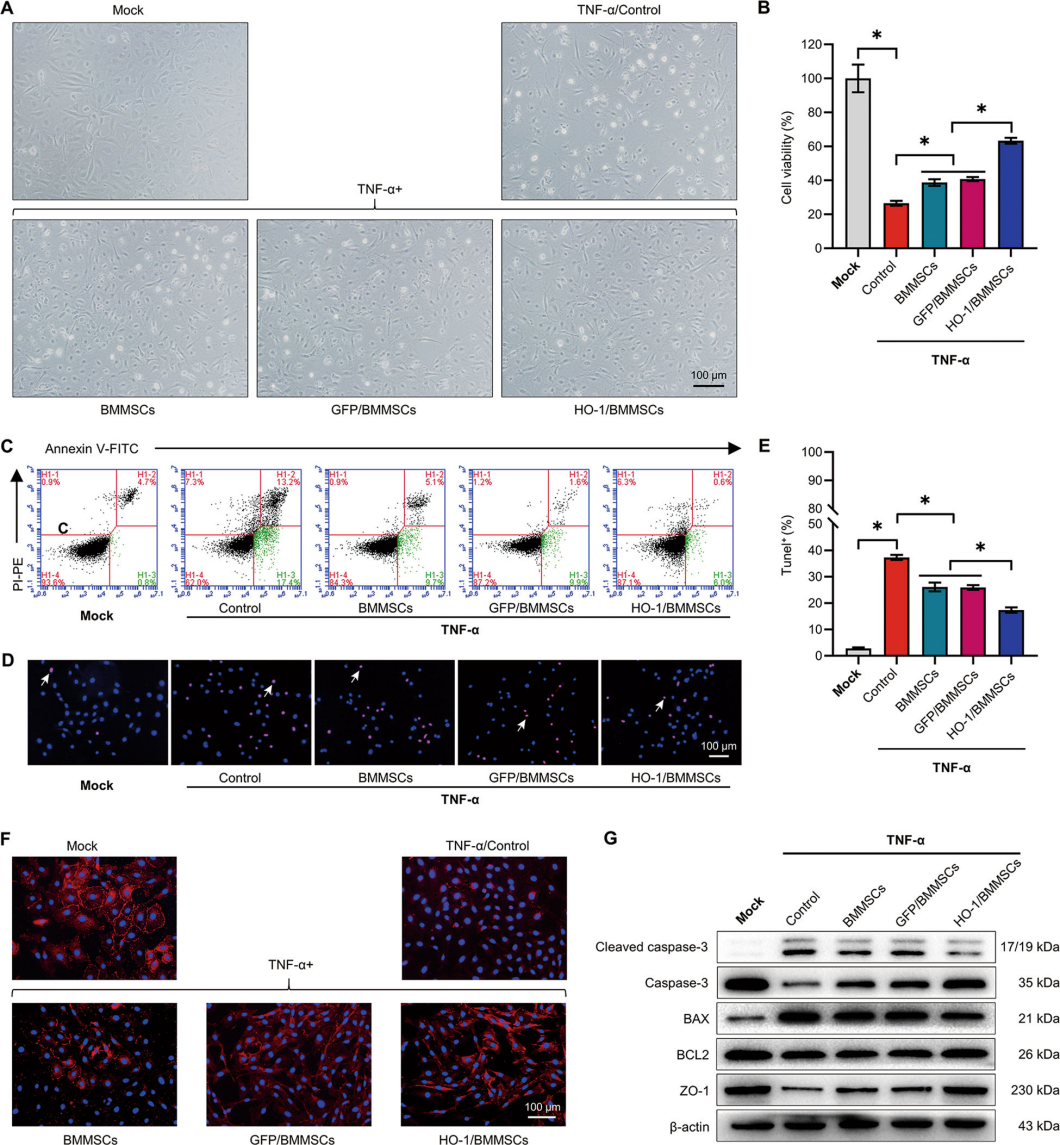
HO-1/BMMSCs have a better ability to protect IECs injured by TNF- α in vitro. **a** The morphology of the cells in each group was observed under light microscopy. **b** A Cell Counting Kit-8 was used to detect the relative viability of the cells in each group (fold change relative to the Mock group, *n* = 4). **c** Annexin V-FITC/PI-PE was used to detect the proportion of early apoptotic cells in each group (*n* = 3). **d**, **e** The proportion of TUNEL + cells (shown by white arrows) in each group (*n* = 3). **f** Labeling by immunofluorescence for the ZO-1 in each group. **g** The relative levels of (cleaved) caspase-3, BAX, BCL2 and ZO-1 protein in each group was detected using western blotting (*n* = 3). *P < 0.05. BAX B-cell lymphoma-2 associated X, apoptosis regulator; BCL2 B-cell lymphoma-2 apoptosis regulator; BMMSCs Bone marrow mesenchymal stem cells; FITC fluorescein isothiocyanate; GFP green fluorescent protein; HO-1 heme oxygenase-1; IECs intestinal epithelial cells; PI propidium iodide; PE phycoerythrin; TNF-α tumor necrosis factor alpha; TUNEL terminal deoxynucleotidyl transferase nick-end-labeling; ZO-1 Zona Occludens 1.

Models of BMMSCs, GFP/BMMSCs, and HO-1/BMMSCs protecting IEC-6 cells (IEC-6s) from inflammatory injury were established. The cells were divided into a Mock group, a TNF- α injury model group, cells co-cultured with BMMSCs group, a GFP/BMMSCs group, and a HO-1/BMMSCs group. After the addition of TNF-α and lymphocytes, the morphological destruction of IEC-6s was observed under a light microscope (Fig. 2a). The results showed that the activity of cells decreased and early apoptosis occurred in the TNF-α and lymphocytes-treated cells (Fig. 2b, c). Terminal deoxynucleotidyl transferase nick-end-labeling (TUNEL) staining showed that 34.24 ± 2.67% of the TNF-α and lymphocytes-treated cells had genomic DNA fragmentation (Fig. 2d, e). Zona occludens 1 (ZO-1) levels were reduced, as demonstrated by IF, and the TJ structures were destroyed (Fig. 2f). The viability of IEC-6s co-cultured with HO-1/BMMSCs was significantly higher than that of injury group and the BMMSCs group. Annexin V/propidium iodide (PI) staining showed that early apoptotic cells decreased to 6.42 ± 1.22% and TUNEL positive cells decreased to 19.24 ± 0.98% in the HO-1/BMMSCs group. IF showed that TJ structures were markedly improved. Western blotting showed that the proportion of cleaved caspase-3 and the B-cell lymphoma-2 associated X, apoptosis regulator (BAX)/B-cell lymphoma-2 apoptosis regulator (BCL2) ratio in IEC-6s co-cultured with HO-1/BMMSCs were significantly lower than those in the injury group and the BMMSCs co-culture group; and the level of ZO-1 increased significantly (Figs. 2g and S8B). These results demonstrated that HO-1/BMMSCs are more effective than BMMSCs in protecting IEC-6s from inflammatory injury.

### Protective effect of exosomes derived from BMMSCs modified by HO-1 on inflammatory injury of IEC-6s

**Fig. 3:**
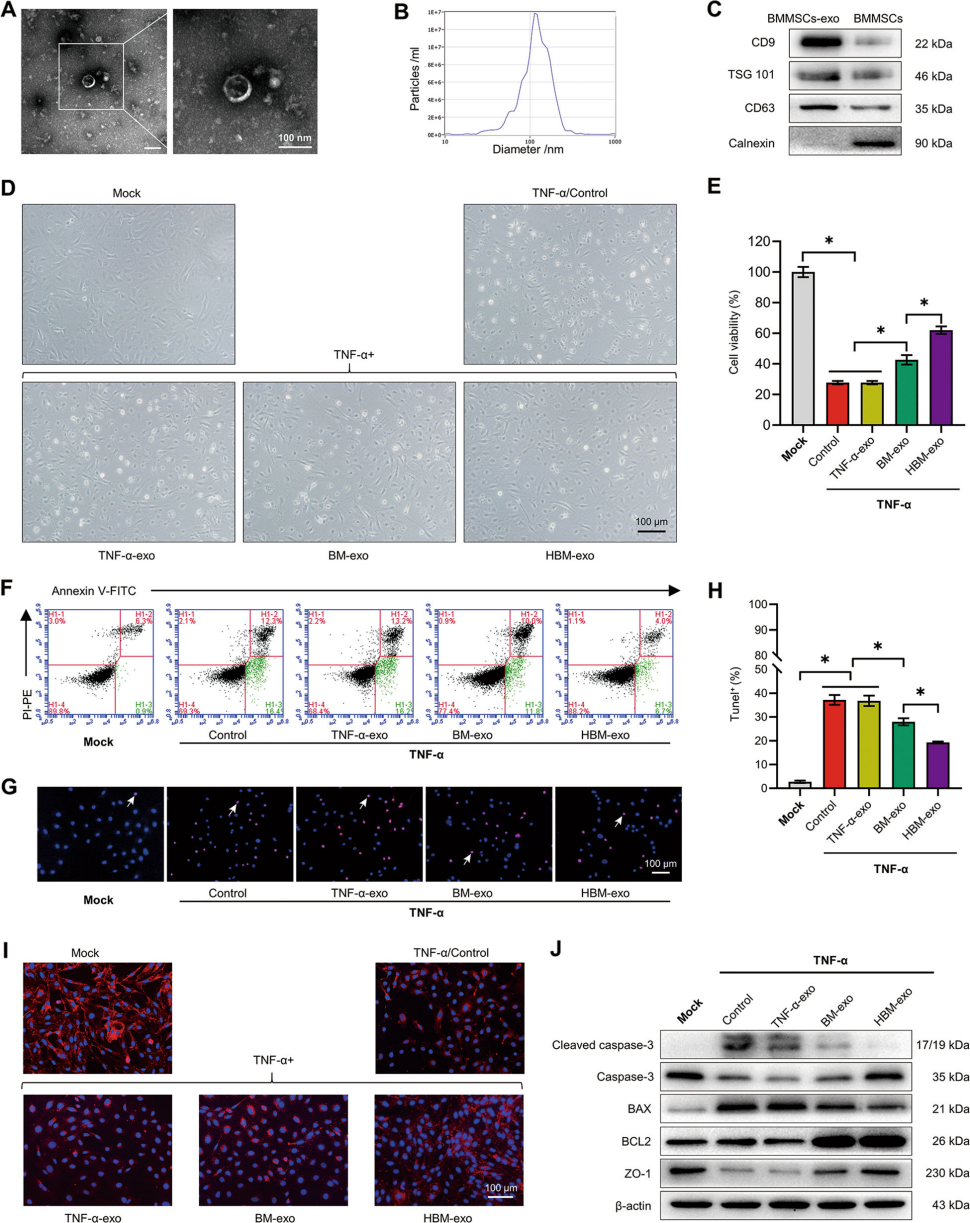
Exosomes in a co-culture system of IECs treated with HO-1/BMMSCs (HBM-exo) have a better protective effect than BM-exo on IEC-6s injured by inflammation. **a**–**c** The extracted exosomes were identified. After the exosomes were extracted from BMMSCs, they were observed to be vesicular under transmission electron microscope (**a**) and were detected using Nanoparticle tracking analysis (**b**, dilution factor: 600). Western blotting confirmed the positive expression of CD63, TSG101, and CD9 as exosome marker proteins and the negative expression of Calnexin (**c**). **d** The morphology of the IECs in each group was observed under a light microscope. **e** The relative cell viability of each group was detected (fold change relative to the Mock group, *n* = 5). **f** Annexin V-FITC/PI-PE was used to detect the proportion of early apoptotic cells in each group (*n* = 4). **g**, **h** The proportion of TUNEL + cells (shown by the white arrows) in each group (*n* = 3). **i** Labeling by immunofluorescence for the ZO-1 protein in each group. **j** The relative levels of (cleaved) caspase-3, BAX, BCL2, and ZO-1 in each group was detected using western blotting (*n* = 3). *P < 0.05. BAX B-cell lymphoma-2 associated X, apoptosis regulator; BCL2, B-cell lymphoma-2 apoptosis regulator; BMMSCs Bone marrow mesenchymal stem cells; BM-exo BMMSCs co-culture system exosomes; CD63 lysosomal membrane-associated glycoprotein 3; CD9 CD9 molecule; Exo exosomes; FITC fluorescein isothiocyanate; HO-1 heme oxygenase-1; HBM-exo HO-1/BMMSCs co-culture system exosomes; IECs intestinal epithelial cells; PE phycoerythrin; PI propidium iodide; TNF-α tumor necrosis factor alpha; TNF-α-exo tumor necrosis factor alpha-treated IEC-6s system exosomes; TSG 101 tumor susceptibility 101; TUNEL terminal deoxynucleotidyl transferase nick-end-labeling; ZO-1 Zona Occludens 1.

Under the electron microscope, the diameter of the extracted bubbly exosomes was ~120 nm (Fig. 3a). The nanoparticle tracking analysis (NTA) results showed that exosomes formed aggregates of 125.3 ± 57.0 nm, and original concentration was 9.9 × 10^10^ particles/mL (Fig. 3b). Western blotting showed that the exosomes were positive for lysosomal membrane-associated glycoprotein 3 (CD63), tumor susceptibility gene 101 (TSG 101) and CD9 molecule (CD9), and negative for Calnexin, which met the criteria for identification as exosomes (Fig. 3c). We isolated exosomes from the substrate of co-culture system in the TNF-α inflammatory injury group (TNF-α-exo), BMMSCs-exo (BM-exo) in the BMMSCs co-culture group, and HO-1/BMMSCs-exosomes (HBM-exo) in the HO-1/BMMSCs co-culture group. Then, the exosomes were used to treat IEC-6s for 48 h after damage. The experiment was divided into a Mock group, a TNF-α group, and TNF-α plus TNF-α-exo, BM-exo, and HBM-exo groups. The results showed that compared with the BM-exo group, HBM-exo treatment significantly improved cell status (Fig. 3d), increased the cell viability of IEC-6s (Fig. 3e), resulted in a lower proportion of early apoptosis cells (Fig. 3f), increased the level of ZO-1, and protected the TJ structures (Fig. 3i). The proportion of TUNEL positive cells was lower in the HBM-exo group (Fig. 3g, h). The proportion of cleaved caspase-3 and the BAX/BCL2 ratio was lower than that of the BMMSCs co-culture group, and the level of ZO-1 increased significantly (Fig. 3j and S8C). These results demonstrated that HBM-exo are more effective than BM-exo in protecting IEC-6s from inflammatory injury.

### Proteomic analysis of IEC-6s in different groups

**Fig. 4:**
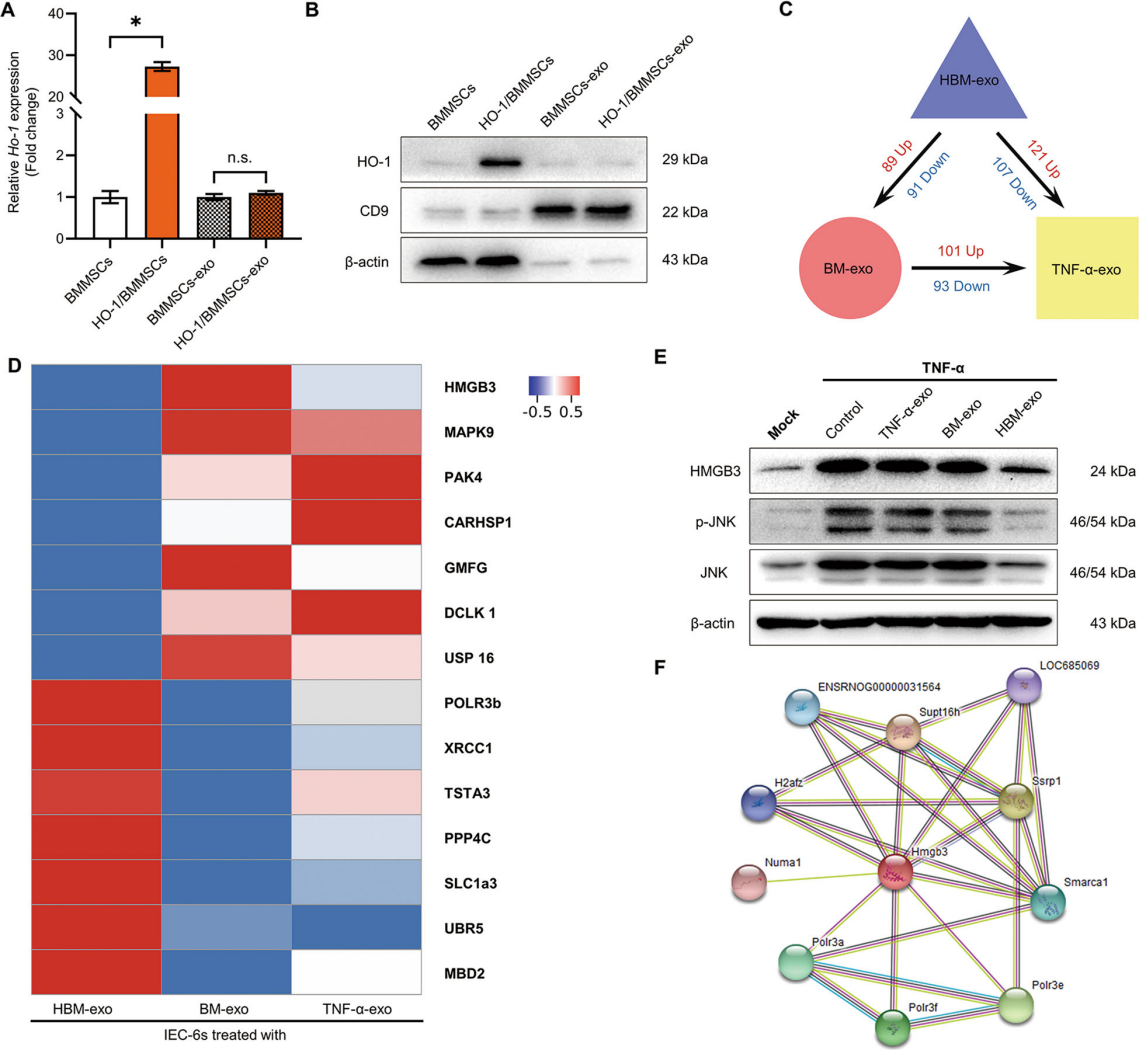
HBM-exo downregulated the intracellular HMGB3/JNK pathway. **a**, **b** QRT-PCR and western blotting were used to detect the expression levels of *Ho-1* mRNA (**a**, fold change relative to cells or exosomes of BMMSCs, *n* = 3) and HO-1 protein (**b**) of HO-1 in BMMSCs and HO-1/BMMSCs cells and exosomes. **c**, **d** Proteomic analysis of the effect of HBM-exo on inflammatory stimulation. The HBM-exo treatment group, BM-exo treatment group, and TNF-α-exo treatment group were compared; the number of DAPs (**c**) and the DAPs (**d**) among the three groups is shown using a Heatmap. **e** The intracellular HMGB3, phosphorylated (p-) JNK, and JNK protein of the Mock group, TNF-α group, and TNF-α-exo, BM-exo, and HBM-exo groups was detected using western blotting. **f** The proteins interacting with HMGB3 were searched in the String database. *P < 0.05; ns no statistic, *P* > 0.05. BMMSCs bone marrow mesenchymal stem cells; BM-exo BMMSCs co-culture system exosomes; CD9 CD9 molecule; DAPs differentially abundant proteins; Exo exosomes; HMGB3, high mobility group box 3; HO-1 heme oxygenase-1; HBM-exo HO-1/BMMSCs co-culture system exosomes; IECs intestinal epithelial cells; JNK C-Jun NH2-terminal kinase; qRT-PCR quantitative real-time reverse transcription polymerase chain reaction; TNF-α tumor necrosis factor alpha; TNF-α-exo tumor necrosis factor alpha-treated IEC-6s system exosomes.

We detected the HO-1 mRNA and protein expression levels in HO-1/BMMSCs and BMMSCs cells and exosomes. The results showed that there was no significant difference in mRNA and protein levels of HO-1 in exosomes (Fig. 4a, b). The proteins in the inflammation-injured IEC-6s treated with TNF-α-exo, BM-exo, and HBM-exo were analyzed using mass spectrometry. The results showed that compared with the TNF-α-exo group, 101 proteins were upregulated and 93 were downregulated in the inflammation-injured IEC-6s treated with BM-exo (Figs. 4c, d and S3, Table S7–9). Compared with the BM-exo group, 89 proteins were upregulated and 91 proteins were downregulated in IEC-6s treated with HBM-exo (Figs. 4c, d and S4, Table S10–12). When compared with the inflammatory injured IEC-6s treated with TNF-α-exo, the expression of 121 proteins in the HBM-exo group were upregulated and 107 were downregulated (Figs. 4c, d and S5, Table S13–15). The mass spectrometry proteomics data have been deposited at the ProteomeXchange Consortium via the PRIDE partner repository with the dataset identifier PXD017002.

Based on the comprehensive analysis of the proteomic results, we focused on the expression levels of the High mobility group box 3 (HMGB3) protein, and the level of phosphorylated (p-) JNK. We found that the relative levels of HMGB3 and p-JNK protein in IEC-6s treated with HBM-exo were significantly lower than those in BM-exo treated group (Fig. 4e and S8D). The proteins predicted or confirmed to interact with HMGB3 (Figs. 4f and Table S16) were analyzed in the String database (https://string-db.org/)^27^. The results of proteomic analysis hinted that the HMGB3 and JNK pathway might play a role in the protection exerted by HBM-exo against inflammatory injury in IEC-6s.

### HMGB3 promotes the damage of IEC cells

**Fig. 5:**
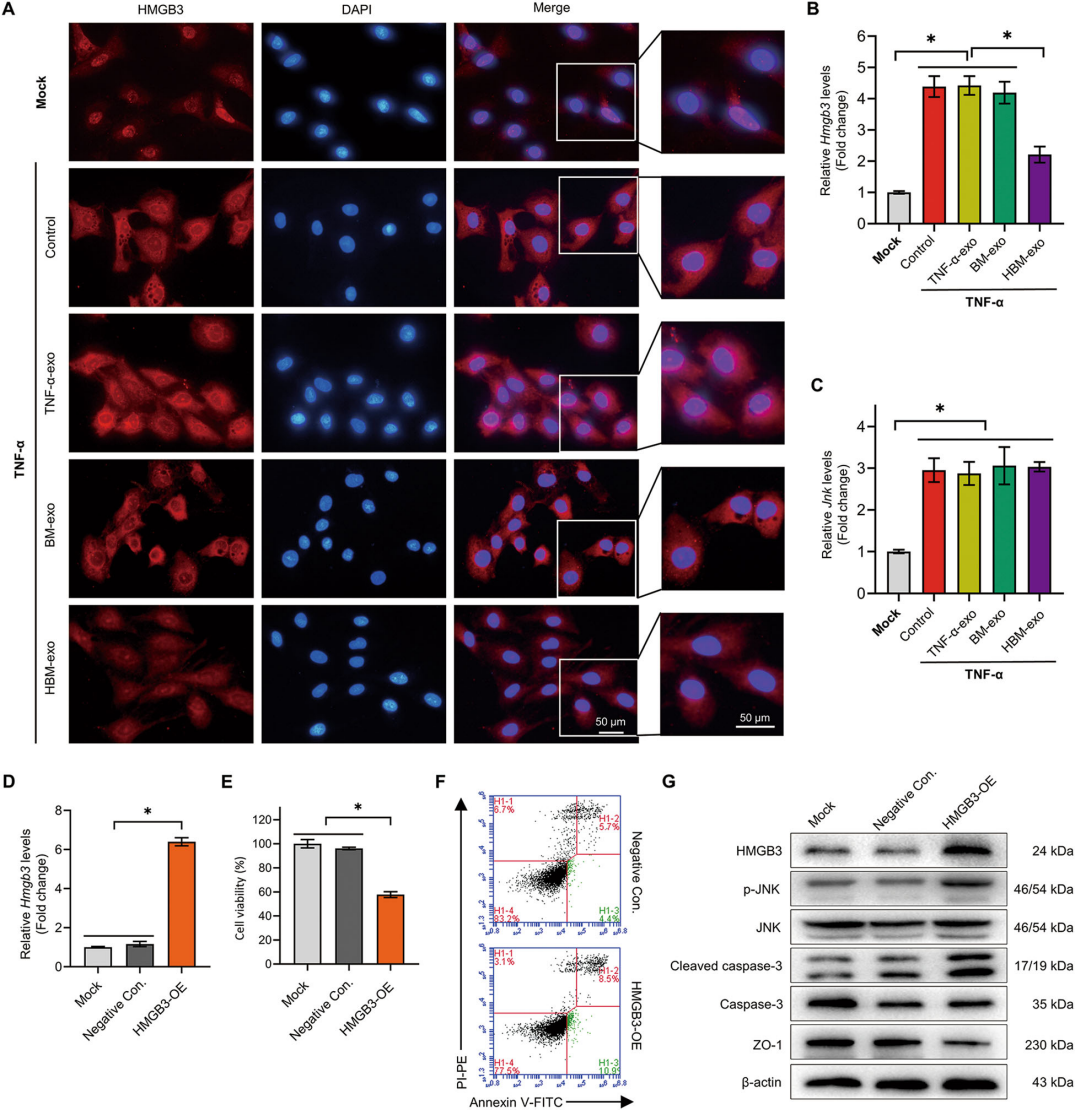
HBM-exo alleviated the injury of IECs cells by downregulating the abnormal HMGB3/JNK pathway induced by inflammatory injury. **a** HMGB3 protein of IECs in the Mock group, TNF-α group, TNF-α-exo, BM-exo, and HBM-exo groups was labeled using immunofluorescence. **b**, **c** QRT-PCR was used to detect the expression level of *Hmgb3* and *Jnk* in each group (fold change to relative the Mock group, *n* = 4). **d** The negative control (Negative Con.) and HMGB3 overexpression (HMGB3-OE) IEC-6s were established by transfection of the negative control and the *Hmgb3* overexpression plasmid; **e** The Cell Counting Kit-8 method was used to detect the relative cell viability (fold change relative to the Mock group, *n* = 4); **f** Annexin V-FITC/ PI-PE double staining was used to mark the proportion of early apoptosis (*n* = 4). **g** The protein expression levels of phosphorylated (p-) JNK, (cleaved) caspase-3, and ZO-1 proteins affected by HMGB3 were detected using western blotting (*n* = 4).*P < 0.05; BMMSCs Bone marrow mesenchymal stem cells; BM-exo BMMSCs co-culture system exosomes; DAPI 4,6-diamino-2-phenyl indole; Exo exosomes; FITC fluorescein isothiocyanate; HMGB3 high mobility group box 3; HO-1 heme oxygenase-1; HBM-exo HO-1/BMMSCs co-culture system exosomes; IECs, intestinal epithelial cells; JNK, C-Jun NH2-terminal kinase; PE phycoerythrin; PI propidium iodide; qRT-PCR quantitative real-time reverse transcription polymerase chain reaction; TNF-α tumor necrosis factor alpha; TNF-α-exo tumor necrosis factor alpha-treated IEC-6s system exosomes; ZO-1 Zona Occludens 1.

Next, we carried out further research on the HMGB3/JNK pathway. IF results showed that HMGB3 was mainly distributed in the nuclei of normal IEC-6s. After TNF-α treatment, the level of HMGB3 in the cytoplasm increased significantly, while the cytoplasmic level of HMGB3 decreased significantly after HBM-exo treatment (Fig. 5a). The relative expression level of the *Hmgb3* mRNA decreased significantly after treatment with HBM-exo (Fig. 5b). The results suggested that abnormally high levels of HMGB3 in the cytoplasm might cause damage to IECs.

To clarify the effect of HMGB3 on IEC-6s, we overexpressed *Hmgb3* mRNA in IEC-6s via plasmid transfection (Fig. 5d). The activity of IEC-6s decreased and the number of apoptotic cells increased significantly. Western blotting showed that after *Hmgb3* overexpression, the level of p-JNK and cleaved caspase-3 both increased significantly, and the level of ZO-1 decreased significantly (Figs. 5e–g and S9A).

### MiR-200b directly targets the 3′ UTR region of *Hmgb3* and regulate the expression of HMGB3

**Fig. 6:**
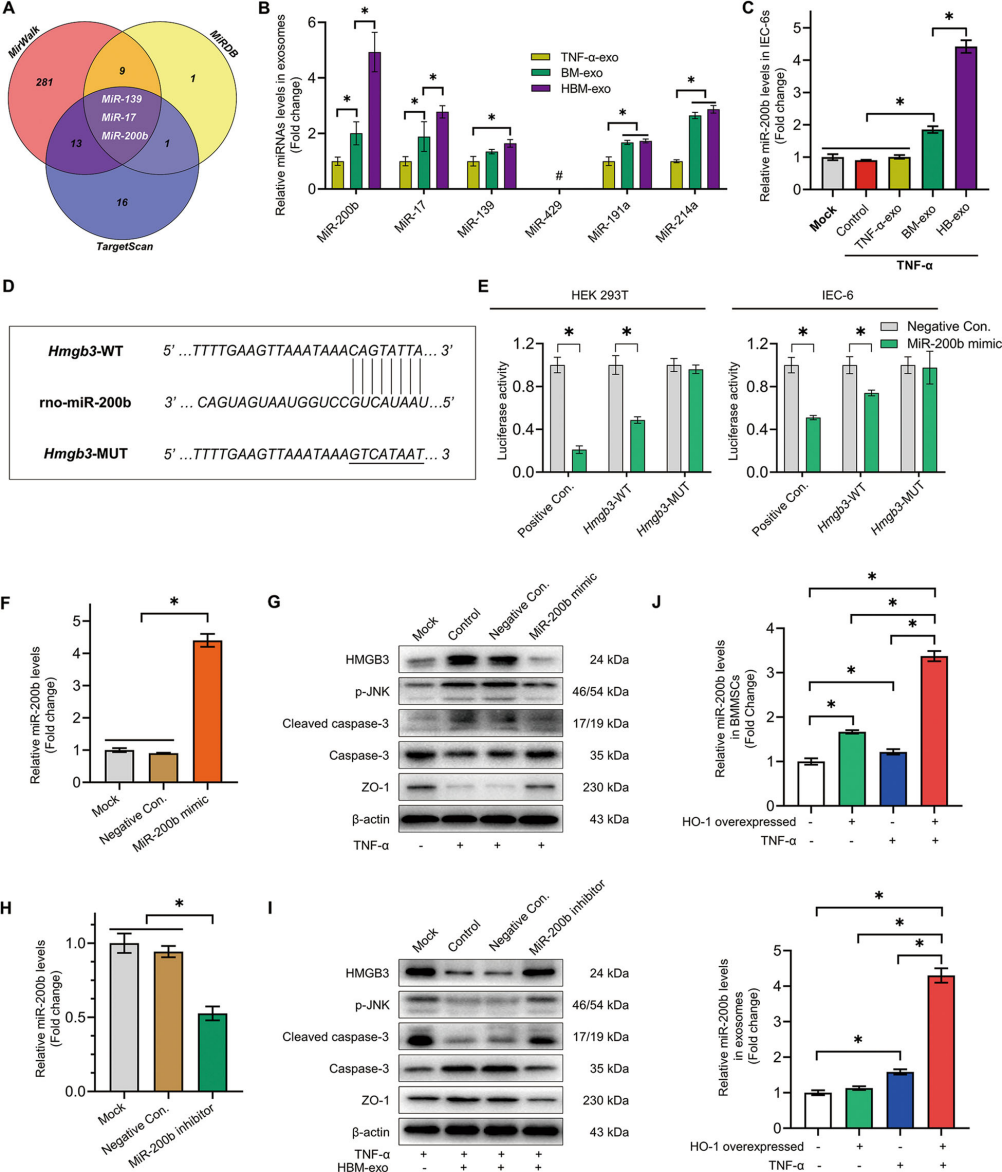
MiR-200b in HBM-exo targets *Hmgb3* to attenuate the inflammatory injury of IECs. **a** TargetScan, miRWalk, and miRDB database predicted the microRNAs targeting the 3′ UTR of *Hmgb3*. **b** QRT-PCR was used to detect the relative expression levels of miR-200b, miR-17, miR-139, miR-429, miR-191a, and miR-214a in TNF-α-exo, BM-exo, and HBM-exo groups (normalized by U6, fold change to TNF-α-exo, *n* = 5). **c** QRT-PCR was used to detect the relative expression of miR-200b in the Mock, TNF-α, TNF-α-exo, BM-exo, and HBM-exo groups (fold change relative to the Mock group, *n* = 4). **d**, **e** The plasmids HMGB3-WT (wild-type), HMGB3-MUT (mutant-type), positive control (Positive Con.) and negative control (Negative Con.) were constructed. Dual luciferase reporter assays were used to verify the targeted binding relationship between miR-200b and the 3′ UTR region of *Hmgb3* in HEK293T cells and IEC-6s (*n* = 3). **f**, **g** The protective effect of overexpression of miR-200b on inflammatory injury of IEC-6s. Transfection of miR-200b mimic and Negative Con., western blotting to verify the Mock group, Control group under TNF-α damage, and the Negative Con group. The relative expression levels of HMGB3, phosphorylated (p-) JNK, and other proteins in IEC-6s of the control and miR-200b mimic groups (*n* = 3); **h**, **i** Interference with miR-200b expression to block the protective effect of HBM-exo on inflammatory IEC-6s. Transfection of an miR-200b inhibitor and Negative Con. After treatment, the relative expression levels of HMGB3 and p-JNK were detected in the model of IEC-6s protected by HBM-exo (*n* = 3). **j** The relative expression levels of miR-200b in BMMSCs, HO-1/BMMSCs, TNF-α + BMMSCs, TNF-α + HO-1/BMMSCs cells, and exocrine bodies were detected (fold change to BMMSCs, *n* = 5). *P < 0.05, # undetected. BMMSCs bone marrow mesenchymal stem cells; BM-exo BMMSCs co-culture system exosomes; Exo exosomes; HMGB3 high mobility group box 3; HO-1 heme oxygenase-1; HBM-exo HO-1/BMMSCs co-culture system exosomes; IECs intestinal epithelial cells; JNK C-Jun NH2-terminal kinase; qRT-PCR quantitative real-time reverse transcription polymerase chain reaction; TNF-α tumor necrosis factor alpha; UTR untranslated region.

We predicted that the microRNAs miR-200b, miR-17, miR-139, miR-429, miR-191a, and miR-214a might target *Hmgb*3, and detected the expression of exosomes in the substrate of the co-culture system (Fig. 6a and Table S17). Compared with BM-exo, miR-200b (4.93 ± 0.71-fold vs. 2.01 ± 0.41-fold, fold change relative to TNF-α-exo) in HBM-exo were expressed at significantly higher levels (Fig. 6b). The results showed that the content of miR-200b in inflammation-injured IEC-6s increased significantly after HBM-exo treatment (Fig. 6c). Dual luciferase reporter assays using IEC-6s and HEK293T cells showed that miR-200b could directly bind to the 3′ untranslated region (UTR) of *Hmgb3*-wild-type (WT), but not to the 3′ UTR of *Hmgb3*-mutant-type (MUT), indicating that HMGB3 expression was regulated by miR-200b (Fig. 6d, e, Table S18).

### MiR-200b in HBM-exo can protect IEC-6s from inflammatory injury

To verify the function of miR-200b, we first transfected the miR-200b-mimic, to establish IEC-6s overexpressing miR-200b (Fig. 6f), and then carried out inflammatory injury with TNF-α and lymphocytes. The results showed that miR-200b significantly reversed the upregulation of the HMGB3/JNK pathway, decreased the expression of cleaved caspase-3, and protected the level of ZO-1 (Fig. 6g and S9B). Then, after transfection of the miR-200b inhibitor into IEC-6 cells treated with HBM-exo, the results showed that the miR-200b inhibitor significantly blocked the protective effect of HBM-exo on inflammation injured IEC-6s (Figs. 6h, i and S9C).

To clarify the source of the increased miR-200b expression in the exosomes, we examined the relative expression of miR-200b in four kinds of cells and exosomes: BMMSCs, HO-1/BMMSCs, TNF-α (50 ng/mL)-treated BMMSCs, and TNF-α-treated HO-1/BMMSCs. The results showed significantly increased expression of miR-200b in exosomes secreted by BMMSCs stimulated by TNF-α. The relative expression of miR-200b in HO-1/BMMSCs stimulated by TNF-α was significantly higher than that of HO-1/BMMSCs and BMMSCs stimulated by TNF-α (Fig. 6j).

### The expression of miR-200b was significantly increased in the rat model of immune rejection after HO-1/BMMSCs repair of SBTx

**Fig. 7:**
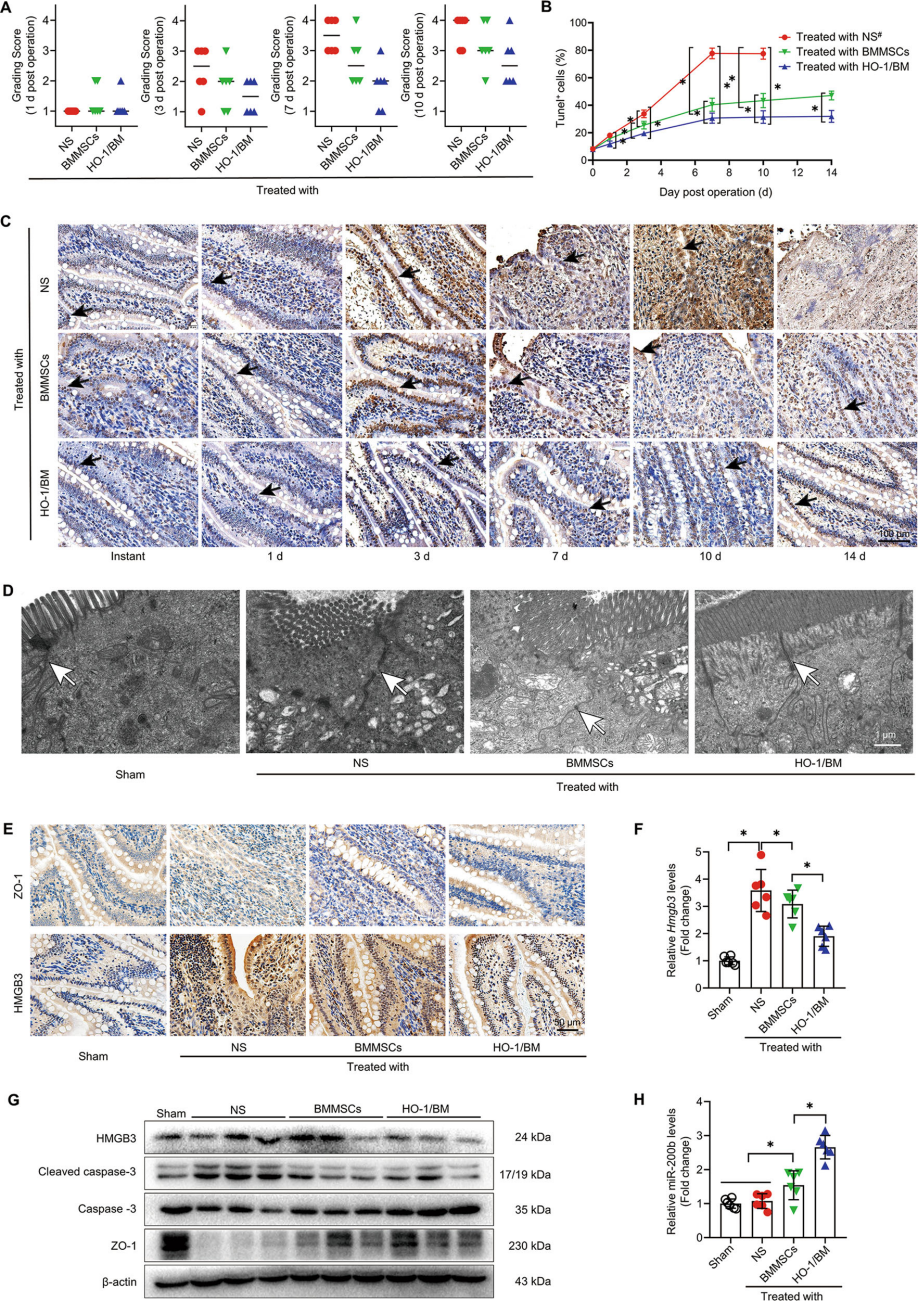
HO-1/BMMSCs can upregulate the expression of miR-200b in the intestinal tissue of a rat allogeneic SBTx model and alleviate intestinal injury. **a** The immune rejection score of intestinal histopathology in NS-treated, BMMSCs-treated, and HO-1/BMMSCs-treated SBTx rats (1, 3, 7, and 10 day post surgery, *n* = 6, median). **b**, **c** The proportion of TUNEL (+) cells (shown by black arrows) in intestinal tissue of SBTx rats in each group (Instant, and 1, 3, 7, 10, and 14 day post surgery). **d** Transmission electron microscopy was used to observe the ultrastructure of intestinal tissue in the above-mentioned groups and Sham group after small bowel transplantation (3 day post surgery). The tight junctions are shown using white arrows. **e** ZO-1 and HMGB3 proteins in intestinal tissue of rats after SBTx were labeled using immunohistochemistry (3 days after surgery). **f**, **g** QRT-PCR was used to detect the relative expression levels of *Hmgb3* mRNA in intestinal tissues of rats after small bowel transplantation (fold change relative to the Sham group, **f**), and the relative expression levels of HMGB3, (cleaved) caspase-3, and ZO-1 were detected using western blotting (3 day post operation, **g**); **h** QRT-PCR was used to detect the relative expression level of miR-200b in intestinal tissue of SBTx rats (fold change relative to the Sham group, 3 day post surgery). *P < 0.05. #: the data of NS treatment group could not be analyzed at 14 day post surgery. BMMSCs (BM) Bone marrow mesenchymal stem cells; HMGB3 high mobility group box 3; HO-1 heme oxygenase-1; NS normal saline; qRT-PCR quantitative real-time reverse transcription polymerase chain reaction; SBTx small bowel transplantation; ZO-1 Zona Occludens 1.

In the intestinal tissues of the SBTx rat model treated with stem cells, we scored the pathology according to the intensity of hematoxylin-eosin (HE) staining. The injury rejection score of small bowel allografts decreased significantly in the rats treated with HO-1/BMMSCs (Fig. 7a), and effectively reduced the apoptosis of IECs (Fig. 7b, c), which agreed with conclusion of our previous study^6,7^. We selected tissue from 3-day post-surgery rats for a follow-up study. The results of electron microscopy showed that the IECs and TJs of the small bowel in Sham group were basically intact. In the normal saline (NS) group, microvilli and TJs were twisted, broken, destroyed, and swollen. The intestinal tissue of the SBTx in the BMMSCs group was more intact than that in the NS group. The TJs in the small intestine of rats treated with HO-1/BMMSCs were basically normal, which is an important structural basis for the preservation of intestinal barrier function (Fig. 7d). Molecular analysis showed that the level of miR-200b in intestinal tissue of rats treated with HO-1/BMMSCs was significantly higher than that of other groups, while the HMGB3/JNK pathway was significantly downregulated, which reduced the inflammatory injury and apoptosis of the intestinal tissue (Figs. 7e–h and S9D).

## Discussion

Our previous studies confirmed that HO-1/BMMSCs have good protective and repair effects on small bowel allografts in vivo and vitro; however, the molecular mechanism has not been fully determined^10–13^. To systematically analyze the effect of HO-1/BMMSCs, we carried out transcriptome analysis. The results showed that HO-1 could change the mRNA expression profile of BMMSCs. HO-1 modification could improve the immune regulation, immune response, and resistance to stress stimulation of BMMSCs significantly, and affected many pathways, such as the TNF-α pathway. This provided a theoretical foundation for HO-1/BMMSCs to repair the inflammatory injury of IECs.

In our previous in vitro studies, we found that HO-1/BMMSCs could protect IECs model cells against TNF-α-induced-injury by reducing apoptosis, and protecting the structure of TJs and TJ proteins^19,20,28^. TJs are an important structural basis of intestinal mucosal barrier, which exists widely in normal IECs, and its integrity depends on the precise regulation of many proteins such as ZO-1^29,30^. After SBTx, the TJ structure and ZO-1 protein are seriously damaged, which is one of the reasons for the poor prognosis of surgical recipients of small bowel transplantation^9^. We further found that HO-1 enhanced the protective effect of BMMSCs against inflammatory injury of IECs after direct contact between isolated cells, suggesting that HO-1 modification can optimize the paracrine function of BMMSCs, thereby enhancing their protective effect^19,20^.

Exosomes are important vesicles secreted by cells that transmit information between cells. Studies have shown that the release of exosomes with specific genetic information (such as mRNAs and miRNAs) is an important mechanism by which MSCs exert their functions^31,32^. To clarify the role of exosomes derived from HO-1/BMMSCs, we isolated and purified the exosomes from the co-culture system, and confirmed their protective effect on the physiological activity of inflammatory injured IECs and the structure of intercellular TJs. These results indicated that HO-1 can change the expression profile of exosomes secreted by BMMSCs, and then transmit these alterations to IECs to regulate the molecular pathway in inflammation-injured cells to achieve protection.

Exosomes comprise a variety of molecules, and the mechanism of their action on targeted cells is complicated^33^. To ensure that only the proteins in IEC-6s were collected, we isolated the cells using 0.8-μm Transwell chambers and strictly removed all the cell culture supernatant in the process of cell-extraction. Through the analysis of the proteome directly responsible for their biological functions, we could predict the molecular mechanism by which the exosomes secreted by HO-1/BMMSCs protect IECs from inflammatory injury^34^. The results of the proteomic analysis showed that compared with the addition of natural BMMSC-derived exosomes, the addition of HO-1/BMMSCs exosomes changed the levels of various proteins in inflammatory injured IECs, which may be critical for the protective effect of HO-1/BMMSCs. We found that compared with the exosomes derived from natural BMMSCs, the exosomes derived from HO-1/BMMSCs could downregulate the level of HMGB3 in IECs, resulting in regulation of the JNK-mediated caspase-apoptosis pathway, which plays important roles in the injury process of IECs as reported previously^13,14,35^. We speculated that this might be one of the key mechanisms of the protective role of HO-1/BMMSCs and their exosomes.

Most of the studies on HMGB3 have focused on promoting the proliferation, invasion, and metastasis of non-small cell lung cancer, gastrointestinal tumors, and breast cancer; however, there are few studies in the non-tumor field^36–38^. HMGB3 is a member of the high mobility group protein family, which comprise nuclear non-histone proteins, including HMGBl, HMGB2, and HMGB3, and are damage-related molecular model molecules mediated by nucleic acid receptors. They are considered to be the universal “sentinel” of the immune response and play an important role in the inflammatory response^39^. Unlike HMGB1, HMGB3 is not actively secreted out of cells when they are damaged, but is passively released only when the cells are broken^40^. In our study, we found that the increased expression of HMGB3 in the cytoplasm of IECs stimulated by inflammation activates the mitogen-activated protein kinase JNK pathway, cleaves the linker region of caspase-3 to form cleaved caspase-3, and then induces cell apoptosis. HO-1/BMMSCs-derived exosomes could significantly reduce the HMGB3 level in the cytoplasm, and then downregulate the apoptosis induced by the p-JNK pathway and the degradation of ZO-1^35,41–43^. The protective effect of exosomes from HO-1/BMMSCs on the inflammatory injury of IECs depends on their downregulation of the abnormally high expression level of HMGB3 in the cytoplasm.

The method by which exosomes derived from HO-1/BMMSCs regulate the translation and expression of *Hmgb3* might be through the transfer of external miRNAs into IECs^44,45^. We analyzed nucleic acid sequences using the TargetScan, miRDB, and miRWalk databases and predicted miRNAs that might bind to the 3′ UTR region of *Hmgb3*. We selected miR-139, miR-17, miR-200b, miR-429, miR-191a, and miR-214a, which are involved in the process of intestinal injury in co-culture supernatant, for in vivo detection^46,47^. The results showed that compared with those in the BMMSC-treated group, the expression levels of miR-200b and miR-17 increased significantly in the HO-1/BMMSCs-treated group. The expression level of miR-200b showed a larger increase than that of miR-17. We then confirmed that miR-200b can bind to the 3′ UTR region of *Hmgb3* mRNA to inhibit its translation. A number of previous studies have shown that miR-200b can reduce the inflammatory injury of a variety of tissues and organs^48–54^. For example, Wendlandt et al.^48^ confirmed that miR-200b inhibits nerve inflammation by inhibiting inflammatory factors, such as interleukin (IL)-1 β, IL-6, and TNF-α to relieve neuropathic pain. Lo et al.^50^ confirmed that miR-200b attenuates the inflammatory injury of endothelial cells under high glucose via targeted regulation of O-GlcNAc transferase. Other studies have shown that miR-200b promotes the proliferation of IECs, inhibits mesenchymal transformation, and protects the structure of TJs through targeted regulation of Zinc finger E box binding protein-1/2, and by other mechanisms, which can effectively reduce the inflammatory injury in intestinal tissue. This represents a potential therapeutic strategy for intestinal diseases such as inflammatory bowel disease^49,51^. We carried out functional experiments by overexpressing (miR-200b mimic) and knocking down (miR-200b inhibitor) miR-200b in vitro to verify that it effectively protects the activity of IECs and the structural integrity of TJs by targeting *Hmgb3* and p-JNK pathway.

**Fig. 8:**
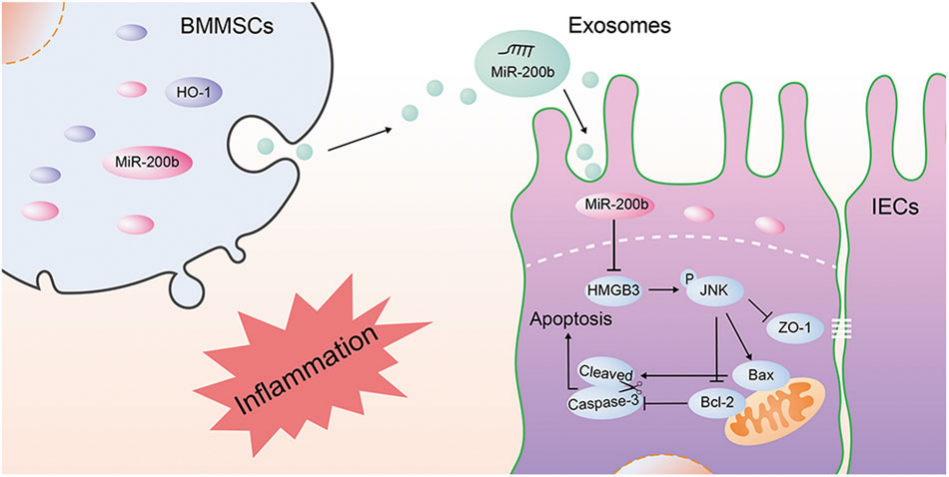
Schematic depiction of the mechanisms underlying miR-200b in HO-1/BMMSCs-derived exosomes alleviating inflammatory injury of IECs. BAX B-cell lymphoma-2 associated X, apoptosis regulator; BCL2 B-cell lymphoma-2 apoptosis regulator; BMMSCs Bone marrow mesenchymal stem cells; HMGB3 high mobility group box 3; HO-1 heme oxygenase-1; IECs intestinal epithelial cells; JNK C-Jun NH2-terminal kinase; ZO-1 Zona Occludens 1.

Next, we explored the source and mechanism of the upregulated miR-200b in the exosomes of the co-culture system. The contents and components of exosomes of MSCs are dynamic and are regulated by various factors^55–57^. HO-1 can regulate the expression of many kinds of miRNAs and miRNA processing enzymes. The mechanism might be related to HO-1 and downstream NF-E2-related factor 2 translocation to the nucleus and its effect on gene expression^58–61^. A study by Loboda et al.^62^ showed that cyclosporine A decreased the expression of miR-200b significantly in *Ho-1* (−/−) proximal tubular epithelial cells. By detecting the level of miR-200b in BMMSC cells and exosomes stimulated by TNF-α and HO-1 modification, we found that the expression level of miR-200b in BMMSCs with both HO-1 modification and TNF-α stimulation was significantly higher than that after single factor stimulation and in unstimulated BMMSCs. According to the available information, we speculated that HO-1/BMMSCs stimulated by inflammation might affect the expression or nuclear translocation of certain transcription factors that promote the transcription and secretion of miR-200b. Subsequently, miR-200b in the exosomes is transmitted into the inflammatory injured IECs, and binds to the 3′ UTR region of *Hmgb3*, which caused mRNA degradation, downregulation of the JNK-mediated apoptosis pathway, and finally, reduced inflammatory injury of IECs (Fig. 8). The molecular mechanism by which HO-1 regulates miR-200b in BMMSCs in the inflammatory environment will be the focus of our future research.

To further verify our conclusions, we conducted experiments in the intestinal tissue of the previously established in vivo rat model of SBTx immune rejection treated with HO-1/BMMSCs^7^. We found that compared with natural BMMSCs treatment, the degree of immune rejection of small bowel allografts in HO-1/BMMSCs-treated SBTx rats was reduced significantly. The results showed that HO-1/BMMSCs could reduce the apoptosis of small bowel allografts and the damage to the TJ structure. Previous studies confirmed that HO-1/BMMSCs and BMMSCs colonization of the transplanted small bowel tissues began at 3 day post surgery^6,7^. To minimize the protective effect of the quantity of colonizing cells, we selected tissue samples of transplanted small bowel at 3 day post surgery for detection. The results showed that the level of miR-200b in the small bowel allografts increased significantly and the level of HMGB3 decreased significantly in the HO-1/BMMSCs treatment group, which supported the conclusion of this study. However, it must be pointed out that our in vivo verification results are only preliminary. In future work, we plan to establish HO-1/BMMSCs-derived exosomes, miR-200b-overexpressing BMMSC-derived exosomes, and miR-200b-loaded exosomes to treat an allogeneic SBTx immune rejection rat model to further confirm the key role of miR-200b in the protection and repair of small intestinal tissue of SBTx rats.

In conclusion, the results of the present study showed that HO-1-modified BMMSCs exocrine secretion increased miR-200b expression in the inflammatory environment. The exosomes transfer miR-200b into IECs, where they bind and degrade *Hmgb3*, which downregulates the JNK pathway, resulting in reduced apoptosis of IECs and decreased damage to intercellular TJ structures, ultimately reducing the inflammatory damage to IECs.

## Materials and methods

### Isolation, identification, and HO-1-overexpression modification of BMMSCs

The rats used in our study were purchased from Vital River Company (Beijing, China). The experiment was performed according to National Institutes of Health Guide for the Care and Use of Laboratory animals (NIH Publications No. 8023, revised 1978) and approved by the Ethics Committee of Tianjin first Central Hospital (Permit number: 2016-03-A1). Bone marrow contents of the femur and tibia of Lewis rats were isolated under aseptic conditions, and BMMSCs were obtained using the continuous adherent method^6^. BMMSCs were cultured in 10% fetal bovine serum (FBS, Biowest, Nuaillé, France) + 1% penicillin-streptomycin combination (Gibco, Thermo Scientific, Waltham, MA, USA) + 89% Dulbecco’s modified Eagle’s medium (DMEM)/F12 (Gibco). When passaged to the 3rd generation, the BMMSCs were induced to differentiate to verify their adipogenic and osteogenic differentiation ability^63^. HO-1/BMMSCs and GFP/BMMSCs were obtained using adenovirus (Genechem, Shanghai, China) transfection, and the expression of GFP was observed using fluorescence microscopy (IX71, Olympus, Tokyo, Japan).

### Isolation and identification of exosomes

Cell culture supernatants for exosomes isolation were substituted by exosome-free medium (System Biosciences, Palo Alto, CA, USA) 2 days before exosomes isolation. Exosome separation kits (System Biosciences) were used to isolate exosomes in accordance with manufacturer’s instructions. The identification process of the exosomes included the observation of exosomes by electron microscopy (Hitachi Hmur600, Tokyo, Japan), determination of the grain-diameter ratio, and determination of the concentration of the exosomes using NTA (ZetaVIEW S/N 17–310, PARTICLE METRIX, Meerbusch, Germany). Western blotting was used to detect the exosome proteins CD63, CD9, and TSG 101 as positive markers and Calnexin as a purity control^64,65^.

### Establishment of an in vitro IEC-6 cell model of inflammatory injury

The rat IEC-6 cell line was maintained in our laboratory^20^. The IEC-6 cell model of inflammatory injury in vitro was achieved by treatment with TNF-α (100 ng/mL) and lymphocytes. The method of lymphocyte extraction and the generation of the BMMSCs (including HO-1/BMMSCs) protection model were as performed according to our previous study^19,20^. Lymphocytes, BMMSCs, and IEC-6s were isolated using a 0.8-µm Transwell chamber (Corning, Sigma-Aldrich, St. Louis, MO, USA). To clarify the role of the exosomes, we collected and purified the exosomes from the culture supernatants of each group and added them to the corresponding groups at 100 μg/mL (according to the exosome protein concentration) after the inflammatory injury of IEC-6s.

### MiRNA mimic and inhibitor transfection

Cells were cultured to the sub-fusion state (>60% confluence). Lipofectamine 3000 (Invitrogen, Carlsbad, CA, USA) was diluted with Opti-MEM medium (Gibco) and added to the cells, and then the miR-200b-3p mimic (Sense: 5′-UAAUACUGCCUGGUAAUGAUGAC-3′, Antisense: 5′-CAUCAUUACCAGGCAGUAUUAUU-3′) and miR-200b-3p inhibitor (Sense: 5′- GUCAUCAUUACCAGGCAGUAUUA-3′) were added^66^. The expression of miR-200b in the transfected cells was detected using qRT-PCR to confirm the effect after 24 h.

### Treatment of rat allogeneic SBTx in vivo model with BMMSCs and HO-1/BMMSCs

The rat allogeneic SBTx model used in this study was established using Lewis rats as the donors and Brown Norway rats as the recipients, and the methods and principles of establishing the model were described in our previous studies^6,7^. According to the different treatments used, the rats were divided into four groups: Sham group (without SBTx), NS group (treated with normal saline), BM group (treated with BMMSCs) and the HO-1/BMMSCs group (treated with HO-1/BMMSCs). HE staining was used to observe the morphology of the intestinal tissue and evaluate the score of intestinal injury^67^. Transmission electron microscopy was used to observe the tissue ultrastructure, and immunohistochemistry (IHC) was used to label proteins (HMGB3 and ZO-1) in intestinal tissues^7,68^.

### Extraction of RNA, qRT-PCR, and RNA-seq

The Trizol Reagent (Takara, Shiga, Japan) was used to extract the intracellular RNA, and Exosome RNA purification column kits (System Biosciences) were used to purify the exosomal RNA. Reverse transcription and qPCR were performed according to the instructions of the kits used. The β-actin gene (*Actb*) was used as a control for normalization for mRNA and U6 was used for normalization for miRNAs. Information on the primers is shown in Table S2 and S3.

RNA-seq was performed by MajorBio, Inc. (Shanghai, China). The RNA concentration was quantified using a NanoDrop spectrophotometer (Thermo Scientific), and the RNA integrity was evaluated using a 2100 bioanalyzer and RNA 6000 kit (Agilent Technologies, Santa Clara, CA, USA). The RNA-seq transcriptome library was prepared following the instructions of the TruSeq™ RNA sample preparation Kit from Illumina (San Diego, CA, USA) using 1 μg of total RNA. Briefly, mRNA was isolated according to the polyA selection method using oligo (dT) beads and then fragmented using fragmentation buffer. Double-stranded cDNA was then synthesized using a SuperScript double-stranded cDNA synthesis kit (Invitrogen) with random hexamer primers (Illumina). The synthesized cDNA was subjected to end-repair, phosphorylation, and “A” base addition according to Illumina’s library construction protocol. The libraries were size selected for cDNA target fragments of 300 bp on 2% Low Range Ultra Agarose followed by PCR amplification using Phusion DNA polymerase (NEB, Ipswich, MA, USA) for 15 PCR cycles. After quantification using a TBS380 minifluorimeter (the cDNA solution was adjusted to 100 nM), the paired-end RNA-seq sequencing library was sequenced using an Illumina HiSeqxten/NovaSeq 6000 sequencer (2 × 150 bp read length). The raw paired end reads were trimmed and subjected to quality control using SeqPrep (https://github.com/jstjohn/SeqPrep) and Sickle (https://github.com/najoshi/sickle) with default parameters. To identify DEGs between two samples, the expression level of each transcript was calculated according to the transcripts per million reads (TPM) method. Essentially, differential expression analysis was performed using DEGseq with a Q value ≤ 0.05. DEGs with fold change > 2 or <−2 and a Q value ≤ 0.001 were considered to be significant DEGs^69^. Functional-enrichment analysis, including GO and KEGG, were performed to identify which DEGs were significantly enriched in GO terms and metabolic pathways using a Bonferroni-corrected *P*-value ≤ 0.05 compared with the whole-transcriptome background. GO functional enrichment and KEGG pathway analysis were carried out using Goatools (https://github.com/tanghaibao/Goatools) and KOBAS (http://kobas.cbi.pku.edu.cn/home.do)^70^.

### Western blotting and label-free quantitative proteomic analysis

Total intracellular proteins were extracted using Radioimmunoprecipitation assay Lysis Buffer (Solarbio, Beijing, China). The detailed experimental method for bicinchoninic acid assay, western blotting and quantification can be found in our previous publication^20^. β-actin was used as a control for normalization of the protein levels.

Label-free quantitative proteomic analysis of IEC-6s was performed by PTM-BioLab, Inc. (Zhejiang, China). Cell samples were sonicated three times on ice using a high intensity ultrasonic processor in lysis buffer (8 M urea, 1% protease inhibitor cocktail). The protein solution was reduced using 5 mM dithiothreitol for 30 min at 56 °C, alkylated using 11 mM iodoacetamide for 15 min at room temperature in the dark, diluted to a urea concentration <2 M, and digested using trypsin. The resulting peptides were subjected to nanospray ionization source followed by tandem mass spectrometry (MS/MS) in Q Exactive™ Plus (Thermo Scientific) coupled online to the ultra-performance liquid chromatography instrument (ARKRAY, Kyoto, Japan). The m/z scan range was 350–1800 for a full scan, and intact peptides were detected in the Orbitrap at a resolution of 70,000. Peptides were then selected for MS/MS using a new chemical entity setting of 28 and the fragments were detected in the Orbitrap at a resolution of 17,500. A data-dependent procedure that alternated between one MS scan followed by 20 MS/MS scans with 15.0 s dynamic exclusion was implemented. The resulting MS/MS data were processed using Maxquant search engine (v.1.5.2.8, Computational Systems Biochemistry, Martinsried, Germany). Tandem mass spectra were searched against the *Uniprot_Rattus_norvegicus_10116\20181030* database concatenated with a reverse decoy database. After the DAPs were obtained, GO and KEGG analyses were performed similarly to the DEGs.

### Prediction and identification of miRNA-mRNA targeting relationships

TargetScan (http://www.targetscan.org/), miRWalk (http://mirwalk.umm.uni-heidelberg.de/), and miRDB (http://mirdb.org/) analysis were performed to predict the targeting relationship between miRNAs and mRNAs. Dual luciferase reporter assays were used to verify the relationship^71^. The miRNA-WT and MUT plasmids (Genepharma, Inc., Jiangsu, China) were constructed according to the target sequence. The relative fluorescence intensities of firefly and Renilla luciferase were calculated. IEC-6s and competent HEK293T cells were transformed with the miRNA plasmids (Fig. S4A).

### FCM

The molecular surface markers of BMMSCs, CD29, CD34, CD45, CD90, RT1-A, and RT1-B (Table S1) were identified using FCM (Accuri C6 Plus, Becton, Dickinson and Company, NJ, USA)^68^. For FCM, an Annexin V-fluorescein isothiocyanate (FITC)/ PI-phycoerythrin (PE) apoptosis detection kit (Solarbio) was used according to the manufacturer’s instructions.

### IF, IHC, and TUNEL staining

The IF, IHC, and TUNEL staining was performed according to our previous studies^6,19,20^. A TUNEL-TMR red kit (Roche, Sigma-Aldrich) was used to label apoptotic cells in vitro, and a TUNEL-Peroxidase kit (Roche) was used to assess paraffin sections of SBTx model tissues in vivo^7^. Hematoxylin or 4,6-diamino-2-phenyl indole (DAPI) were used to label cell nuclei.

### Ultrastructure observation

The morphology of exosomes and SBTx tissues was detected by transmission electron microscopy. Fresh exosomes samples were dissolved in phosphate-buffered saline and transplanted intestinal tissue samples were cut into 1 mm × 1 mm × 2 mm pieces. The samples were fixed with 2.5% glutaraldehyde solution, embed, subjected to ultrathin sectioning, and then observed under a transmission electron microscope^68^.

### Cell Counting Kit-8

Cell Counting Kit-8 (CCK-8) (Solarbio) was used to detect cell viability after injury and treatment (5000 cells/tube). The experiment was carried out according to the manufacturer’s instructions^20^.

### Statistical analysis

All statistical analyses were performed using SPSS version 17.0 (IBM Corp., Armonk, NY, USA). Normally distributed data were expressed as the mean ± standard deviation. The number of independent experiments performed are indicated in the figure legends. Significant differences between groups were assessed using two-tailed Student’s T test (individual comparison) or using analysis of variance with minimal significant difference and Student-Newman-Keuls comparison. A difference with *P* < 0.05 was considered statistically significant. GraphPad Prism 8.0 software (GraphPad Software Inc., La Jolla, CA, USA) was used to display the significant data.

## Supplementary information

Supplemental tables

Supplemental figure legends

Supplemental figure 1

Supplemental figure 2

Supplemental figure 3

Supplemental figure 4

Supplemental figure 5

Supplemental figure 6

Supplemental figure 7

Supplemental figure 8

Supplemental figure 9
