# Supplementary material for: MiR-200b in heme oxygenase-1-modified bone marrow mesenchymal stem cell-derived exosomes alleviates inflammatory injury of intestinal epithelial cells by targeting high mobility group box 3
Source: Cell Death Dis. 2020 Jun 25;11(6):480. doi: 10.1038/s41419-020-2685-8 (PMC7316799; doi:10.1038/s41419-020-2685-8)
Supplement: Supplementary file 1 — Supplemental tables [file 41419_2020_2685_MOESM1_ESM.docx]

**Supplemental Table 1. List of primary antibodies used in the study.**

| Antibody | Applications | Catalog number | Company | Location |
| --- | --- | --- | --- | --- |
| CD29 | FCM | 303006 | Biolegend | CA, USA |
| CD34 | FCM | 343604 | Biolegend | CA, USA |
| CD45 | FCM | 368510 | Biolegend | CA, USA |
| CD90 | FCM | 206106 | Biolegend | CA, USA |
| RT1-A | FCM | 205208 | Biolegend | CA, USA |
| RT1-B | FCM | 205305 | Biolegend | CA, USA |
| HO-1 | WB (1:600), IF (1:250) | 10701-1-AP | Proteintech Group | IL, USA |
| CD9 | WB (1:1000) | 20597-1-AP | Proteintech Group | IL, USA |
| TSG 101 | WB (1:1000) | 14497-1-AP | Proteintech Group | IL, USA |
| CD63 | WB (1:800) | 25682-1-AP | Proteintech Group | IL, USA |
| Calnexin | WB (1:800) | 10427-2-AP | Proteintech Group | IL, USA |
| ZO-1 | WB (1:2000), IF (1:150), IHC (1:250) | 61-7300 | Invitrogen | MA，USA |
| Caspase 3 | WB (1:1000) | 9662 | Cell Signaling Technology | MA, USA |
| BAX | WB (1:1000) | 50599-2-Ig | Proteintech Group | IL, USA |
| BCL2 | WB (1:1000) | 26593-1-AP | Proteintech Group | IL, USA |
| HMGB3 | WB (1:500), IF (1:150), IHC (1:150) | orb455136 | Biorbyt | CB4, UK |
| p-JNK | WB (1:1000) | 9255 | Cell Signaling Technology | MA, USA |
| JNK | WB (1:1000) | 9252 | Cell Signaling Technology | MA, USA |
| β-actin | WB (1:2000) | 14395-1-AP | Proteintech Group | IL, USA |

**Abbreviations:** WB, western blotting, IHC, Immunohistochemistry, IF, Immunofluorescence, FCM, Flow Cytometer

**Supplemental Table 2. PCR primer sequences for mRNA.**

| Gene (mRNA) | Primer Sequence (5'-3') | Product Size (bp) |
| --- | --- | --- |
| *Ho-1* (Forword) | GCCCACGCATATACCCGCTAC | 212 |
| *Ho-1* (Reverse) | TCTGTCACCCTGTGCTTGACC |  |
| *Ifit3* (Forword) | ACTCTGAGCACAAAGCGACA | 136 |
| *Ifit3* (Reverse) | ATCTCTGCTCGATCACTGCG |  |
| *Akap2* (Forword) | CACGGAACTGGCGCCTAGAG | 152 |
| *Akap2* (Reverse) | CACGGACACCCCAATTTCCA |  |
| *Nlrc5* (Forword) | GAGTGTCAGCTGGAATCCCT | 144 |
| *Nlrc5* (Reverse) | GGTCAATGGAGGAAGGCAAGA |  |
| *Rgs7* (Forword) | TGGCACCTTCTACCGGTTTC | 145 |
| *Rgs7* (Reverse) | GCTCTCGGCTTCGTAGTCTG |  |
| *Cdh1* (Forword) | CCTTGCCTACCTCTGTGACG | 144 |
| *Cdh1* (Reverse) | CAAGAGCTGATCACCCGGAG |  |
| *Sfrp2* (Forword) | GGGCCAGAGAGAGTTCAAGC | 164 |
| *Sfrp2* (Reverse) | AGACTGTAGCTCTCCCGACA |  |
| *Hmgb3* (Forword) | ACTTTGCTGTTGGGGTGACA | 184 |
| *Hmgb3* (Reverse) | CACGCCAAACAAGGAGCATC |  |
| *Jnk* (Forword) | TCCAGTTCTCGTACCCGCTA | 135 |
| *Jnk* (Reverse) | AGCATGGCGTGACACAGTAA |  |
| *β-actin* (Forword) | GCGTGACATTAAAGAGAAGCTG | 500 |
| *β-actin* (Reverse) | AGAAGCATTTGCGGTGCAC |  |

**Supplemental Table 3. PCR primer sequences for microRNA.**

| Gene (miRNA) | RT-primer | Sense primer | Antisense primer |
| --- | --- | --- | --- |
| rno-miR-200b | GTCGTATCCAGTGCAGGGTCCGAGGTGCACTGGATACGACGTCATCA | TGCGGTAATACTGCCTGGTAATG | CCAGTGCAGGGTCCGAGGT |
| rno-miR-17 | GTCGTATCCAGTGCAGGGTCCGAGGTGCACTGGATACGACCCACAAG | TGCGGACTGCAGTGAAGGCACT |  |
| rno-miR-139 | GTCGTATCCAGTGCAGGGTCCGAGGTGCACTGGATACGACCTCCAAC | TGCGGTGGAGACGCGGCCCTGT |  |
| rno-miR-429 | GTCGTATCCAGTGCAGGGTCCGAGGTGCACTGGATACGACACGGCAT | TGCGGTAATACTGTCTGGTAATG |  |
| rno-miR-191a | GTCGTATCCAGTGCAGGGTCCGAGGTGCACTGGATACGACGGGAACG | TGCGGGCTGCACTTGGATTTCG |  |
| rno-miR-214a | GTCGTATCCAGTGCAGGGTCCGAGGTGCACTGGATACGACCTGCCTG | TGCGGACAGCAGGCACAGACAG |  |
| U6 | GTCGTATCCAGTGCAGGGTCCGAGGTGCACTGGATACGACAAAATATGG | TGCGGGTGCTCGCTTCGGCAGCAC |  |

**Supplemental Table 4. Different expressed genes between HO-1/BMMSCs and GFP/BMMSCs.**

| Gene ID | Gene name | TPM (HO-1/BMMSCs) | TPM (GFP /BMMSCs) | log2FC （HB/GB） |
| --- | --- | --- | --- | --- |
| ENSRNOG00000046600 | *AABR07015066.1* | 1625.54 | 3445.12 | -1.30081 |
| ENSRNOG00000059586 | *AABR07015080.2* | 747.16 | 2047.7 | -1.30196 |
| ENSRNOG00000060518 | *LOC257642* | 446.98 | 928.05 | -1.26314 |
| ENSRNOG00000003217 | *Lgals3bp* | 280.63 | 122.03 | 1.050423 |
| ENSRNOG00000006228 | *Pdia4* | 609.16 | 202.9 | 1.435917 |
| ENSRNOG00000014117 | *Hmox1* | 8281.83 | 258.55 | 4.844092 |
| ENSRNOG00000017414 | *Irf7* | 84.28 | 13.05 | 2.551821 |
| ENSRNOG00000026963 | *Hsp90b1* | 736.41 | 258.96 | 1.35852 |
| ENSRNOG00000029658 | *Rnf213* | 13.97 | 2.34 | 2.379642 |
| ENSRNOG00000037198 | *Usp18* | 73.61 | 8.69 | 2.928042 |
| ENSRNOG00000056747 | *AABR07015055.1* | 277.69 | 542.81 | -1.14221 |
| ENSRNOG00000023410 | *Apol9a* | 54.03 | 4.49 | 3.431081 |
| ENSRNOG00000049575 | *Atn1* | 33.9 | 8.67 | 1.82327 |
| ENSRNOG00000004659 | *Creld2* | 168.34 | 51.84 | 1.535497 |
| ENSRNOG00000027008 | *Igtp* | 35.62 | 5.26 | 2.554812 |
| ENSRNOG00000023334 | *Parp14* | 13.01 | 2.71 | 2.119087 |
| ENSRNOG00000001963 | *Mx2* | 20.1 | 0.96 | 4.235442 |
| ENSRNOG00000056869 | *AABR07015002.1* | 17.45 | 0.54 | 4.655992 |
| ENSRNOG00000013267 | *Helz2* | 7.08 | 1.42 | 2.185548 |
| ENSRNOG00000000500 | *Scube3* | 29.3 | 12.37 | 1.103816 |
| ENSRNOG00000047350 | *LOC100909505* | 0 | 29.86 | -10.7388 |
| ENSRNOG00000019050 | *Ifit1* | 20.31 | 1.01 | 4.178739 |
| ENSRNOG00000022256 | *Cxcl10* | 52.12 | 4.92 | 3.238655 |
| ENSRNOG00000028243 | *Derl3* | 109.71 | 3.76 | 4.48217 |
| ENSRNOG00000046414 | *LOC108348048* | 8.78 | 0.13 | 5.923225 |
| ENSRNOG00000028814 | *Oasl2* | 24.05 | 4.86 | 2.16024 |
| ENSRNOG00000018434 | *Stab1* | 5.14 | 12.31 | -1.40318 |
| ENSRNOG00000008012 | *Abcb1a* | 24.8 | 8.74 | 1.348007 |
| ENSRNOG00000021802 | *Isg15* | 36.48 | 3.73 | 3.128319 |
| ENSRNOG00000023463 | *Parp9* | 16.77 | 3.09 | 2.273716 |
| ENSRNOG00000001959 | *Mx1* | 12.39 | 1.38 | 3.032445 |
| ENSRNOG00000057092 | *Slfn4* | 13.3 | 3.16 | 1.962256 |
| ENSRNOG00000061740 | *Smarcb1* | 23.92 | 7.72 | 1.486425 |
| ENSRNOG00000021719 | *Slfn5* | 16.73 | 6.83 | 1.651066 |
| ENSRNOG00000055154 | *AABR07063462.1* | 0 | 3279.21 | -12.3705 |
| ENSRNOG00000036604 | *Ifit2* | 15.93 | 3.59 | 2.004871 |
| ENSRNOG00000033220 | *Oas1b* | 21.81 | 2.86 | 2.783293 |
| ENSRNOG00000007539 | *Rsad2* | 6.24 | 0.06 | 6.455104 |
| ENSRNOG00000001369 | *Oas1a* | 17.18 | 1.55 | 3.540485 |
| ENSRNOG00000011078 | *Srm* | 68.86 | 20.71 | 1.569244 |
| ENSRNOG00000004346 | *Notch3* | 3.65 | 8.95 | -1.43598 |
| ENSRNOG00000001187 | *Oasl* | 10.97 | 1.37 | 2.853068 |
| ENSRNOG00000031081 | *Stat2* | 31.13 | 13.72 | 1.042339 |
| ENSRNOG00000007159 | *Ccl2* | 136.96 | 48.41 | 1.316973 |
| ENSRNOG00000010517 | *Fam126a* | 20.17 | 8.85 | 1.012472 |
| ENSRNOG00000000777 | *RT1-S3* | 36.11 | 10.26 | 1.72536 |
| ENSRNOG00000002341 | *Trim25* | 27.24 | 8.98 | 1.451552 |
| ENSRNOG00000055498 | *LOC103694875* | 44.73 | 2.27 | 4.14066 |
| ENSRNOG00000023400 | *Dtx3l* | 11.29 | 4.45 | 3.202927 |
| ENSRNOG00000028895 | *Rtp4* | 11.74 | 0.9 | 3.557613 |
| ENSRNOG00000002468 | *Tnr* | 15.43 | 6.57 | 1.089302 |
| ENSRNOG00000011504 | *Akap2* | 7.73 | 1.23 | 3.586933 |
| ENSRNOG00000059504 | *AABR07015078.2* | 397.09 | 707.04 | -1.15418 |
| ENSRNOG00000007690 | *Cmpk2* | 5.59 | 0.69 | 2.875789 |
| ENSRNOG00000052444 | *RGD1563091* | 4.16 | 0.66 | 2.593523 |
| ENSRNOG00000028335 | *Fat4* | 4.87 | 2.17 | 1.025557 |
| ENSRNOG00000037113 | *Slfn2* | 26.8 | 8.04 | 1.581539 |
| ENSRNOG00000050193 | *LOC100912262* | 0 | 4.31 | -8.98887 |
| ENSRNOG00000027191 | *Birc6* | 7.68 | 3.54 | 1.020423 |
| ENSRNOG00000008196 | *Parp12* | 25.23 | 10.45 | 1.14627 |
| ENSRNOG00000048315 | *Eif2ak2* | 11.23 | 3.61 | 1.493377 |
| ENSRNOG00000018247 | *Dhx58* | 11.67 | 2.56 | 2.045714 |
| ENSRNOG00000019741 | *Isyna1* | 22.92 | 7.36 | 1.486286 |
| ENSRNOG00000008194 | *Znfx1* | 9.56 | 4.1 | 1.079114 |
| ENSRNOG00000031138 | *Irgm* | 16.93 | 4.86 | 1.650343 |
| ENSRNOG00000016164 | *Fcrl2* | 25.55 | 46.92 | -1.03484 |
| ENSRNOG00000018414 | *Csf1r* | 10.19 | 19.09 | -1.03078 |
| ENSRNOG00000022218 | *Ifi44* | 12.49 | 2.01 | 2.505145 |
| ENSRNOG00000060896 | *AABR07063424.1* | 173.02 | 337.47 | -1.2653 |
| ENSRNOG00000027151 | *Lrrc58* | 42.36 | 15.9 | 1.253074 |
| ENSRNOG00000022839 | *Ifit3* | 7.11 | 0.56 | 3.519645 |
| ENSRNOG00000019478 | *Irf9* | 30.18 | 10.67 | 1.342665 |
| ENSRNOG00000006227 | *Ifih1* | 4.71 | 0.77 | 2.47132 |
| ENSRNOG00000056028 | *Rfxap* | 8.6 | 0 | 8.577095 |
| ENSRNOG00000050669 | *LOC100911515* | 27.68 | 69.32 | -1.29589 |
| ENSRNOG00000047076 | *Oas1g* | 12.77 | 2.18 | 2.392673 |
| ENSRNOG00000023068 | *Cd5l* | 8.27 | 18.81 | -1.33729 |
| ENSRNOG00000028382 | *Rfxapl1* | 0 | 6.47 | -8.29414 |
| ENSRNOG00000003927 | *Cd55* | 21.18 | 8.81 | 1.108582 |
| ENSRNOG00000008465 | *Tmem176b* | 4.77 | 16.77 | -2.13688 |
| ENSRNOG00000037563 | *Cd68* | 24.81 | 45.9 | -1.05125 |
| ENSRNOG00000038960 | *RGD1309362* | 8.18 | 2.19 | 1.75391 |
| ENSRNOG00000051922 | *Samd9l* | 7.45 | 2.71 | 1.315585 |
| ENSRNOG00000059900 | *Bst2* | 50.39 | 9.03 | 2.230682 |
| ENSRNOG00000050374 | *Pigg* | 0 | 1.55 | -8.10149 |
| ENSRNOG00000004006 | *Dnajb9* | 20.95 | 8.33 | 1.178065 |
| ENSRNOG00000011054 | *Laptm5* | 7.84 | 16.42 | -1.22485 |
| ENSRNOG00000032240 | *Gbp5* | 4.82 | 0.93 | 2.235538 |
| ENSRNOG00000030963 | *LOC108351137* | 8.24 | 20.88 | -1.50377 |
| ENSRNOG00000038047 | *Mt1* | 125.99 | 19.99 | 2.338259 |
| ENSRNOG00000031743 | *Gbp2* | 6.87 | 1.69 | 1.877114 |
| ENSRNOG00000010975 | *Adnp* | 3.61 | 1.1 | 1.554555 |
| ENSRNOG00000058439 | *Fam50a* | 0 | 5.83 | -7.74336 |
| ENSRNOG00000060066 | *Hoxa6* | 2.97 | 0 | 7.730111 |
| ENSRNOG00000059097 | *Ddx60* | 1.58 | 0.06 | 4.654227 |
| ENSRNOG00000000239 | *Ccl7* | 50.8 | 19.48 | 1.200252 |
| ENSRNOG00000006384 | *Ddx58* | 18.16 | 3.11 | 1.674731 |
| ENSRNOG00000019430 | *Coro1a* | 6.89 | 15.73 | -1.3466 |
| ENSRNOG00000043098 | *Mt2A* | 205.23 | 53.91 | 1.605141 |
| ENSRNOG00000023453 | *Lrba* | 6.22 | 2.76 | 1.174188 |
| ENSRNOG00000022082 | *Dusp11* | 7.47 | 0 | 7.677691 |
| ENSRNOG00000029195 | *Uba7* | 2.34 | 0.34 | 2.529167 |
| ENSRNOG00000020142 | *Gdf1* | 0 | 2.17 | -7.50688 |
| ENSRNOG00000055765 | *LOC103694863* | 5.34 | 9.58 | -1.06683 |
| ENSRNOG00000014844 | *Kif21a* | 5.63 | 2.19 | 1.407621 |
| ENSRNOG00000023708 | *Tmem176a* | 2.89 | 11.83 | -2.20221 |
| ENSRNOG00000052981 | *AABR07058464.1* | 12.51 | 1.77 | 2.644382 |
| ENSRNOG00000061059 | *LOC100912478* | 2.63 | 0.67 | 9.054142 |
| ENSRNOG00000018517 | *Trim21* | 7.42 | 0.6 | 2.088024 |
| ENSRNOG00000058083 | *Metazoa_SRP* | 25.07 | 165.29 | -3.13061 |
| ENSRNOG00000061096 | *Rn7sl1* | 25.07 | 165.29 | -3.13061 |
| ENSRNOG00000013578 | *Trem2* | 5.63 | 15.85 | -1.76164 |
| ENSRNOG00000049814 | *LOC100910882* | 15.82 | 6.74 | 1.077069 |
| ENSRNOG00000012807 | *C1qa* | 13.57 | 26.9 | -1.15747 |
| ENSRNOG00000049661 | *Inafm1* | 5.45 | 0.63 | 2.944213 |
| ENSRNOG00000050535 | *LOC108348096* | 1.29 | 0 | 7.293641 |
| ENSRNOG00000049895 | *LOC100910143* | 4.92 | 2.1 | 1.084583 |
| ENSRNOG00000011971 | *C1s* | 9.55 | 4.08 | 1.086595 |
| ENSRNOG00000048771 | *RGD1559482* | 6.29 | 18.86 | -1.73304 |
| ENSRNOG00000037744 | *Oas1i* | 8.86 | 1.08 | 2.690221 |
| ENSRNOG00000020845 | *Tyrobp* | 26.31 | 54.62 | -1.27155 |
| ENSRNOG00000029191 | *LOC685067* | 1.93 | 0.07 | 4.630676 |
| ENSRNOG00000024899 | *Cxcl13* | 19.09 | 6.46 | 1.390542 |
| ENSRNOG00000037709 | *Armcx1* | 0 | 2.59 | -7.09731 |
| ENSRNOG00000002470 | *Ifi47* | 6.23 | 1.51 | 1.890063 |
| ENSRNOG00000025764 | *AC128848.1* | 50.85 | 14.26 | 1.601718 |
| ENSRNOG00000017854 | *Ucp2* | 6.77 | 13.49 | -1.15197 |
| ENSRNOG00000008409 | *Myo1f* | 2.62 | 5.13 | -1.1145 |
| ENSRNOG00000050806 | *Spty2d1* | 4.75 | 1.99 | 1.111942 |
| ENSRNOG00000022769 | *Sp100* | 9.48 | 3.75 | 1.09575 |
| ENSRNOG00000000916 | *Katnal1* | 2.64 | 0 | 7.005484 |
| ENSRNOG00000003160 | *RragB* | 0 | 0.93 | -6.90848 |
| ENSRNOG00000005285 | *Fbxo33* | 6.53 | 3.06 | 1.017699 |
| ENSRNOG00000015318 | *Heyl* | 0.23 | 1.22 | -2.569 |
| ENSRNOG00000022572 | *LOC102548286* | 2.34 | 10.01 | -2.27699 |
| ENSRNOG00000049770 | *Cryba4* | 8.85 | 20.37 | -1.42746 |
| ENSRNOG00000019890 | *Folr2* | 6.34 | 14.57 | -1.40539 |
| ENSRNOG00000034084 | *Ggct* | 29.72 | 11.12 | 1.234291 |
| ENSRNOG00000024159 | *Fcer1g* | 26.61 | 49.7 | -1.11255 |
| ENSRNOG00000007975 | *Ncoa2* | 4.33 | 1.78 | 1.137764 |
| ENSRNOG00000027246 | *LOC100911668* | 3.26 | 0.75 | 1.970321 |
| ENSRNOG00000037371 | *XAF1* | 12.17 | 2.07 | 2.367642 |
| ENSRNOG00000004361 | *Parp10* | 7.01 | 2.93 | 1.111648 |
| ENSRNOG00000021984 | *Rgs7* | 0.05 | 1.43 | -4.79752 |
| ENSRNOG00000028043 | *Cxcl3* | 5.23 | 0.28 | 3.952604 |
| ENSRNOG00000032348 | *Rpl35al1* | 0 | 53.12 | -6.72644 |
| ENSRNOG00000019351 | *LOC100911881* | 1.99 | 6 | -1.75929 |
| ENSRNOG00000042905 | *RT1-T24-4* | 79.87 | 15.25 | 1.103338 |
| ENSRNOG00000049422 | *LOC108348047* | 10.56 | 19.01 | -1.02772 |
| ENSRNOG00000011585 | *Fat3* | 1.37 | 0.61 | 1.026477 |
| ENSRNOG00000010438 | *Cpt1b* | 7.07 | 2.25 | 1.245544 |
| ENSRNOG00000000699 | *Selplg* | 2.37 | 5.62 | -1.40035 |
| ENSRNOG00000002120 | *Spata18* | 4.7 | 1.93 | 1.171244 |
| ENSRNOG00000061910 | *Igfbp3* | 9.03 | 4.06 | 1.004028 |
| ENSRNOG00000057696 | *LOC103690016* | 0 | 0.72 | -6.61848 |
| ENSRNOG00000048917 | *LOC100909604* | 0.48 | 2.15 | -2.31155 |
| ENSRNOG00000018808 | *Vip* | 12 | 4.74 | 1.183217 |
| ENSRNOG00000011984 | *Cxcl14* | 13.56 | 5.93 | 1.03566 |
| ENSRNOG00000027271 | *RGD1359290* | 11.38 | 5.05 | 1.050686 |
| ENSRNOG00000058793 | *Vma21* | 0 | 2.21 | -6.59525 |
| ENSRNOG00000003576 | *Ints2* | 4.4 | 1.99 | 1.002645 |
| ENSRNOG00000051002 | *RT1-DMb* | 2.49 | 8.72 | -1.76492 |
| ENSRNOG00000059284 | *AABR07015674.1* | 2.89 | 0.07 | 5.277735 |
| ENSRNOG00000046779 | *LOC100911562* | 3.5 | 0.69 | 2.383949 |
| ENSRNOG00000037167 | *Rtp3* | 3.1 | 0.65 | 2.125441 |
| ENSRNOG00000022565 | *Lrrc25* | 2.73 | 5.68 | -1.21063 |
| ENSRNOG00000045924 | *RT1-T24-3* | 9.44 | 2.59 | 1.690018 |
| ENSRNOG00000030704 | *AC094053.1* | 0.61 | 0 | 6.523067 |
| ENSRNOG00000007679 | *Cyth4* | 2.31 | 4.96 | -1.26522 |
| ENSRNOG00000001971 | *Bbx* | 4.55 | 1.52 | 1.29933 |
| ENSRNOG00000055229 | *LOC108348118* | 4.01 | 1.53 | 1.245208 |
| ENSRNOG00000004890 | *Adcy8* | 1.89 | 0.57 | 1.587283 |
| ENSRNOG00000005378 | *Gna15* | 0.51 | 1.6 | -1.80745 |
| ENSRNOG00000050419 | *Avil* | 2.96 | 0.9 | 1.576228 |
| ENSRNOG00000031579 | *LOC100363469* | 1.51 | 15.9 | -3.66018 |
| ENSRNOG00000003802 | *Pttg1* | 13.5 | 26.01 | -1.13088 |
| ENSRNOG00000013213 | *Epha4* | 1.24 | 0.37 | 1.607025 |
| ENSRNOG00000008880 | *Nrk* | 4.54 | 2.57 | 1.450031 |
| ENSRNOG00000012390 | *Npw* | 13.23 | 33.51 | -1.38818 |
| ENSRNOG00000037462 | *Eml6* | 0.88 | 0.21 | 1.925419 |
| ENSRNOG00000002950 | *Lyl1* | 0.48 | 2.89 | -2.73856 |
| ENSRNOG00000037339 | *Siglec10* | 2.35 | 4.6 | -1.1946 |
| ENSRNOG00000005059 | *Med22* | 0.74 | 2.02 | -1.60043 |
| ENSRNOG00000048222 | *Nlrc5* | 0.66 | 0.09 | 2.73377 |
| ENSRNOG00000061155 | *LOC100911851* | 2.11 | 4.5 | -1.19854 |
| ENSRNOG00000050395 | *Ms4a6e* | 0.58 | 3.1 | -2.56567 |
| ENSRNOG00000002318 | *Limch1* | 2.68 | 1.17 | 1.026434 |
| ENSRNOG00000042139 | *Clec4a1* | 2.91 | 6.4 | -1.29471 |
| ENSRNOG00000058555 | *7SK* | 5.7 | 45.13 | -3.35832 |
| ENSRNOG00000046867 | *LOC102549115* | 0 | 1.5 | -6.14609 |
| ENSRNOG00000014653 | *Arl11* | 3.46 | 7.4 | -1.25385 |
| ENSRNOG00000026407 | *Fam184a* | 3.56 | 1.52 | 1.083188 |
| ENSRNOG00000059510 | *LOC100910792* | 2.93 | 1.02 | 1.378029 |
| ENSRNOG00000042825 | *Cd300le* | 11.64 | 22.45 | -1.14236 |
| ENSRNOG00000046295 | *Ubald1* | 0 | 8.02 | -6.07447 |
| ENSRNOG00000002802 | *Cxcl1* | 7.84 | 1.81 | 1.938798 |
| ENSRNOG00000049893 | *LOC100910934* | 2.16 | 0.03 | 5.851758 |
| ENSRNOG00000003054 | *Cask* | 4.73 | 5.39 | -1.19461 |
| ENSRNOG00000002794 | *Selp* | 1.49 | 3.03 | -1.17668 |
| ENSRNOG00000004757 | *Tmem158* | 9.6 | 3.01 | 1.501778 |
| ENSRNOG00000049491 | *RT1-DMb* | 2.07 | 5.53 | -1.57963 |
| ENSRNOG00000054806 | *LOC108348323* | 0.22 | 1.51 | -3.07671 |
| ENSRNOG00000008091 | *Gins1* | 5.56 | 11.11 | -1.16769 |
| ENSRNOG00000022309 | *Frem1* | 1.62 | 0.7 | 1.07888 |
| ENSRNOG00000054524 | *Tnip3* | 1.66 | 0.56 | 1.419172 |
| ENSRNOG00000022298 | *Cxcl11* | 1.83 | 0.13 | 3.670204 |
| ENSRNOG00000050888 | *Cyp4f37* | 0 | 0.84 | -5.9366 |
| ENSRNOG00000051772 | *AABR07027272.1* | 4.68 | 1.67 | 1.335024 |
| ENSRNOG00000003537 | *Spta1* | 1.2 | 0.48 | 1.177419 |
| ENSRNOG00000007956 | *Styx* | 11.13 | 4.58 | 1.117084 |
| ENSRNOG00000034093 | *AABR07011951.1* | 1.83 | 4.01 | -1.33774 |
| ENSRNOG00000018509 | *Cx3cr1* | 1.09 | 3.79 | -1.95429 |
| ENSRNOG00000049504 | *AABR07009221.1* | 1.74 | 0.67 | 1.22584 |
| ENSRNOG00000009403 | *Dcaf17* | 4.5 | 1.65 | 1.29947 |
| ENSRNOG00000057851 | *LOC680254* | 9.28 | 16.71 | -1.02704 |
| ENSRNOG00000023473 | *Rnf122* | 7.06 | 11.16 | -1.02787 |
| ENSRNOG00000052395 | *AABR07071395.1* | 2.79 | 0.26 | 3.045714 |
| ENSRNOG00000019542 | *LOC100910979* | 4.72 | 1.52 | 1.47951 |
| ENSRNOG00000033564 | *Cfd* | 5.96 | 12.66 | -1.27055 |
| ENSRNOG00000061900 | *AABR07047771.1* | 0.36 | 0 | 5.862825 |
| ENSRNOG00000002609 | *Ero1b* | 3.74 | 1.74 | 1.02091 |
| ENSRNOG00000018092 | *Cd83* | 0.78 | 2.35 | -1.73451 |
| ENSRNOG00000048881 | *LOC680910* | 2.34 | 5.99 | -1.52429 |
| ENSRNOG00000042686 | *Trim34* | 3.47 | 1.34 | 1.227043 |
| ENSRNOG00000054751 | *Lmbrd2* | 3.2 | 1.43 | 1.020328 |
| ENSRNOG00000024272 | *Ino80d* | 1.86 | 0.61 | 1.460751 |
| ENSRNOG00000002568 | *Socs1* | 5.01 | 1.23 | 1.863875 |
| ENSRNOG00000030187 | *Mmp12* | 2.29 | 4.75 | -1.20635 |
| ENSRNOG00000019293 | *LOC100911840* | 5.65 | 10.96 | -1.12827 |
| ENSRNOG00000050396 | *rno-mir-3542-1* | 0 | 3.15 | -5.73774 |
| ENSRNOG00000001270 | *Hvcn1* | 3.83 | 7.89 | -1.20636 |
| ENSRNOG00000023969 | *Herc6* | 0.88 | 0.15 | 2.430377 |
| ENSRNOG00000046862 | *LOC108348137* | 5.21 | 2.26 | 1.056378 |
| ENSRNOG00000031834 | *Nkain4* | 2.12 | 6.45 | -1.75179 |
| ENSRNOG00000052725 | *RGD1566029* | 1.15 | 0.38 | 1.466045 |
| ENSRNOG00000004843 | *Pole3* | 6.35 | 21.68 | -2.05428 |
| ENSRNOG00000046120 | *RGD1561252* | 0 | 3.63 | -5.68656 |
| ENSRNOG00000004221 | *Lgr5* | 1.46 | 0.38 | 1.789875 |
| ENSRNOG00000016469 | *Fxyd2* | 6.31 | 15.88 | -1.61421 |
| ENSRNOG00000046745 | *LOC691918* | 10.26 | 4.21 | 1.520048 |
| ENSRNOG00000005809 | *Arhgdib* | 4.94 | 9.21 | -1.06493 |
| ENSRNOG00000008223 | *Cnr1* | 0.31 | 0.01 | 4.746153 |
| ENSRNOG00000004857 | *L2hgdh* | 2.73 | 1.13 | 1.12494 |
| ENSRNOG00000028015 | *Pf4* | 33.66 | 82.7 | -1.68468 |
| ENSRNOG00000019736 | *Nfs1* | 1.42 | 0 | 5.667472 |
| ENSRNOG00000003439 | *Il15* | 2.19 | 7.03 | -1.25685 |
| ENSRNOG00000036598 | *LOC685933* | 5.27 | 2.36 | 1.015241 |
| ENSRNOG00000010266 | *Cd180* | 0.7 | 1.82 | -1.53925 |
| ENSRNOG00000004720 | *Kcnj2* | 3.16 | 0.9 | 1.651435 |
| ENSRNOG00000009795 | *Nfib* | 3.01 | 0.98 | 1.392164 |
| ENSRNOG00000009919 | *Acod1* | 1.01 | 0.14 | 2.670204 |
| ENSRNOG00000050706 | *Col25a1* | 4.35 | 1.68 | 1.258707 |
| ENSRNOG00000016525 | *Susd3* | 0.13 | 1.54 | -3.76164 |
| ENSRNOG00000024889 | *Snip1* | 0.65 | 0 | 5.559204 |
| ENSRNOG00000006326 | *Atraid* | 0 | 1.74 | -5.51378 |
| ENSRNOG00000049949 | *Hapln4* | 0.78 | 0.14 | 2.302053 |
| ENSRNOG00000003384 | *Hs3st3b1* | 1.23 | 0.37 | 1.583631 |
| ENSRNOG00000059363 | *AC130862.3* | 2.5 | 0.73 | 1.631326 |
| ENSRNOG00000047005 | *Kcnk5* | 1.54 | 0.51 | 1.442604 |
| ENSRNOG00000059938 | *AC120712.2* | 0.93 | 0 | 5.505145 |
| ENSRNOG00000020173 | *Tie1* | 0.19 | 0.72 | -2.07982 |
| ENSRNOG00000059506 | *AC124926.2* | 0.28 | 0 | 5.48733 |
| ENSRNOG00000050582 | *LOC108348189* | 3.69 | 0 | 5.484006 |
| ENSRNOG00000048562 | *Polr2l* | 2.03 | 0 | 5.483341 |
| ENSRNOG00000020774 | *LOC103690114* | 2.72 | 1.11 | 1.143238 |
| ENSRNOG00000014465 | *Oca2* | 6.76 | 2.71 | 1.158902 |
| ENSRNOG00000021199 | *Fcgr1a* | 3.29 | 6.49 | -1.14332 |
| ENSRNOG00000004330 | *Chrdl1* | 1.92 | 0.81 | 1.095467 |
| ENSRNOG00000023407 | *Mrpl36* | 5.63 | 10.97 | -1.14406 |
| ENSRNOG00000007540 | *Msc* | 0 | 3.78 | -5.41372 |
| ENSRNOG00000010906 | *Ccl5* | 11.37 | 2.18 | 2.15005 |
| ENSRNOG00000026378 | *LOC100911204* | 0.28 | 0.02 | 3.699204 |
| ENSRNOG00000052977 | *LOC103690033* | 1.98 | 0.44 | 2.00693 |
| ENSRNOG00000020579 | *Col7a1* | 0.85 | 0.38 | 1.034032 |
| ENSRNOG00000059485 | *Cdkl3* | 1.35 | 0.78 | 1.966499 |
| ENSRNOG00000057798 | *AC102976.1* | 4.57 | 1.59 | 1.361427 |
| ENSRNOG00000014156 | *Fut7* | 0.11 | 0.91 | -3.17668 |
| ENSRNOG00000022166 | *Ammecr1* | 7.03 | 2.45 | 1.349494 |
| ENSRNOG00000047864 | *RT1-DMa* | 4.58 | 8.66 | -1.0363 |
| ENSRNOG00000039481 | *AABR07008300.1* | 15.08 | 6.48 | 1.016388 |
| ENSRNOG00000012302 | *Gucy1a3* | 0.48 | 1.06 | -1.30221 |
| ENSRNOG00000010997 | *Ednrb* | 1.72 | 0.42 | 1.899863 |
| ENSRNOG00000056701 | *Gtf2a2* | 0 | 7.6 | -5.26971 |
| ENSRNOG00000038955 | *MGC105567* | 1.33 | 0.27 | 2.161191 |
| ENSRNOG00000042478 | *Adam22* | 2.82 | 1.24 | 1.118962 |
| ENSRNOG00000049282 | *Oas2* | 0.62 | 0 | 5.293641 |
| ENSRNOG00000008861 | *AABR07047011.1* | 1.94 | 0.78 | 1.177048 |
| ENSRNOG00000009590 | *Stox2* | 0.32 | 0.02 | 4.373401 |
| ENSRNOG00000061442 | *AABR07068214.1* | 9.93 | 3.44 | 1.339046 |
| ENSRNOG00000005122 | *Uvssa* | 2.23 | 0.85 | 1.236614 |
| ENSRNOG00000002070 | *Mrpl1* | 1.43 | 0 | 5.253606 |
| ENSRNOG00000014975 | *Zfp483* | 1.3 | 0.28 | 2.045714 |
| ENSRNOG00000014660 | *Usp53* | 1.22 | 0.55 | 1.002645 |
| ENSRNOG00000042340 | *LOC679894* | 0.72 | 0.22 | 1.556675 |
| ENSRNOG00000052887 | *Dnajc6* | 0.72 | 0.23 | 1.556675 |
| ENSRNOG00000036964 | *Ralgapa2* | 0.73 | 0.24 | 1.438031 |
| ENSRNOG00000023214 | *Il20rb* | 1.47 | 0.07 | 4.293641 |
| ENSRNOG00000048060 | *RGD1563072* | 4.43 | 0.55 | 2.803741 |
| ENSRNOG00000037331 | *Cd33* | 1.45 | 3.39 | -1.41372 |
| ENSRNOG00000003526 | *Sytl4* | 3.27 | 1.1 | 1.127243 |
| ENSRNOG00000004810 | *Plcb1* | 0.54 | 0.1 | 2.282753 |
| ENSRNOG00000021384 | *Ankrd44* | 2.06 | 0.87 | 1.092007 |
| ENSRNOG00000000443 | *C4a* | 0.81 | 0.29 | 1.370582 |
| ENSRNOG00000060341 | *AABR07031399.1* | 1.25 | 0.39 | 1.53114 |
| ENSRNOG00000060878 | *AABR07066020.1* | 0.46 | 2.07 | -3.3944 |
| ENSRNOG00000008716 | *Nefh* | 1.67 | 0.72 | 1.076087 |
| ENSRNOG00000003189 | *Cited1* | 8.54 | 3.45 | 1.225037 |
| ENSRNOG00000015992 | *Ccl20* | 2.73 | 0.22 | 3.438031 |
| ENSRNOG00000009694 | *Bmp4* | 1.69 | 3.32 | -1.12421 |
| ENSRNOG00000013564 | *Dok3* | 0.81 | 2.25 | -1.62671 |
| ENSRNOG00000027451 | *Mettl25* | 2.95 | 1.27 | 1.070047 |
| ENSRNOG00000050854 | *Clcn2* | 0.74 | 1.48 | -1.29355 |
| ENSRNOG00000059400 | *AABR07070796.1* | 0 | 1.13 | -5.04768 |
| ENSRNOG00000009431 | *Tbc1d4* | 1.73 | 0.76 | 1.045714 |
| ENSRNOG00000002227 | *Kit* | 0.66 | 0.21 | 1.525706 |
| ENSRNOG00000046305 | *LOC103690163* | 1.16 | 2.21 | -1.07433 |
| ENSRNOG00000046314 | *AABR07044473.1* | 1.08 | 0.04 | 4.449152 |
| ENSRNOG00000047509 | *AABR07047844.1* | 1.08 | 0.04 | 4.449152 |
| ENSRNOG00000048192 | *AABR07070238.3* | 1.08 | 0.04 | 4.449152 |
| ENSRNOG00000050867 | *AABR07050530.1* | 1.08 | 0.04 | 4.449152 |
| ENSRNOG00000055972 | *AC128394.1* | 1.08 | 0.04 | 4.449152 |
| ENSRNOG00000036880 | *Arl5c* | 1.81 | 0.19 | 2.68957 |
| ENSRNOG00000023614 | *Hsh2d* | 0.48 | 0 | 5.045714 |
| ENSRNOG00000016289 | *Bmpr1b* | 3.17 | 1.29 | 1.137636 |
| ENSRNOG00000001752 | *Nrros* | 0.88 | 1.87 | -1.23722 |
| ENSRNOG00000057463 | *AABR07008293.3* | 1.22 | 0 | 5.037575 |
| ENSRNOG00000048982 | *LOC100912282* | 0.36 | 0 | 5.036668 |
| ENSRNOG00000028592 | *AABR07035074.1* | 2.15 | 0.92 | 1.080013 |
| ENSRNOG00000011063 | *Dennd1b* | 3.62 | 1.3 | 1.097552 |
| ENSRNOG00000045973 | *LOC102553861* | 2.91 | 5.87 | -1.17101 |
| ENSRNOG00000030131 | *Zscan30* | 1.82 | 0.36 | 2.192555 |
| ENSRNOG00000061902 | *Wbp1* | 4.96 | 1.7 | 1.596913 |
| ENSRNOG00000049388 | *Prr32* | 1.33 | 0.07 | 4.133176 |
| ENSRNOG00000060289 | *7SK* | 23.81 | 0 | 5.005484 |
| ENSRNOG00000007112 | *Pcsk1n* | 0 | 1.79 | -4.96148 |
| ENSRNOG00000045998 | *Sema6b* | 0.05 | 0.35 | -2.89289 |
| ENSRNOG00000040108 | *Cd36* | 0.14 | 0.75 | -2.42781 |
| ENSRNOG00000014867 | *Synpo2* | 0.98 | 0.35 | 1.332595 |
| ENSRNOG00000006893 | *Ppm1k* | 3.73 | 1.5 | 1.01534 |
| ENSRNOG00000005472 | *Sp4* | 1.59 | 0.67 | 1.100161 |
| ENSRNOG00000057009 | *LOC103693189* | 14.6 | 4.36 | 1.501029 |
| ENSRNOG00000013674 | *Megf10* | 0.72 | 0.31 | 1.062787 |
| ENSRNOG00000057443 | *LOC497963* | 0.29 | 0 | 4.952604 |
| ENSRNOG00000058862 | *AABR07051308.1* | 0.39 | 0 | 4.941985 |
| ENSRNOG00000005046 | *Tspan13* | 2.87 | 1.19 | 1.123716 |
| ENSRNOG00000026944 | *Gpr150* | 0.37 | 1.15 | -1.78026 |
| ENSRNOG00000015718 | *RGD1307461* | 0.41 | 0.02 | 4.045714 |
| ENSRNOG00000016971 | *Zfp612* | 1.87 | 0.63 | 1.424225 |
| ENSRNOG00000019082 | *Sbk1* | 0.56 | 0.13 | 1.917498 |
| ENSRNOG00000014443 | *Pde5a* | 1.54 | 0.65 | 1.103047 |
| ENSRNOG00000053095 | *Rn60_20_0051.7* | 0 | 2.23 | -4.85925 |
| ENSRNOG00000006814 | *Klrg2* | 2.37 | 0.56 | 1.920183 |
| ENSRNOG00000054080 | *Cgnl1* | 0.39 | 0.11 | 1.667202 |
| ENSRNOG00000029938 | *Pik3c2b* | 0.11 | 0.32 | -1.64616 |
| ENSRNOG00000055056 | *AABR07062390.2* | 5.62 | 1.83 | 1.43354 |
| ENSRNOG00000045683 | *LOC102553715* | 0.73 | 0 | 4.859238 |
| ENSRNOG00000048053 | *Rn50_10_0701.2* | 2.08 | 0.16 | 3.215639 |
| ENSRNOG00000011947 | *Tifab* | 0.36 | 1.44 | -2.17668 |
| ENSRNOG00000022358 | *Rn60_16_0588.3* | 2.05 | 0.55 | 1.729504 |
| ENSRNOG00000030486 | *Prdm6* | 0.58 | 1.55 | -1.5732 |
| ENSRNOG00000009465 | *Sfrp2* | 0 | 0.37 | -4.76164 |
| ENSRNOG00000010608 | *Cep162* | 0.85 | 0.34 | 1.133176 |
| ENSRNOG00000023337 | *Sema3a* | 1.3 | 0.53 | 1.161191 |
| ENSRNOG00000010283 | *Cd28* | 1.41 | 0.22 | 2.505145 |
| ENSRNOG00000052864 | *LOC108348326* | 0 | 0.95 | -4.7325 |
| ENSRNOG00000005053 | *Egln3* | 0.59 | 0.11 | 2.308748 |
| ENSRNOG00000027430 | *Ikzf2* | 1.05 | 0.23 | 2.161191 |
| ENSRNOG00000011718 | *C1rl* | 1.47 | 0.51 | 1.382748 |
| ENSRNOG00000048118 | *LOC100910438* | 0 | 0.45 | -4.70917 |
| ENSRNOG00000021752 | *Foxq1* | 2.38 | 0.85 | 1.325821 |
| ENSRNOG00000010468 | *Elovl6* | 0 | 0.9 | -4.70704 |
| ENSRNOG00000028768 | *Gbp4* | 0.45 | 0.05 | 3.133176 |
| ENSRNOG00000058538 | *AABR07002001.1* | 1.47 | 3.23 | -1.30443 |
| ENSRNOG00000050414 | *AABR07006333.1* | 3.78 | 0.49 | 2.732027 |
| ENSRNOG00000010880 | *Gpr27* | 2.47 | 0.65 | 1.76818 |
| ENSRNOG00000051563 | *Giot1* | 1.23 | 0.33 | 1.76818 |
| ENSRNOG00000015480 | *Prkcz* | 1.14 | 0.46 | 1.169096 |
| ENSRNOG00000004084 | *Fam84a* | 1.09 | 0.41 | 1.286722 |
| ENSRNOG00000060718 | *AABR07064253.1* | 0.16 | 0 | 4.71247 |
| ENSRNOG00000040303 | *LOC103692531* | 29.71 | 22.95 | -1.61725 |
| ENSRNOG00000048650 | *LOC103690175* | 1.44 | 0 | 4.706779 |
| ENSRNOG00000028444 | *AABR07006258.1* | 0 | 3.35 | -4.66358 |
| ENSRNOG00000061365 | *AABR07007055.1* | 0.67 | 0.04 | 3.853068 |
| ENSRNOG00000008101 | *Tmem251* | 22.27 | 8.44 | 1.141929 |
| ENSRNOG00000050910 | *LOC688583* | 4.03 | 1.83 | 1.427353 |
| ENSRNOG00000006231 | *Ptpro* | 0.3 | 0.62 | -1.19307 |
| ENSRNOG00000028016 | *Ncf2* | 2.57 | 4.82 | -1.01162 |
| ENSRNOG00000046699 | *Slpi* | 2.84 | 0.58 | 2.104607 |
| ENSRNOG00000053168 | *LOC102553088* | 1.1 | 0.31 | 1.673745 |
| ENSRNOG00000050703 | *Fam115c* | 1.36 | 0.55 | 1.16782 |
| ENSRNOG00000038087 | *AC110846.1* | 4.86 | 0 | 4.6663 |
| ENSRNOG00000039379 | *RGD1562963* | 0.1 | 0.72 | -3.04175 |
| ENSRNOG00000026324 | *Stard6* | 0.32 | 1.43 | -2.21732 |
| ENSRNOG00000001006 | *Nptx2* | 1.11 | 0.37 | 1.438031 |
| ENSRNOG00000011193 | *Hmgcll1* | 0.78 | 0.27 | 1.367642 |
| ENSRNOG00000007545 | *Angptl4* | 2.11 | 0.82 | 1.215639 |
| ENSRNOG00000009211 | *C3ar1* | 1.01 | 1.98 | -1.12421 |
| ENSRNOG00000016041 | *Zc3h12d* | 0.02 | 0.22 | -3.76164 |
| ENSRNOG00000047363 | *Kcnk13* | 0.04 | 0.44 | -3.76164 |
| ENSRNOG00000053620 | *AABR07051069.1* | 0.02 | 0.43 | -4.73623 |
| ENSRNOG00000028649 | *Tox3* | 0.26 | 0 | 4.630676 |
| ENSRNOG00000029591 | *Pkdrej* | 0.16 | 0.02 | 3.045714 |
| ENSRNOG00000059362 | *Has3* | 0.15 | 0.02 | 3.045714 |
| ENSRNOG00000046981 | *Gp1bb* | 0.97 | 2.78 | -1.68981 |
| ENSRNOG00000049178 | *AABR07028414.1* | 0.15 | 0 | 4.618603 |
| ENSRNOG00000001287 | *Gper1* | 1.72 | 3.36 | -1.13014 |
| ENSRNOG00000010018 | *Clec4a3* | 3.7 | 6.96 | -1.10113 |
| ENSRNOG00000002462 | *LOC108348055* | 3.18 | 6.09 | -1.12042 |
| ENSRNOG00000007076 | *LOC691422* | 0.3 | 0.95 | -1.81227 |
| ENSRNOG00000004409 | *Sash3* | 0.43 | 1.01 | -1.38055 |
| ENSRNOG00000053891 | *Phf11b* | 1.41 | 0.28 | 2.183217 |
| ENSRNOG00000022599 | *Ints1* | 1.54 | 0.63 | 1.153011 |
| ENSRNOG00000023493 | *Creb3l4* | 0 | 0.27 | -4.52232 |
| ENSRNOG00000000245 | *Slc16a6* | 1.1 | 0.31 | 1.677982 |
| ENSRNOG00000017092 | *Zfp169* | 9.11 | 0.73 | 1.04436 |
| ENSRNOG00000020699 | *Cd37* | 1.11 | 2.46 | -1.30792 |
| ENSRNOG00000061317 | *AABR07011733.1* | 1.05 | 0.13 | 2.827297 |
| ENSRNOG00000051253 | *LOC102553828* | 2.2 | 0 | 4.538848 |
| ENSRNOG00000000414 | *Cep85l* | 1.25 | 0.48 | 1.194577 |
| ENSRNOG00000059095 | *SNORD101* | 4.12 | 1.43 | 1.350568 |
| ENSRNOG00000014030 | *Synm* | 0.63 | 1.15 | 1.023687 |
| ENSRNOG00000054458 | *Kcnmb4* | 0.76 | 2.04 | -1.58232 |
| ENSRNOG00000020151 | *Cdh1* | 0.02 | 0.17 | -3.65473 |
| ENSRNOG00000050600 | *Zfp872* | 0.85 | 0.1 | 2.952604 |
| ENSRNOG00000047294 | *AABR07043115.1* | 0.02 | 0.16 | -3.4031 |
| ENSRNOG00000014265 | *Tnfrsf19* | 1.67 | 0.73 | 1.200992 |
| ENSRNOG00000004947 | *Zfp871* | 1.45 | 0.53 | 1.302053 |
| ENSRNOG00000004812 | *Sema6d* | 0.23 | 0.54 | -1.20583 |
| ENSRNOG00000061182 | *Gabre* | 0.61 | 0.16 | 1.789336 |
| ENSRNOG00000000855 | *Lst1* | 3.8 | 10.31 | -1.76164 |
| ENSRNOG00000010344 | *B4galnt3* | 0.09 | 0.41 | -2.27621 |
| ENSRNOG00000020431 | *Matk* | 0.19 | 0.81 | -2.27621 |
| ENSRNOG00000002771 | *Ereg* | 0.79 | 0.18 | 1.984313 |
| ENSRNOG00000020061 | *Tmem198* | 0.96 | 2.01 | -1.21732 |
| ENSRNOG00000010350 | *Rcan2* | 0.77 | 0.27 | 1.38675 |
| ENSRNOG00000014685 | *LOC108348167* | 0 | 0.25 | -4.41372 |
| ENSRNOG00000037366 | *Zfp939* | 0 | 0.45 | -4.41372 |
| ENSRNOG00000012369 | *Fam174b* | 0.66 | 0.05 | 3.630676 |

**Supplemental Table 5. The Gene Ontology Analysis based on different expressed genes between HO-1/BMMSCs and GFP/BMMSCs.**

| Number | GO ID | Description | Ratio in study | Ratio in pop | P-value corrected |
| --- | --- | --- | --- | --- | --- |
| 21 | GO:0051607 | defense response to virus | 21/557 | 117/21716 | 1.95E-07 |
| 40 | GO:0002252 | immune effector process | 40/557 | 420/21716 | 1.95E-07 |
| 56 | GO:0043207 | response to external biotic stimulus | 56/557 | 749/21716 | 1.95E-07 |
| 26 | GO:0009615 | response to virus | 26/557 | 202/21716 | 1.95E-07 |
| 64 | GO:0006955 | immune response | 64/557 | 885/21716 | 1.95E-07 |
| 73 | GO:0002682 | regulation of immune system process | 73/557 | 1219/21716 | 1.95E-07 |
| 57 | GO:0009607 | response to biotic stimulus | 57/557 | 783/21716 | 1.95E-07 |
| 75 | GO:0006952 | defense response | 75/557 | 898/21716 | 1.95E-07 |
| 93 | GO:0002376 | immune system process | 93/557 | 1498/21716 | 1.95E-07 |
| 54 | GO:0002684 | positive regulation of immune system process | 54/557 | 814/21716 | 1.95E-07 |
| 15 | GO:0034341 | response to interferon-gamma | 15/557 | 72/21716 | 3E-07 |
| 74 | GO:0009605 | response to external stimulus | 74/557 | 1346/21716 | 4.9E-07 |
| 33 | GO:0034097 | response to cytokine | 33/557 | 377/21716 | 7.83E-07 |
| 18 | GO:0030595 | leukocyte chemotaxis | 18/557 | 121/21716 | 1.51E-06 |
| 10 | GO:0035456 | response to interferon-beta | 10/557 | 30/21716 | 1.61E-06 |
| 39 | GO:0051707 | response to other organism | 39/557 | 524/21716 | 2.25E-06 |
| 30 | GO:0006954 | inflammatory response | 30/557 | 335/21716 | 2.32E-06 |
| 33 | GO:0098542 | defense response to other organism | 33/557 | 404/21716 | 3.71E-06 |
| 13 | GO:0070098 | chemokine-mediated signaling pathway | 13/557 | 63/21716 | 3.82E-06 |
| 21 | GO:0050900 | leukocyte migration | 21/557 | 183/21716 | 6.67E-06 |
| 9 | GO:0035458 | cellular response to interferon-beta | 9/557 | 27/21716 | 8.32E-06 |
| 20 | GO:0060326 | cell chemotaxis | 20/557 | 171/21716 | 1.02E-05 |
| 29 | GO:0001819 | positive regulation of cytokine production | 29/557 | 342/21716 | 1.15E-05 |
| 25 | GO:0071345 | cellular response to cytokine stimulus | 25/557 | 263/21716 | 1.15E-05 |
| 37 | GO:0001817 | regulation of cytokine production | 37/557 | 518/21716 | 1.25E-05 |
| 87 | GO:0048584 | positive regulation of response to stimulus | 87/557 | 1855/21716 | 1.75E-05 |
| 30 | GO:0045087 | innate immune response | 30/557 | 378/21716 | 2.58E-05 |
| 24 | GO:0006935 | chemotaxis | 24/557 | 259/21716 | 3.09E-05 |
| 24 | GO:0042330 | taxis | 24/557 | 260/21716 | 3.23E-05 |
| 44 | GO:0050776 | regulation of immune response | 44/557 | 707/21716 | 3.23E-05 |
| 88 | GO:0010033 | response to organic substance | 88/557 | 1933/21716 | 4.39E-05 |
| 25 | GO:0031349 | positive regulation of defense response | 25/557 | 286/21716 | 4.71E-05 |
| 26 | GO:0032103 | positive regulation of response to external stimulus | 26/557 | 307/21716 | 4.82E-05 |
| 7 | GO:1901623 | regulation of lymphocyte chemotaxis | 7/557 | 18/21716 | 7.17E-05 |
| 108 | GO:0006950 | response to stress | 108/557 | 2570/21716 | 7.17E-05 |
| 5 | GO:2000501 | regulation of natural killer cell chemotaxis | 5/557 | 7/21716 | 8.71E-05 |
| 68 | GO:0051240 | positive regulation of multicellular organismal process | 68/557 | 1385/21716 | 9.46E-05 |
| 14 | GO:0002705 | positive regulation of leukocyte mediated immunity | 14/557 | 101/21716 | 0.000115 |
| 25 | GO:0019221 | cytokine-mediated signaling pathway | 25/557 | 306/21716 | 0.000146 |
| 38 | GO:0031347 | regulation of defense response | 38/557 | 610/21716 | 0.000214 |
| 13 | GO:0097529 | myeloid leukocyte migration | 13/557 | 92/21716 | 0.000217 |
| 12 | GO:0002708 | positive regulation of lymphocyte mediated immunity | 12/557 | 80/21716 | 0.000285 |
| 10 | GO:0071346 | cellular response to interferon-gamma | 10/557 | 56/21716 | 0.000436 |
| 29 | GO:0001775 | cell activation | 29/557 | 418/21716 | 0.000436 |
| 14 | GO:0002687 | positive regulation of leukocyte migration | 14/557 | 116/21716 | 0.000498 |
| 13 | GO:0002688 | regulation of leukocyte chemotaxis | 13/557 | 100/21716 | 0.000498 |
| 43 | GO:0051270 | regulation of cellular component movement | 43/557 | 764/21716 | 0.000512 |
| 16 | GO:0002685 | regulation of leukocyte migration | 16/557 | 151/21716 | 0.000512 |
| 8 | GO:0048247 | lymphocyte chemotaxis | 8/557 | 34/21716 | 0.000512 |

**Supplemental Table 6. The Kyoto Encyclopedia of Genes and Genomes analysis based on different expressed genes between HO-1/BMMSCs and GFP/BMMSCs.**

| Number | Pathway id | Description | Ratio in study | Ratio in pop | P-value corrected |
| --- | --- | --- | --- | --- | --- |
| 18 | map04621 | NOD-like receptor signaling pathway | 18/296 | 199/16940 | 0 |
| 19 | map05164 | Influenza A | 19/296 | 224/16940 | 0 |
| 20 | map05168 | Herpes simplex infection | 20/296 | 263/16940 | 0 |
| 16 | map04062 | Chemokine signaling pathway | 16/296 | 194/16940 | 0.0001 |
| 18 | map04060 | Cytokine-cytokine receptor interaction | 18/296 | 270/16940 | 0.0003 |
| 8 | map04622 | RIG-I-like receptor signaling pathway | 8/296 | 71/16940 | 0.0069 |
| 11 | map04380 | Osteoclast differentiation | 11/296 | 140/16940 | 0.0075 |
| 15 | map05162 | Measles | 15/296 | 264/16940 | 0.014 |
| 11 | map05160 | Hepatitis C | 11/296 | 155/16940 | 0.0189 |
| 9 | map04668 | TNF signaling pathway | 9/296 | 116/16940 | 0.042 |
| 6 | map04623 | Cytosolic DNA-sensing pathway | 6/296 | 64/16940 | 0.1812 |
| 9 | map05150 | Staphylococcus aureus infection | 9/296 | 165/16940 | 0.5211 |
| 7 | map04657 | IL-17 signaling pathway | 7/296 | 108/16940 | 0.595 |
| 6 | map04918 | Thyroid hormone synthesis | 6/296 | 82/16940 | 0.6424 |
| 1 | map04010 | MAPK signaling pathway | 1/296 | 297/16940 | 0.9949 |
| 9 | map04022 | cGMP-PKG signaling pathway | 9/296 | 192/16940 | 1 |
| 6 | map04610 | Complement and coagulation cascades | 6/296 | 97/16940 | 1 |
| 7 | map04919 | Thyroid hormone signaling pathway | 7/296 | 138/16940 | 1 |
| 1 | map00660 | C5-Branched dibasic acid metabolism | 1/296 | 1/16940 | 1 |
| 7 | map04611 | Platelet activation | 7/296 | 158/16940 | 1 |
| 5 | map04911 | Insulin secretion | 5/296 | 96/16940 | 1 |
| 7 | map04640 | Hematopoietic cell lineage | 7/296 | 166/16940 | 1 |
| 6 | map04915 | Estrogen signaling pathway | 6/296 | 137/16940 | 1 |
| 4 | map04978 | Mineral absorption | 4/296 | 75/16940 | 1 |
| 6 | map05142 | Chagas disease (American trypanosomiasis) | 6/296 | 150/16940 | 1 |

**Supplemental Table 7. Different abundant proteins between IEC-6s treated with BM-exo and TNF-α-exo.**

| Protein accession | Protein name | Relative expression (IEC-6s treated with BM-exo) | Relative expression (IEC-6s treated with TNF-α-exo) | Fold Change (M/T) |
| --- | --- | --- | --- | --- |
| P05065 | ALDOA | 0.703 | 1.603 | 0.439 |
| P23358 | RPL12 | 0.308 | 1.378 | 0.224 |
| Q6PDV8 | LOC100360057 | 0.68 | 1.366 | 0.498 |
| A0A0G2JUQ5 | PICALM | 0.354 | 1.646 | 0.215 |
| P05982 | NQO1 | 1.399 | 0.508 | 2.754 |
| F1LUM5 | TUBAL3 | 1.391 | 0.238 | 5.845 |
| Q6B345 | S100A11 | 2.619 | 0.285 | 9.189 |
| P62755 | RPS6 | 0.387 | 2.059 | 0.188 |
| B5DEN5 | EEF1B2 | 0.343 | 0.887 | 0.387 |
| A0A096MJB3 | WDR82 | 1.474 | 0.483 | 3.052 |
| Q9R1N3 | SLC4A7 | 1.945 | 0.675 | 2.881 |
| A0A0G2JXM6 | RBM42 | 0.671 | 1.432 | 0.469 |
| P61972 | NUTF2 | 0.624 | 1.461 | 0.427 |
| D3ZPL1 | CPSF6 | 1.414 | 0.113 | 12.513 |
| M0R402 | TMX3 | 0.356 | 1.894 | 0.188 |
| A0A140TAA8 | NDC1 | 1.185 | 0.564 | 2.101 |
| A0A096MKH2 | VTA1 | 0.564 | 1.344 | 0.42 |
| Q3MHS2 | ZNF830 | 0.718 | 1.481 | 0.485 |
| B0BNM1 | NAXE | 0.68 | 1.432 | 0.475 |
| A0A0G2JUV4 | MRPL1 | 1.247 | 0.309 | 4.036 |
| Q6P503 | ATP6V1D | 0.318 | 1.245 | 0.255 |
| D3ZA85 | NFU1 | 1.055 | 0.502 | 2.102 |
| F8WG67 | ACOT7 | 0.354 | 1.243 | 0.285 |
| G3V734 | DECR1 | 0.215 | 0.962 | 0.223 |
| P41777 | NOLC1 | 1.382 | 0.618 | 2.236 |
| Q5M849 | IFI35 | 0.614 | 1.429 | 0.43 |
| A0A0G2K508 | RRAS2 | 1.306 | 0.49 | 2.665 |
| F1M978 | IMPA1 | 0.72 | 1.544 | 0.466 |
| G3V7R1 | NUP50 | 1.309 | 0.62 | 2.111 |
| B5DFG8 | PYGO2 | 0.529 | 1.552 | 0.341 |
| Q5XJW2 | GADD45GIP1 | 1.28 | 0.474 | 2.7 |
| Q6AYD9 | NUDT19 | 0.417 | 2.106 | 0.198 |
| Q66H15 | RMDN3 | 0.616 | 1.442 | 0.427 |
| D3ZT98 | BOLA3 | 1.209 | 0.264 | 4.58 |
| A0A0G2JY75 | GTF3C2 | 1.064 | 0.508 | 2.094 |
| P11980 | PKM | 0.335 | 0.801 | 0.418 |
| Q4QQU6 | SMNDC1 | 0.173 | 1.447 | 0.12 |
| D3ZUD8 | TM9SF3 | 1.032 | 0.414 | 2.493 |
| P13233 | CNP | 1.157 | 0.563 | 2.055 |
| D3ZU64 | VPS37B | 0.488 | 1.493 | 0.327 |
| D3ZPD0 | CKAP2 | 1.091 | 0.506 | 2.156 |
| G3V8M5 | PPP4C | 0.461 | 0.924 | 0.499 |
| P11240 | COX5A | 1.498 | 0.624 | 2.401 |
| F1LMQ3 | PSMD8 | 0.656 | 1.344 | 0.488 |
| Q6IRE4 | TSG101 | 1.378 | 0.453 | 3.042 |
| Q7TSU1 | ARFGEF2 | 1.103 | 0.529 | 2.085 |
| Q5RJN0 | NDUFS7 | 0.764 | 1.533 | 0.498 |
| F1M771 | RYBP | 1.16 | 0.424 | 2.736 |
| Q810T5 | KAT7 | 1.21 | 0.588 | 2.058 |
| D3ZUT9 | INTS3 | 0.611 | 0.196 | 3.117 |
| M0R7E6 | --- | 0.069 | 1.505 | 0.046 |
| D3ZFL3 | MAP3K6 | 0.388 | 2.029 | 0.191 |
| A0A0G2KA57 | RALGAPB | 0.705 | 1.427 | 0.494 |
| G3V6R5 | SUOX | 0.462 | 1.003 | 0.461 |
| D3ZCL3 | SNRPC | 0.541 | 1.178 | 0.459 |
| P81799 | NAGK | 1.296 | 0.61 | 2.125 |
| A0A0G2K7P2 | PHF14 | 1.273 | 0.536 | 2.375 |
| Q4QRB4 | TUBB3 | 1.127 | 0.54 | 2.087 |
| Q8CFC1 | RIOX2 | 0.664 | 1.403 | 0.473 |
| B0BN83 | ARMC1 | 1.231 | 0.474 | 2.597 |
| D4A2Y9 | PEX13 | 0.238 | 0.797 | 0.299 |
| Q4V7D1 | SSR1 | 1.85 | 0.371 | 4.987 |
| A0A0G2JUK2 | LSM14A | 0.294 | 0.825 | 0.356 |
| Q9WVR8 | MEN1 | 0.57 | 1.43 | 0.399 |
| A0A096MJ98 | --- | 0.504 | 2.081 | 0.242 |
| A0A0G2K588 | LTBP4 | 0.89 | 0.435 | 2.046 |
| D4A563 | PEAK1 | 1.284 | 0.307 | 4.182 |
| P97546 | NPTN | 0.455 | 1.001 | 0.455 |
| F1LPG2 | DOCK8 | 0.588 | 1.257 | 0.468 |
| Q5U4E8 | PRMT7 | 1.311 | 0.603 | 2.174 |
| D3ZDZ7 | FUK | 1.575 | 0.77 | 2.045 |
| A0A0G2JYN0 | DCAF8 | 0.705 | 1.553 | 0.454 |
| Q923W4 | HDGFL3 | 0.226 | 1.386 | 0.163 |
| A0A0G2K9M4 | WDFY3 | 1.393 | 0.512 | 2.721 |
| B2RYQ2 | PTPA | 1.249 | 0.412 | 3.032 |
| G3V7S5 | SENP3 | 1.396 | 0.584 | 2.39 |
| Q496Z9 | TRMT1L | 1.363 | 0.625 | 2.181 |
| D3ZGJ0 | DHX32 | 1.752 | 0.798 | 2.195 |
| A0A096MJ42 | DLG3 | 0.335 | 0.809 | 0.414 |
| Q5M7U2 | ORC5 | 0.759 | 1.608 | 0.472 |
| Q5FWY5 | AIP | 1.636 | 0.745 | 2.196 |
| Q6AZ50 | ATG3 | 0.533 | 1.713 | 0.311 |
| Q497A9 | EIF4EBP2 | 0.342 | 1.932 | 0.177 |
| Q2MCP5 | WDR45B | 1.353 | 0.665 | 2.035 |
| Q8K1P9 | FADS3 | 0.287 | 0.818 | 0.351 |
| D4A465 | LAMTOR2 | 0.512 | 1.519 | 0.337 |
| D4ADB4 | CGGBP1 | 1.343 | 0.614 | 2.187 |
| Q5RK19 | SNF8 | 1.256 | 0.545 | 2.305 |
| F1LXU0 | MED13 | 0.682 | 1.401 | 0.487 |
| A0A0G2JWL3 | NF1 | 1.219 | 0.435 | 2.802 |
| D4ABP9 | FBXO3 | 0.53 | 1.896 | 0.28 |
| A0A0G2K4Y6 | HAUS6 | 0.505 | 1.041 | 0.485 |
| B2GV73 | ARPC3 | 1.544 | 0.571 | 2.704 |
| A0A0G2JX67 | PREPL | 1.626 | 0.518 | 3.139 |
| D4A986 | MBD2 | 0.48 | 1.005 | 0.478 |
| D3ZH92 | KLHL26 | 1.337 | 0.246 | 5.435 |
| F1M7K9 | --- | 1.342 | 0.39 | 3.441 |
| A1L108 | ARPC5L | 0.22 | 1.544 | 0.142 |
| P0C0A1 | VPS25 | 0.606 | 1.394 | 0.435 |
| G3V6R7 | OXSM | 0.562 | 1.29 | 0.436 |
| Q5BKC6 | HSPBAP1 | 0.331 | 2.148 | 0.154 |
| Q5I0J8 | PHF11L | 1.568 | 0.485 | 3.233 |
| D4A9F1 | TANGO6 | 1.469 | 0.627 | 2.343 |
| Q66H99 | NOL10 | 1.583 | 0.55 | 2.878 |
| Q3LRZ1 | CRTC2 | 0.643 | 1.341 | 0.479 |
| P00406 | MTCO2 | 0.437 | 1.798 | 0.243 |
| P56571 | --- | 0.637 | 1.413 | 0.451 |
| G3V7F5 | NR2C2 | 0.551 | 1.108 | 0.497 |
| A0A0G2K351 | UNC119B | 1.192 | 0.419 | 2.845 |
| Q3MIF0 | RPE | 1.791 | 0.886 | 2.021 |
| D4AAI2 | ARHGAP45 | 1.91 | 0.541 | 3.53 |
| Q5U1Z9 | MTX2 | 0.271 | 1.729 | 0.157 |
| D3ZYR1 | FCHO2 | 0.402 | 1.598 | 0.252 |
| F1M9C8 | KIF1C | 1.512 | 0.422 | 3.583 |
| M0R750 | SRCAP | 0.705 | 1.598 | 0.441 |
| D4A1G8 | CEP170B | 1.859 | 0.617 | 3.013 |
| D4A3T2 | GATAD1 | 1.234 | 0.585 | 2.109 |
| M0RA39 | MXRA7 | 1.352 | 0.191 | 7.079 |
| D3ZQJ3 | GIGYF1 | 1.615 | 0.446 | 3.621 |
| Q9ESM0 | IP6K1 | 1.186 | 0.455 | 2.607 |
| P52020 | SQLE | 1.553 | 0.447 | 3.474 |
| A0A0G2K590 | SPAST | 0.665 | 1.583 | 0.42 |
| B2RZ68 | DCAF7 | 0.486 | 1.42 | 0.342 |
| Q5XFW4 | MRPL13 | 1.775 | 0.545 | 3.257 |
| D3ZH66 | TAF5 | 0.531 | 1.402 | 0.379 |
| B5DF11 | ZFAND5 | 0.333 | 1.341 | 0.248 |
| A0A0G2K4R7 | RBMS1 | 0.634 | 1.669 | 0.38 |
| B5DFI8 | UBE2S | 1.801 | 0.528 | 3.411 |
| G3V9Q4 | STK38 | 1.501 | 0.499 | 3.008 |
| D4AAM1 | RGD1597339 | 1.16 | 0.524 | 2.214 |
| P62255 | UBE2G1 | 0.636 | 1.364 | 0.466 |
| F1M2K4 | LATS1 | 1.37 | 0.63 | 2.175 |
| G3V8H7 | OLFML3 | 1.127 | 0.502 | 2.245 |
| Q5XIC1 | GMPPA | 0.744 | 1.491 | 0.499 |
| A0A0H2UHR9 | STN1 | 0.485 | 1.354 | 0.358 |
| A0A0G2K038 | REXO2 | 1.286 | 0.413 | 3.114 |
| Q5EB92 | WDR70 | 0.601 | 1.248 | 0.482 |
| Q561Q8 | MED4 | 0.556 | 1.444 | 0.385 |
| A0A0G2K1A9 | NOL11 | 1.384 | 0.459 | 3.015 |
| A0A0G2K2L0 | MLH1 | 1.317 | 0.607 | 2.17 |
| M0RBJ0 | GNG2 | 1.35 | 0.532 | 2.538 |
| G3V728 | NIPSNAP1 | 1.75 | 0.86 | 2.035 |
| G3V7N6 | MBOAT7L1 | 1.401 | 0.599 | 2.339 |
| Q5M9I5 | UQCRH | 0.904 | 0.402 | 2.249 |
| F1LU48 | ERGIC1 | 1.179 | 0.198 | 5.955 |
| Q3B7D1 | UBE2Z | 0.586 | 1.414 | 0.414 |
| D3ZHF8 | GUF1 | 1.153 | 0.483 | 2.387 |
| Q2IBC5 | CAV2 | 1.367 | 0.633 | 2.16 |
| D4AEL8 | CEP131 | 1.456 | 0.374 | 3.893 |
| Q66H33 | --- | 0.588 | 1.412 | 0.416 |
| A0A0G2K2J0 | SHCBP1 | 1.389 | 0.611 | 2.273 |
| B2RYC8 | IRAK3 | 1.48 | 0.52 | 2.846 |
| Q6P777 | MVB12A | 1.096 | 0.523 | 2.096 |
| F1LWT0 | SIMC1 | 0.692 | 1.708 | 0.405 |
| F2Z3S3 | ST6GAL1 | 1.822 | 0.433 | 4.208 |
| Q9QX19 | CIT | 1.482 | 0.485 | 3.056 |
| Q3ZAU5 | DDHD1 | 1.448 | 0.573 | 2.527 |
| F1M4J0 | RICTOR | 1.566 | 0.774 | 2.023 |
| B5DFE0 | MPP6 | 0.265 | 1.085 | 0.244 |
| A0A0G2JVD0 | RBM28 | 1.648 | 0.303 | 5.439 |
| A0A0G2K0T2 | CD63 | 0.507 | 1.814 | 0.279 |
| G3V7B6 | PTPMT1 | 1.927 | 0.5 | 3.854 |
| D4A7K6 | MED10 | 0.446 | 1.554 | 0.287 |
| D3ZWQ8 | ARHGEF6 | 0.54 | 1.46 | 0.37 |
| D3Z9G0 | TRAF3 | 0.147 | 2.705 | 0.054 |
| B2RYB1 | TXNDC11 | 0.667 | 1.339 | 0.498 |
| F7DLY1 | EPS8L2 | 1.82 | 0.181 | 10.055 |
| A0A0G2JSR9 | SDF4 | 1.175 | 0.543 | 2.164 |
| F1M8K0 | DAG1 | 1.272 | 0.504 | 2.524 |
| D4A333 | TTC21B | 0.526 | 1.474 | 0.357 |
| Q5BJQ2 | MINDY1 | 0.651 | 1.349 | 0.483 |
| D4A3I5 | FAM160B1 | 0.441 | 1.559 | 0.283 |
| B2RZ99 | CKS1B | 0.578 | 1.422 | 0.406 |
| D3ZM59 | USP45 | 0.491 | 1.281 | 0.383 |
| B0BNM0 | TMEM222 | 1.279 | 0.261 | 4.9 |
| D3ZBZ9 | TTC33 | 1.604 | 0.495 | 3.24 |
| A0A096MJX5 | RGD1305178 | 1.166 | 0.496 | 2.351 |
| B1H264 | TBC1D17 | 1.268 | 0.416 | 3.048 |
| B2RZ96 | UBE2R2 | 1.542 | 0.458 | 3.367 |
| A0A0G2K393 | PLEK | 0.536 | 1.464 | 0.366 |
| D3ZMY8 | PCNT | 0.073 | 2.832 | 0.026 |
| H8Y6S5 | POLR2M | 0.517 | 1.855 | 0.279 |
| M0RBX6 | --- | 2.335 | 0.52 | 4.49 |
| D4A8F2 | RSU1 | 1.722 | 0.278 | 6.194 |
| D3ZJB8 | ARIH2 | 1.372 | 0.641 | 2.14 |
| D3ZJ86 | SLC9A6 | 1.373 | 0.617 | 2.225 |
| F1LNB3 | AKAP10 | 0.682 | 1.401 | 0.487 |
| A0A0G2K5L2 | LOC100911440 | 0.942 | 0.455 | 2.07 |
| D4A9W3 | DGLUCY | 1.096 | 0.337 | 3.252 |
| D4ADR5 | CHPF2 | 0.162 | 0.379 | 0.427 |
| E9PTJ1 | COIL | 0.368 | 2.424 | 0.152 |
| A0A0G2K3A5 | BIN3 | 1.416 | 0.584 | 2.425 |
| Q99P99 | HDAC4 | 0.452 | 1.548 | 0.292 |
| E9PTV9 | --- | 0.677 | 1.712 | 0.395 |

**Supplemental Table 8. The Gene Ontology Analysis based on different abundant proteins between IEC-6s treated with BM-exo and TNF-α-exo.**

| GO Terms Level | GO Terms Description | GO Terms ID | Mapping | Background | Fold Enrichment | Fisher' exact test P value |
| --- | --- | --- | --- | --- | --- | --- |
| 7 | hormone-mediated signaling pathway | GO:0009755 | 4/158 | 24/4751 | 5.01 | 0.007439 |
| 7 | osteoblast development | GO:0002076 | 2/158 | 5/4751 | 12.03 | 0.010287 |
| 7 | multi-organism membrane budding | GO:1902592 | 2/158 | 5/4751 | 12.03 | 0.010287 |
| 6 | late endosome to vacuole transport | GO:0045324 | 2/158 | 6/4751 | 10.02 | 0.015096 |
| 6 | positive regulation of chromatin binding | GO:0035563 | 2/158 | 6/4751 | 10.02 | 0.015096 |
| 7 | positive regulation of embryonic development | GO:0040019 | 2/158 | 7/4751 | 8.59 | 0.020676 |
| 6 | phosphorylation | GO:0016310 | 20/158 | 372/4751 | 1.62 | 0.020908 |
| 6 | regulation of protein complex disassembly | GO:0043244 | 4/158 | 34/4751 | 3.54 | 0.025129 |
| 7 | negative regulation of osteoblast differentiation | GO:0045668 | 2/158 | 8/4751 | 7.52 | 0.026973 |
| 6 | cell development | GO:0048468 | 17/158 | 311/4751 | 1.64 | 0.028084 |
| 6 | regulation of supramolecular fiber organization | GO:1902903 | 8/158 | 110/4751 | 2.19 | 0.029159 |
| 7 | cell morphogenesis involved in differentiation | GO:0000904 | 7/158 | 91/4751 | 2.31 | 0.030857 |
| 6 | intracellular signal transduction | GO:0035556 | 19/158 | 363/4751 | 1.57 | 0.030871 |
| 7 | negative regulation of protein complex disassembly | GO:0043242 | 3/158 | 21/4751 | 4.3 | 0.030923 |

**Supplemental Table 9. The Kyoto Encyclopedia of Genes and Genomes analysis based on different abundant proteins between IEC-6s treated with BM-exo and TNF-α-exo.**

| KEGG pathway | Mapping | Background | Fold enrichment | Fisher's exact test p value |
| --- | --- | --- | --- | --- |
| rno04260 Cardiac muscle contraction | 4/88 | 25/2706 | 4.92 | 0.007831 |
| rno04144 Endocytosis | 10/88 | 144/2706 | 2.14 | 0.01684 |
| rno00051 Fructose and mannose metabolism | 3/88 | 21/2706 | 4.39 | 0.028939 |
| rno05034 Alcoholism | 4/88 | 42/2706 | 2.93 | 0.045654 |

**Supplemental Table 10. Different abundant proteins between IEC-6s treated with HBM-exo and BM-exo.**

| Protein accession | Protein name | Relative expression (IEC-6s treated with HBM-exo) | Relative expression (IEC-6s treated with BM-exo) | Fold Change (H/M) |
| --- | --- | --- | --- | --- |
| P23358 | RPL12 | 1.314 | 0.308 | 4.266 |
| A0A0G2K8H0 | CAPRIN1 | 0.581 | 1.216 | 0.478 |
| Q6B345 | S100A11 | 0.096 | 2.619 | 0.037 |
| B5DEN5 | EEF1B2 | 1.771 | 0.343 | 5.163 |
| D3ZRM9 | LOC100360491 | 0.598 | 1.4 | 0.427 |
| A0JPM9 | EIF3J | 0.664 | 1.419 | 0.468 |
| M0RDD7 | CHTOPL1 | 1.429 | 0.636 | 2.247 |
| P61354 | RPL27 | 0.193 | 1.398 | 0.138 |
| Q9R1N3 | SLC4A7 | 0.379 | 1.945 | 0.195 |
| Z4YNF4 | ACP1 | 0.648 | 1.422 | 0.456 |
| Q5XIF6 | TUBA4A | 0.453 | 1.247 | 0.363 |
| M0R402 | TMX3 | 0.75 | 0.356 | 2.107 |
| F1M4U9 | BAZ1A | 0.56 | 1.517 | 0.369 |
| B2GUW4 | EXD2 | 1.779 | 0.484 | 3.676 |
| Q6P9Y4 | SLC25A4 | 0.485 | 1.248 | 0.389 |
| Q9WU49 | CARHSP1 | 0.469 | 0.981 | 0.478 |
| Q4QQV8 | CHMP5 | 1.354 | 0.571 | 2.371 |
| Q4FZU6 | ANXA8 | 0.55 | 1.366 | 0.403 |
| Q6P503 | ATP6V1D | 1.437 | 0.318 | 4.519 |
| F1M8F4 | GMFG | 0.427 | 1.586 | 0.269 |
| F8WG67 | ACOT7 | 1.403 | 0.354 | 3.963 |
| Q9EQH5 | CTBP2 | 0.581 | 1.446 | 0.402 |
| A0A0A0MY00 | ACADSB | 0.38 | 1.294 | 0.294 |
| G3V734 | DECR1 | 1.823 | 0.215 | 8.479 |
| Q9ESZ0 | XRCC1 | 1.475 | 0.637 | 2.316 |
| Q4KLH4 | PSPC1 | 1.37 | 0.626 | 2.188 |
| G3V762 | TSTA3 | 1.369 | 0.552 | 2.48 |
| A0A140UHX1 | LRWD1 | 1.526 | 0.582 | 2.622 |
| Q498D1 | FOXP1 | 0.364 | 1.387 | 0.262 |
| P11980 | PKM | 1.864 | 0.335 | 5.564 |
| D4AEG3 | PPIL4 | 1.947 | 0.569 | 3.422 |
| Q4QQU6 | SMNDC1 | 1.38 | 0.173 | 7.977 |
| A0A1W2Q6Q2 | DCLK1 | 0.449 | 1.089 | 0.412 |
| Q765A7 | PGAP1 | 0.456 | 1.107 | 0.412 |
| D3ZU64 | VPS37B | 1.019 | 0.488 | 2.088 |
| G3V8H5 | IKBKB | 1.843 | 0.402 | 4.585 |
| G3V8M5 | PPP4C | 1.614 | 0.461 | 3.501 |
| A0A0G2K3M7 | PHF10 | 1.437 | 0.671 | 2.142 |
| A0A0G2KAS7 | SLC1A3 | 1.553 | 0.612 | 2.538 |
| P68370 | TUBA1A | 0.119 | 1.043 | 0.114 |
| Q2KJ09 | USP16 | 0.571 | 1.366 | 0.418 |
| D3ZV82 | LOC685067 | 0.655 | 1.36 | 0.482 |
| Q0R3X4 | GIMAP4 | 0.458 | 1.363 | 0.336 |
| A0A0G2K4R1 | PPP1R12C | 0.429 | 0.89 | 0.482 |
| B5DF62 | PAK4 | 0.431 | 1.063 | 0.405 |
| D3ZUT9 | INTS3 | 2.194 | 0.611 | 3.591 |
| M0R7E6 | --- | 1.426 | 0.069 | 20.667 |
| D4A8C8 | PAXBP1 | 0.644 | 1.38 | 0.467 |
| G3V6R5 | SUOX | 1.534 | 0.462 | 3.32 |
| D3ZCL3 | SNRPC | 1.282 | 0.541 | 2.37 |
| D3ZY44 | MRPS2 | 0.45 | 1.683 | 0.267 |
| G3V7X3 | NCK1 | 0.604 | 1.429 | 0.423 |
| D3ZRV0 | DCUN1D1 | 0.435 | 1.104 | 0.394 |
| Q62671 | UBR5 | 1.609 | 0.778 | 2.068 |
| D4A2T4 | RGD1305184 | 0.503 | 1.592 | 0.316 |
| D4A2Y9 | PEX13 | 1.965 | 0.238 | 8.256 |
| D3ZUZ4 | FAM20B | 1.419 | 0.65 | 2.183 |
| D3ZJT9 | L3MBTL3 | 1.494 | 0.537 | 2.782 |
| E9PSX8 | SIPA1 | 0.559 | 1.338 | 0.418 |
| Q4V7D1 | SSR1 | 0.779 | 1.85 | 0.421 |
| A0A0G2JUK2 | LSM14A | 1.881 | 0.294 | 6.398 |
| D3ZUV9 | CNOT3 | 0.606 | 1.533 | 0.395 |
| P97546 | NPTN | 1.544 | 0.455 | 3.393 |
| Q5XII9 | MTFR1L | 1.521 | 0.576 | 2.641 |
| A0A140TAB3 | KLC1 | 1.386 | 0.63 | 2.2 |
| Q5M9F3 | RRP1 | 1.479 | 0.705 | 2.098 |
| D3ZDZ7 | FUK | 0.655 | 1.575 | 0.416 |
| Q923W4 | HDGFL3 | 1.388 | 0.226 | 6.142 |
| D3ZGJ0 | DHX32 | 0.451 | 1.752 | 0.257 |
| A0A096MJ42 | DLG3 | 1.856 | 0.335 | 5.54 |
| Q5FWY5 | AIP | 0.619 | 1.636 | 0.378 |
| A0A0G2JU83 | LBR | 0.25 | 0.994 | 0.252 |
| B3DMA5 | CNOT7 | 1.396 | 0.637 | 2.192 |
| Q497A9 | EIF4EBP2 | 0.725 | 0.342 | 2.12 |
| Q8K1P9 | FADS3 | 1.895 | 0.287 | 6.603 |
| Q9JHW1 | CPD | 0.412 | 1.264 | 0.326 |
| D3ZLP9 | SAP130 | 0.658 | 1.448 | 0.454 |
| D3ZHX6 | EYA3 | 0.466 | 1.591 | 0.293 |
| A0A096MIS5 | TMEM168 | 1.457 | 0.6 | 2.428 |
| Q4V8F5 | TADA3 | 1.328 | 0.654 | 2.031 |
| Q810U0 | CCDC50 | 0.336 | 1.252 | 0.268 |
| A0A140TAF0 | TPM3 | 1.81 | 0.665 | 2.722 |
| O35092 | TIMM17A | 1.661 | 0.339 | 4.9 |
| A0A0G2K4Y6 | HAUS6 | 1.454 | 0.505 | 2.879 |
| F1LPI5 | LAMB3 | 1.715 | 0.676 | 2.537 |
| A0A0G2K1Z2 | ARHGAP12 | 1.573 | 0.427 | 3.684 |
| D3ZGY2 | OTUD6B | 0.311 | 1.613 | 0.193 |
| Q9ERH3 | WDR7 | 0.603 | 1.226 | 0.492 |
| D4A986 | MBD2 | 1.516 | 0.48 | 3.158 |
| B2RYN4 | POLE4 | 0.446 | 1.554 | 0.287 |
| A1L108 | ARPC5L | 1.237 | 0.22 | 5.623 |
| G3V6R7 | OXSM | 1.148 | 0.562 | 2.043 |
| G3V8F7 | GGA2 | 0.645 | 1.368 | 0.471 |
| D4A8G0 | LSM12 | 0.281 | 1.357 | 0.207 |
| G3V722 | B4GALT1 | 1.318 | 0.638 | 2.066 |
| R9PXV8 | WRNIP1 | 0.599 | 1.498 | 0.4 |
| Q0D2L2 | MRPS22 | 1.87 | 0.616 | 3.036 |
| M0RCP9 | PIN4 | 0.657 | 1.326 | 0.495 |
| G3V7F5 | NR2C2 | 1.341 | 0.551 | 2.434 |
| D3ZG43 | NDUFS3 | 0.561 | 1.246 | 0.45 |
| D3ZIY3 | YTHDF3 | 0.518 | 1.157 | 0.448 |
| Q3MIF0 | RPE | 0.323 | 1.791 | 0.18 |
| D4AAI2 | ARHGAP45 | 0.549 | 1.91 | 0.287 |
| A0A0G2JUU6 | EXOSC9 | 1.646 | 0.817 | 2.015 |
| F1LW69 | WAC | 1.371 | 0.616 | 2.226 |
| D4A1B8 | DCTN3 | 0.517 | 1.038 | 0.498 |
| D4A1Z7 | TTF1 | 0.656 | 1.344 | 0.488 |
| D4A1G8 | CEP170B | 0.525 | 1.859 | 0.282 |
| F7EV88 | PBX1 | 0.327 | 1.307 | 0.25 |
| Q66HT5 | CYR61 | 0.48 | 1.52 | 0.316 |
| Q6GMM8 | SLC27A1 | 1.803 | 0.456 | 3.954 |
| P70531 | EEF2K | 0.567 | 1.433 | 0.396 |
| F1LPC5 | TMEM259 | 0.564 | 1.436 | 0.393 |
| B0BN99 | HMGB3 | 0.625 | 1.443 | 0.433 |
| D3ZUY0 | RDH14 | 1.429 | 0.713 | 2.004 |
| Q9R1A0 | CCNH | 0.332 | 0.924 | 0.359 |
| B2RZ68 | DCAF7 | 1.094 | 0.486 | 2.251 |
| Q5PPH0 | ENOPH1 | 0.381 | 1.331 | 0.286 |
| Q5XFW4 | MRPL13 | 0.68 | 1.775 | 0.383 |
| A0A0G2K3M6 | ATP9B | 0.437 | 1.187 | 0.368 |
| D3ZH66 | TAF5 | 1.066 | 0.531 | 2.008 |
| B5DF11 | ZFAND5 | 1.326 | 0.333 | 3.982 |
| D4AAE9 | CISD2 | 1.724 | 0.794 | 2.171 |
| B5DFI8 | UBE2S | 0.671 | 1.801 | 0.373 |
| Q63624 | SCAF1 | 1.717 | 0.83 | 2.069 |
| Q66H20 | PTBP2 | 0.584 | 1.182 | 0.494 |
| A0A0G2JWV6 | FAM219B | 0.378 | 1.272 | 0.297 |
| B5DEM7 | SUPT4H1 | 0.508 | 1.486 | 0.342 |
| Q5FWT9 | SIKE1 | 1.377 | 0.585 | 2.354 |
| A0A0H2UHR9 | STN1 | 1.161 | 0.485 | 2.394 |
| D4A731 | ANKIB1 | 1.339 | 0.661 | 2.026 |
| F7F3Z1 | LMAN2L | 1.787 | 0.213 | 8.39 |
| D3ZDJ4 | UNC93B1 | 0.375 | 1.388 | 0.27 |
| D4ADM2 | PAN3 | 0.28 | 0.96 | 0.292 |
| B1WC85 | WDR92 | 0.472 | 1.528 | 0.309 |
| O35815 | ATXN3 | 0.359 | 1.143 | 0.314 |
| G3V728 | NIPSNAP1 | 0.391 | 1.75 | 0.223 |
| D4A6T2 | RPUSD3 | 1.217 | 0.602 | 2.022 |
| Q6P727 | MAPK9 | 0.378 | 1.397 | 0.271 |
| F7FA68 | --- | 0.457 | 1.421 | 0.322 |
| D3ZRG0 | TOPBP1 | 0.374 | 1.19 | 0.314 |
| A0A0G2JZJ5 | PTDSS1 | 1.735 | 0.473 | 3.668 |
| F1LY69 | IFT140 | 0.43 | 1.345 | 0.32 |
| D3ZV30 | POLR3B | 1.625 | 0.375 | 4.333 |
| F2Z3S3 | ST6GAL1 | 0.745 | 1.822 | 0.409 |
| D4AD31 | PHF8 | 0.508 | 1.194 | 0.425 |
| A0A0G2KB61 | PPP1R13B | 0.542 | 1.458 | 0.372 |
| P70608 | SLC7A1 | 1.718 | 0.5 | 3.436 |
| D3ZZ04 | METTL9 | 1.52 | 0.67 | 2.269 |
| Q9Z2X3 | PSMD10 | 0.651 | 1.304 | 0.499 |
| F1M4J0 | RICTOR | 0.66 | 1.566 | 0.421 |
| B5DFE0 | MPP6 | 1.65 | 0.265 | 6.226 |
| G3V7B6 | PTPMT1 | 0.573 | 1.927 | 0.297 |
| Q3KRC4 | GPRC5C | 0.093 | 1.359 | 0.068 |
| Q5XIQ5 | SDAD1 | 1.735 | 0.646 | 2.686 |
| A0A096MK54 | SYNM | 1.676 | 0.567 | 2.956 |
| A0A0H2UI02 | STK11 | 0.596 | 1.421 | 0.419 |
| A0A0G2K8X3 | PID1 | 1.468 | 0.694 | 2.115 |
| D3ZAW4 | ABHD4 | 1.42 | 0.58 | 2.448 |
| Q5PQN7 | LZIC | 0.448 | 1.552 | 0.289 |
| Q6AYG3 | PRUNE1 | 0.444 | 1.57 | 0.283 |
| Q7TSE9 | HAX1 | 2.339 | 0.417 | 5.609 |
| F7ESD4 | RNF126 | 0.246 | 1.282 | 0.192 |
| D3ZPN3 | MLF2 | 1.759 | 0.241 | 7.299 |
| D3ZM59 | USP45 | 1.228 | 0.491 | 2.501 |
| Q76JQ4 | BHLHE40 | 1.662 | 0.338 | 4.917 |
| F1LVX1 | DNAJC1 | 1.406 | 0.624 | 2.253 |
| A0A0G2JVR3 | IGHMBP2 | 0.635 | 1.365 | 0.465 |
| M0RBX6 | --- | 0.145 | 2.335 | 0.062 |
| O35532 | MSMO1 | 1.398 | 0.602 | 2.322 |
| F1LU70 | GPATCH1 | 1.617 | 0.59 | 2.741 |
| Q5PQN9 | MRPL38 | 0.558 | 1.205 | 0.463 |
| D4ADR5 | CHPF2 | 2.46 | 0.162 | 15.185 |
| A0A0G2JV78 | OSBPL5 | 1.685 | 0.315 | 5.349 |
| F1M787 | CTNND2 | 1.725 | 0.275 | 6.273 |
| D4AAU7 | LRRC9 | 2.209 | 0.288 | 7.67 |
| Q8K3X8 | HSBP1 | 1.443 | 0.557 | 2.591 |
| A0A0G2JXI7 | FOXP4 | 0.45 | 1.55 | 0.29 |
| Q6XFR6 | GYPC | 1.909 | 0.663 | 2.879 |
| D3Z8M8 | TSC22D1 | 1.766 | 0.234 | 7.547 |

**Supplemental Table 11. The Gene Ontology Analysis based on different abundant proteins between IEC-6s treated with HBM-exo and BM-exo.**

| GO Terms Level | GO Terms Description | GO Terms ID | Mapping | Background | Fold Enrichment | Fisher' exact test P value |
| --- | --- | --- | --- | --- | --- | --- |
| 6 | smooth muscle tissue development | GO:0048745 | 2/148 | 4/4751 | 16.05 | 0.00555 |
| 6 | positive regulation of chromatin binding | GO:0035563 | 2/148 | 6/4751 | 10.7 | 0.013315 |
| 7 | TOR signaling | GO:0031929 | 2/148 | 9/4751 | 7.13 | 0.030055 |
| 7 | protein localization to cilium | GO:0061512 | 2/148 | 9/4751 | 7.13 | 0.030055 |
| 6 | intermediate filament cytoskeleton organization | GO:0045104 | 2/148 | 9/4751 | 7.13 | 0.030055 |
| 7 | cellular response to misfolded protein | GO:0071218 | 2/148 | 11/4751 | 5.84 | 0.044089 |

**Supplemental Table 12. The Kyoto Encyclopedia of Genes and Genomes analysis based on different abundant proteins between IEC-6s treated with HBM-exo and BM-exo.**

| KEGG pathway | Mapping | Background | Fold enrichment | Fisher's exact test p value |
| --- | --- | --- | --- | --- |
| rno04930 Type II diabetes mellitus | 3/81 | 13/2706 | 7.71 | 0.005951 |
| rno04920 Adipocytokine signaling pathway | 3/81 | 27/2706 | 3.71 | 0.04509 |
| rno04622 RIG-I-like receptor signaling pathway | 3/81 | 28/2706 | 3.58 | 0.049441 |

**Supplemental Table 13. Different abundant proteins between IEC-6s treated with HBM-exo and TNF-α-exo.**

| Protein accession | Protein name | Relative expression (IEC-6s treated with HBM-exo) | Relative expression (IEC-6s treated with TNF-α-exo) | Fold Change (H/T) |
| --- | --- | --- | --- | --- |
| P05065 | ALDOA | 0.694 | 1.603 | 0.433 |
| P13084 | NPM1 | 1.284 | 0.575 | 2.233 |
| P54001 | P4HA1 | 1.389 | 0.68 | 2.043 |
| P21531 | RPL3 | 1.316 | 0.654 | 2.012 |
| P05982 | NQO1 | 1.093 | 0.508 | 2.152 |
| A0A0G2K8H0 | CAPRIN1 | 0.581 | 1.202 | 0.483 |
| F1LUM5 | TUBAL3 | 1.371 | 0.238 | 5.761 |
| Q6B345 | S100A11 | 0.096 | 0.285 | 0.337 |
| P62755 | RPS6 | 0.554 | 2.059 | 0.269 |
| A0A096MJB3 | WDR82 | 1.043 | 0.483 | 2.159 |
| P61354 | RPL27 | 0.193 | 1.409 | 0.137 |
| Q5XIF6 | TUBA4A | 0.453 | 1.3 | 0.348 |
| P47853 | BGN | 1.422 | 0.613 | 2.32 |
| G3V7B0 | NOL9 | 1.52 | 0.615 | 2.472 |
| D3ZPL1 | CPSF6 | 1.474 | 0.113 | 13.044 |
| M0R402 | TMX3 | 0.75 | 1.894 | 0.396 |
| A0A140TAA8 | NDC1 | 1.251 | 0.564 | 2.218 |
| B2GUW4 | EXD2 | 1.779 | 0.736 | 2.417 |
| Q6P9Y4 | SLC25A4 | 0.485 | 1.267 | 0.383 |
| Q5U2X0 | CDKN2AIP | 1.25 | 0.622 | 2.01 |
| Q9WU49 | CARHSP1 | 0.469 | 1.549 | 0.303 |
| A0A0G2JUV4 | MRPL1 | 1.445 | 0.309 | 4.676 |
| F1LNE2 | UNC5B | 1.398 | 0.612 | 2.284 |
| B5DFM8 | BCAS2 | 1.238 | 0.611 | 2.026 |
| Q9R1E9 | CTGF | 1.618 | 0.466 | 3.472 |
| B0BNA5 | COTL1 | 0.547 | 1.562 | 0.35 |
| D4A3V3 | PARP12 | 0.655 | 1.417 | 0.462 |
| F1M8F4 | GMFG | 0.427 | 0.987 | 0.433 |
| A0A0G2K8Z9 | KIF13B | 0.64 | 1.305 | 0.49 |
| Q6AXT8 | SF3A2 | 1.334 | 0.63 | 2.117 |
| D3ZA85 | NFU1 | 1.442 | 0.502 | 2.873 |
| Q5PPG2 | LGMN | 1.262 | 0.604 | 2.089 |
| A0A0A0MY00 | ACADSB | 0.38 | 1.326 | 0.287 |
| A0A0G2K508 | RRAS2 | 1.204 | 0.49 | 2.457 |
| F1M978 | IMPA1 | 0.736 | 1.544 | 0.477 |
| Q4KM31 | LIMD2 | 1.293 | 0.638 | 2.027 |
| Q5XJW2 | GADD45GIP1 | 1.246 | 0.474 | 2.629 |
| Q6AYD9 | NUDT19 | 0.477 | 2.106 | 0.226 |
| Q498D1 | FOXP1 | 0.364 | 1.25 | 0.291 |
| Q62785 | PDAP1 | 1.507 | 0.509 | 2.961 |
| D3ZUI1 | APIP | 0.651 | 1.342 | 0.485 |
| D3ZT98 | BOLA3 | 1.527 | 0.264 | 5.784 |
| A0A0G2JY75 | GTF3C2 | 1.429 | 0.508 | 2.813 |
| P11980 | PKM | 1.864 | 0.801 | 2.327 |
| B1H284 | TAF5L | 1.343 | 0.645 | 2.082 |
| Q91V33 | KHDRBS1 | 1.428 | 0.662 | 2.157 |
| D4AEG3 | PPIL4 | 1.947 | 0.484 | 4.023 |
| A0A1W2Q6Q2 | DCLK1 | 0.449 | 1.462 | 0.307 |
| A0A0G2K2M2 | LOC102556337 | 0.709 | 1.421 | 0.499 |
| D3ZUD8 | TM9SF3 | 1.554 | 0.414 | 3.754 |
| P13233 | CNP | 1.28 | 0.563 | 2.274 |
| Q765A7 | PGAP1 | 0.456 | 1.437 | 0.317 |
| G3V8H5 | IKBKB | 1.843 | 0.756 | 2.438 |
| D3ZPD0 | CKAP2 | 1.403 | 0.506 | 2.773 |
| F1LPQ3 | MAN2C1 | 0.747 | 1.5 | 0.498 |
| P68370 | TUBA1A | 0.119 | 1.838 | 0.065 |
| A0A0G2K777 | RHOT1 | 1.453 | 0.522 | 2.784 |
| Q6IRE4 | TSG101 | 1.169 | 0.453 | 2.581 |
| D4ACW0 | RBM6 | 0.51 | 1.571 | 0.325 |
| D3ZGW2 | AP1G2 | 0.557 | 1.393 | 0.4 |
| Q7TSU1 | ARFGEF2 | 1.368 | 0.529 | 2.586 |
| Q0R3X4 | GIMAP4 | 0.458 | 1.179 | 0.388 |
| A0A0G2K4R1 | PPP1R12C | 0.429 | 1.681 | 0.255 |
| Q5RJN0 | NDUFS7 | 0.703 | 1.533 | 0.459 |
| F1M771 | RYBP | 1.415 | 0.424 | 3.337 |
| B5DF62 | PAK4 | 0.431 | 1.506 | 0.286 |
| Q810T5 | KAT7 | 1.202 | 0.588 | 2.044 |
| P62489 | POLR2G | 0.695 | 1.42 | 0.489 |
| D3ZUT9 | INTS3 | 2.194 | 0.196 | 11.194 |
| D3ZFL3 | MAP3K6 | 0.582 | 2.029 | 0.287 |
| D3ZRV0 | DCUN1D1 | 0.435 | 1.461 | 0.298 |
| Q62671 | UBR5 | 1.609 | 0.613 | 2.625 |
| A0A0G2K7P2 | PHF14 | 1.192 | 0.536 | 2.224 |
| Q4QRB4 | TUBB3 | 1.333 | 0.54 | 2.469 |
| B0BN83 | ARMC1 | 1.294 | 0.474 | 2.73 |
| D4A2Y9 | PEX13 | 1.965 | 0.797 | 2.465 |
| D3ZTX0 | TMED7 | 0.554 | 1.556 | 0.356 |
| D3ZFQ3 | NUBPL | 1.335 | 0.665 | 2.008 |
| Q9R0Z7 | AAGAB | 0.638 | 1.352 | 0.472 |
| Q4V7D1 | SSR1 | 0.779 | 0.371 | 2.1 |
| A0A0G2JUK2 | LSM14A | 1.881 | 0.825 | 2.28 |
| A0A096MJ98 | --- | 0.415 | 2.081 | 0.199 |
| A0A0G2K588 | LTBP4 | 1.676 | 0.435 | 3.853 |
| D4A563 | PEAK1 | 1.409 | 0.307 | 4.59 |
| Q6MFY6 | PPP1R11 | 0.594 | 1.362 | 0.436 |
| Q62662 | FRK | 1.394 | 0.629 | 2.216 |
| B1H249 | GNPNAT1 | 0.704 | 1.463 | 0.481 |
| A0A0G2JYN0 | DCAF8 | 0.742 | 1.553 | 0.478 |
| Q7M6Z3 | SMYD2 | 0.685 | 1.511 | 0.453 |
| A0A0G2K9M4 | WDFY3 | 1.095 | 0.512 | 2.139 |
| B2RYQ2 | PTPA | 1.339 | 0.412 | 3.25 |
| D4A3Z4 | GCFC2 | 0.65 | 1.304 | 0.498 |
| A0A0G2K9R0 | ZSWIM8 | 1.426 | 0.597 | 2.389 |
| A0A096MJ42 | DLG3 | 1.856 | 0.809 | 2.294 |
| B1WC25 | TRA2A | 1.314 | 0.591 | 2.223 |
| Q5M7U2 | ORC5 | 0.633 | 1.608 | 0.394 |
| A0A0G2JU83 | LBR | 0.25 | 1.755 | 0.142 |
| Q6AZ50 | ATG3 | 0.755 | 1.713 | 0.441 |
| Q4QR81 | RBMS2 | 1.41 | 0.638 | 2.21 |
| Q497A9 | EIF4EBP2 | 0.725 | 1.932 | 0.375 |
| Q8K1P9 | FADS3 | 1.895 | 0.818 | 2.317 |
| Q6MGC4 | PFDN6 | 0.625 | 1.327 | 0.471 |
| Q3B8N7 | TSC22D4 | 0.515 | 1.61 | 0.32 |
| Q9JHW1 | CPD | 0.412 | 1.325 | 0.311 |
| Q5RK19 | SNF8 | 1.198 | 0.545 | 2.198 |
| D3ZHX6 | EYA3 | 0.466 | 0.943 | 0.494 |
| Q810U0 | CCDC50 | 0.336 | 1.412 | 0.238 |
| A0A140TAF0 | TPM3 | 1.81 | 0.525 | 3.448 |
| A0A0G2JWL3 | NF1 | 1.346 | 0.435 | 3.094 |
| D4ABP9 | FBXO3 | 0.575 | 1.896 | 0.303 |
| G3V6K4 | DCAF5 | 0.482 | 1.518 | 0.318 |
| D4ACK7 | CNNM3 | 0.591 | 1.409 | 0.419 |
| F1LPI5 | LAMB3 | 1.715 | 0.609 | 2.816 |
| D3ZGY2 | OTUD6B | 0.311 | 1.076 | 0.289 |
| D3ZH92 | KLHL26 | 1.417 | 0.246 | 5.76 |
| F1M7K9 | --- | 1.268 | 0.39 | 3.251 |
| A0A0G2KA71 | NECTIN2 | 1.351 | 0.655 | 2.063 |
| D4A8G0 | LSM12 | 0.281 | 1.363 | 0.206 |
| Q5BKC6 | HSPBAP1 | 0.521 | 2.148 | 0.243 |
| D3ZKX1 | WASHC3 | 0.609 | 1.439 | 0.423 |
| Q3MHT4 | TUT1 | 1.611 | 0.527 | 3.057 |
| D4A2N0 | HMGXB4 | 1.275 | 0.636 | 2.005 |
| Q0D2L2 | MRPS22 | 1.87 | 0.514 | 3.638 |
| Q4V888 | PIP4P2 | 0.56 | 1.521 | 0.368 |
| P00406 | MTCO2 | 0.765 | 1.798 | 0.425 |
| D3ZG43 | NDUFS3 | 0.561 | 1.192 | 0.471 |
| A0A0G2K351 | UNC119B | 1.389 | 0.419 | 3.315 |
| D3ZIY3 | YTHDF3 | 0.518 | 1.326 | 0.391 |
| Q3MIF0 | RPE | 0.323 | 0.886 | 0.365 |
| A0A0G2JUU6 | EXOSC9 | 1.646 | 0.537 | 3.065 |
| D4A1B8 | DCTN3 | 0.517 | 1.445 | 0.358 |
| Q6MGB8 | RT1-A2 | 0.585 | 1.251 | 0.468 |
| Q5XII0 | EPDR1 | 1.507 | 0.67 | 2.249 |
| F1M9C8 | KIF1C | 1.065 | 0.422 | 2.524 |
| M0R750 | SRCAP | 0.697 | 1.598 | 0.436 |
| D4A3T2 | GATAD1 | 1.181 | 0.585 | 2.019 |
| F7EV88 | PBX1 | 0.327 | 1.365 | 0.24 |
| M0RA39 | MXRA7 | 1.457 | 0.191 | 7.628 |
| E9PT24 | ZEB1 | 1.816 | 0.184 | 9.87 |
| A0A0G2K5Y1 | VIPAS39 | 0.57 | 1.318 | 0.432 |
| D3ZT03 | UPF2 | 0.667 | 1.415 | 0.471 |
| G3V721 | WBP2 | 1.453 | 0.625 | 2.325 |
| Q6GMM8 | SLC27A1 | 1.803 | 0.741 | 2.433 |
| D3ZQJ3 | GIGYF1 | 0.939 | 0.446 | 2.105 |
| Q9ESM0 | IP6K1 | 1.359 | 0.455 | 2.987 |
| F1LQN9 | EXOC1 | 0.62 | 1.393 | 0.445 |
| A0A0G2K590 | SPAST | 0.752 | 1.583 | 0.475 |
| B5DF37 | TAF6L | 1.628 | 0.511 | 3.186 |
| D3ZAZ5 | BCAR3 | 1.524 | 0.5 | 3.048 |
| Q9R1A0 | CCNH | 0.332 | 1.744 | 0.19 |
| Q5PPH0 | ENOPH1 | 0.381 | 1.288 | 0.296 |
| A0A0G2K3M6 | ATP9B | 0.437 | 1.376 | 0.318 |
| D4AAE9 | CISD2 | 1.724 | 0.482 | 3.577 |
| A0A0G2K4R7 | RBMS1 | 0.696 | 1.669 | 0.417 |
| Q63624 | SCAF1 | 1.717 | 0.453 | 3.79 |
| D4AAM1 | RGD1597339 | 1.316 | 0.524 | 2.511 |
| Q66H20 | PTBP2 | 0.584 | 1.233 | 0.474 |
| Q6IN24 | LGALS8 | 0.194 | 1.806 | 0.107 |
| A0A0G2JWV6 | FAM219B | 0.378 | 1.35 | 0.28 |
| G3V8H7 | OLFML3 | 1.372 | 0.502 | 2.733 |
| A0A0G2K038 | REXO2 | 1.302 | 0.413 | 3.153 |
| A0A0G2JUF1 | PECR | 0.531 | 1.469 | 0.361 |
| D3ZEH2 | FOXRED1 | 1.302 | 0.598 | 2.177 |
| D3ZDJ4 | UNC93B1 | 0.375 | 1.237 | 0.303 |
| D4ADM2 | PAN3 | 0.28 | 1.76 | 0.159 |
| A0A0G2K1A9 | NOL11 | 1.157 | 0.459 | 2.521 |
| Q5RK28 | NMES1 | 1.427 | 0.573 | 2.49 |
| M0RBJ0 | GNG2 | 1.118 | 0.532 | 2.102 |
| F1M5V7 | MEX3D | 0.477 | 1.523 | 0.313 |
| O35815 | ATXN3 | 0.359 | 1.498 | 0.24 |
| G3V728 | NIPSNAP1 | 0.391 | 0.86 | 0.455 |
| Q5M9I5 | UQCRH | 1.694 | 0.402 | 4.214 |
| F1LU48 | ERGIC1 | 1.622 | 0.198 | 8.192 |
| Q6P727 | MAPK9 | 0.378 | 1.225 | 0.309 |
| D3ZDR9 | NRDE2 | 0.579 | 1.472 | 0.393 |
| A0A0G2JSX1 | NSMCE1 | 1.439 | 0.564 | 2.551 |
| Q5U2N8 | TTC26 | 0.592 | 1.507 | 0.393 |
| D3ZHF8 | GUF1 | 1.364 | 0.483 | 2.824 |
| F7FA68 | --- | 0.457 | 1.122 | 0.407 |
| D3ZRG0 | TOPBP1 | 0.374 | 1.436 | 0.26 |
| D4AEL8 | CEP131 | 1.17 | 0.374 | 3.128 |
| A0A0G2JZJ5 | PTDSS1 | 1.735 | 0.792 | 2.191 |
| F1LY69 | IFT140 | 0.43 | 1.226 | 0.351 |
| A0A0G2JWC6 | RGD621098 | 0.554 | 1.609 | 0.344 |
| Q6P777 | MVB12A | 1.381 | 0.523 | 2.641 |
| F1LWT0 | SIMC1 | 0.6 | 1.708 | 0.351 |
| M0R5F8 | HMGN5 | 0.311 | 1.689 | 0.184 |
| D4AD31 | PHF8 | 0.508 | 1.298 | 0.391 |
| Q5XIF3 | NDUFS4 | 1.54 | 0.488 | 3.156 |
| Q9QX19 | CIT | 1.033 | 0.485 | 2.13 |
| P70608 | SLC7A1 | 1.718 | 0.782 | 2.197 |
| D3ZYT2 | MRPS5 | 1.611 | 0.559 | 2.882 |
| A0A0G2JVD0 | RBM28 | 1.049 | 0.303 | 3.462 |
| A0A0G2K0T2 | CD63 | 0.679 | 1.814 | 0.374 |
| Q3KRC4 | GPRC5C | 0.093 | 1.549 | 0.06 |
| Q5XIQ5 | SDAD1 | 1.735 | 0.619 | 2.803 |
| D3Z9G0 | TRAF3 | 0.149 | 2.705 | 0.055 |
| Q5KTC7 | NAAA | 1.504 | 0.496 | 3.032 |
| Q64542 | ATP2B4 | 1.485 | 0.515 | 2.883 |
| A0A096MK54 | SYNM | 1.676 | 0.756 | 2.217 |
| F7DLY1 | EPS8L2 | 0.998 | 0.181 | 5.514 |
| Q6AYG3 | PRUNE1 | 0.444 | 0.986 | 0.45 |
| Q5M843 | OGFOD3 | 1.265 | 0.596 | 2.122 |
| A0A0G2JSR9 | SDF4 | 1.282 | 0.543 | 2.361 |
| Q7TSE9 | HAX1 | 2.339 | 0.244 | 9.586 |
| F7ESD4 | RNF126 | 0.246 | 1.472 | 0.167 |
| D3ZT89 | LDB1 | 0.612 | 1.388 | 0.441 |
| Q07014 | LYN | 0.51 | 1.49 | 0.342 |
| F1M8K0 | DAG1 | 1.224 | 0.504 | 2.429 |
| B0BNM0 | TMEM222 | 1.46 | 0.261 | 5.594 |
| A0A096MJX5 | RGD1305178 | 1.338 | 0.496 | 2.698 |
| F1M9X4 | CLIC6 | 0.609 | 1.391 | 0.438 |
| B1H264 | TBC1D17 | 1.316 | 0.416 | 3.163 |
| D3ZMY8 | PCNT | 0.095 | 2.832 | 0.034 |
| H8Y6S5 | POLR2M | 0.628 | 1.855 | 0.339 |
| M0RBX6 | --- | 0.145 | 0.52 | 0.279 |
| D3Z822 | ATR | 1.878 | 0.122 | 15.393 |
| F1LU70 | GPATCH1 | 1.617 | 0.793 | 2.039 |
| D4A5D3 | BUB1 | 1.397 | 0.603 | 2.317 |
| Q5PQN9 | MRPL38 | 0.558 | 1.237 | 0.451 |
| Q9Z1Z1 | EIF2AK3 | 0.651 | 1.456 | 0.447 |
| A0A0G2K5L2 | LOC100911440 | 1.603 | 0.455 | 3.523 |
| D4A9W3 | DGLUCY | 1.567 | 0.337 | 4.65 |
| D4ADR5 | CHPF2 | 2.46 | 0.379 | 6.491 |
| E9PTJ1 | COIL | 0.208 | 2.424 | 0.086 |
| D4AAU7 | LRRC9 | 2.209 | 0.504 | 4.383 |
| E9PTV9 | --- | 0.611 | 1.712 | 0.357 |
| Q6XFR6 | GYPC | 1.909 | 0.428 | 4.46 |

**Supplemental Table 14. The Gene Ontology Analysis based on different abundant proteins between IEC-6s treated HBM-exo and TNF-α-exo.**

| GO Terms Level | GO Terms Description | GO Terms ID | Mapping | Background | Fold Enrichment | Fisher' exact test P value |
| --- | --- | --- | --- | --- | --- | --- |
| 7 | regulation of ERBB signaling pathway | GO:1901184 | 4/178 | 18/4751 | 5.93 | 0.003859 |
| 7 | microtubule-based protein transport | GO:0099118 | 3/178 | 10/4751 | 8.01 | 0.005108 |
| 7 | regulation of bicellular tight junction assembly | GO:2000810 | 2/178 | 5/4751 | 10.68 | 0.012954 |
| 7 | negative regulation of cell junction assembly | GO:1901889 | 2/178 | 6/4751 | 8.9 | 0.018956 |
| 6 | phosphorylation | GO:0016310 | 22/178 | 372/4751 | 1.58 | 0.020458 |
| 7 | regulation of organelle assembly | GO:1902115 | 5/178 | 44/4751 | 3.03 | 0.023221 |
| 7 | mitochondrion disassembly | GO:0061726 | 2/178 | 7/4751 | 7.63 | 0.025891 |
| 6 | regulation of mesenchymal cell proliferation | GO:0010464 | 2/178 | 7/4751 | 7.63 | 0.025891 |
| 7 | glycerophospholipid metabolic process | GO:0006650 | 6/178 | 65/4751 | 2.46 | 0.033745 |
| 6 | microtubule-based transport | GO:0099111 | 5/178 | 49/4751 | 2.72 | 0.035137 |
| 7 | transport along microtubule | GO:0010970 | 5/178 | 49/4751 | 2.72 | 0.035137 |
| 7 | glycerolipid biosynthetic process | GO:0045017 | 4/178 | 34/4751 | 3.14 | 0.036776 |
| 7 | cilium assembly | GO:0060271 | 4/178 | 35/4751 | 3.05 | 0.040349 |
| 6 | intermediate filament cytoskeleton organization | GO:0045104 | 2/178 | 9/4751 | 5.93 | 0.042254 |

**Supplemental Table 15. The Kyoto Encyclopedia of Genes and Genomes analysis based on different abundant proteins between IEC-6s treated with HBM-exo and TNF-α-exo.**

| KEGG pathway | Mapping | Background | Fold enrichment | Fisher's exact test p value |
| --- | --- | --- | --- | --- |
| rno04932 Non-alcoholic fatty liver disease (NAFLD) | 8/101 | 79/2706 | 2.71 | 0.008283 |
| rno04137 Mitophagy - animal | 5/101 | 35/2706 | 3.83 | 0.008733 |
| rno04930 Type II diabetes mellitus | 3/101 | 13/2706 | 6.18 | 0.010989 |
| rno05168 Herpes simplex infection | 8/101 | 87/2706 | 2.46 | 0.014512 |
| rno04210 Apoptosis | 7/101 | 75/2706 | 2.5 | 0.020263 |
| rno05167 Kaposi sarcoma-associated herpesvirus infection | 7/101 | 79/2706 | 2.37 | 0.026217 |

**Supplemental Table 16. The string network coordinates of High mobility group box 3 protein.**

| Node | Annotation |
| --- | --- |
| Polr3e | Polymerase (RNA) III (DNA directed) polypeptide E |
| Supt16h | SPT16 homolog, facilitates chromatin-remodeling subunit; Suppressor of Ty 16 homolog (S. cerevisiae) |
| Polr3a | DNA-directed RNA polymerase subunit; DNA-dependent RNA polymerase catalyzes the transcription of DNA into RNA using the four ribonucleoside triphosphates as substrates |
| LOC685069 | H2A histone family, member V |
| Polr3f | DNA-directed RNA polymerase III subunit RPC6; DNA-dependent RNA polymerase catalyzes the transcription of DNA into RNA using the four ribonucleoside triphosphates as substrates. Specific peripheric component of RNA polymerase III which synthesizes small RNAs, such as 5S rRNA and tRNAs |
| Smarca1 | SWI/SNF related, matrix associated, actin dependent regulator of chromatin, subfamily a, member 1 |
| ENSRNOG00000031564 | Histone H2A |
| Ssrp1 | FACT complex subunit SSRP1; Component of the FACT complex, a general chromatin factor that acts to reorganize nucleosomes. The FACT complex is involved in multiple processes that require DNA as a template such as mRNA elongation, DNA replication and DNA repair. During transcription elongation the FACT complex acts as a histone chaperone that both destabilizes and restores nucleosomal structure. It facilitates the passage of RNA polymerase II and transcription by promoting the dissociation of one histone H2A-H2B dimer from the nucleosome, then subsequently promotes the reestablishment. |
| Hmgb3 | High mobility group box 3 |
| H2afz | Histone H2A.Z; Variant histone H2A which replaces conventional H2A in a subset of nucleosomes. Nucleosomes wrap and compact DNA into chromatin, limiting DNA accessibility to the cellular machineries which require DNA as a template. Histones thereby play a central role in transcription regulation, DNA repair, DNA replication and chromosomal stability. DNA accessibility is regulated via a complex set of post-translational modifications of histones, also called histone code, and nucleosome remodeling. May be involved in the formation of constitutive heterochromatin. |
| Numa1 | Nuclear mitotic apparatus protein 1 |

**Supplemental Table 17. The predicted microRNAs capable of binding to the 3'UTR region of *Hmgb3* analyzed by mirWalk, miRBD and TargetScan.**

| Intersection set of mirWalk, miRBD and TargetScan | Intersection set of mirWalk and miRBD | Intersection set of mirWalk and TargetSan | Intersection set of miRBD and TargetScan |
| --- | --- | --- | --- |
| miR-139 | miR-409a | miR-214 | miR-429 |
| miR-17 | miR-344i | miR-124 | miR-200b |
| miR-200b | miR-295 | miR-128 | miR-139 |
|  | miR-200c | miR-139 | miR-17 |
|  | miR-200b | miR-141 |  |
|  | miR-191a | miR-17 |  |
|  | miR-17 | miR-200a |  |
|  | miR-139 | miR-200b |  |
|  | miR-702 | miR-93 |  |
|  | miR-628 | miR-539 |  |
|  | miR-582 | miR-412 |  |
|  | miR-452 | miR-375 |  |
|  |  | miR-351 |  |
|  |  | miR-350 |  |
|  |  | miR-345 |  |
|  |  | miR-217 |  |

**Supplemental Table 18. Sequencing reports of synthetic genes utilized for Luciferase Reporter Assay**

| Gene Name | Sequence number |
| --- | --- |
| *Hmgb3*_WT | >L5Y6303-1-GLO-F_A01.ab1 |
| *Hmgb3*_MUT | >L5Y6304-1-GLO-F_E01.ab1 |
| rno-miR-200b Positive Control | >L5Y6305-2-GLO-F_B02.ab1 |

>L5Y6303-1-GLO-F_A01.ab1NNNGNGNTNATCGTGGACTATGTGGCCAGCCAGGTTACAACCGCCAAGAAGCTGCGCGGTGGTGTTGTGTTCGTGGACGAGGTGCCTAAAGGACTGACCGGCAAGTTGGACGCCCGCAAGATCCGCGAGATTCTCATTAAGGCCAAGAAGGGCGGCAAGATCGCCGTGTAATTCTAGTTGTTTAAACGAGCTC***TGTATGTTTAGAATGCTGAAATGTTTTTGAAGTTAAATAAACAGTATTACATTTTTAAAA***CTCGAGTCTAGAGTCGACCTGCAGGCATGCAAGCTGATCCGGCTGCTAACAAAGCCCGAAAGGAAGCTGAGTTGGCTGCTGCCACCGCTGAGCAATAACTAGCATAACCCCTTGGGGCGGCCGCTTCGAGCAGACATGATAAGATACATTGATGAGTTTGGACAAACCACAACTAGAATGCAGTGAAAAAAATGCTTTATTTGTGAAATTTGTGATGCTATTGCTTTATTTGTAACCATTATAAGCTGCAATAAACAAGTTAACAACAACAATTGCATTCATTTTATGTTTCAGGTTCAGGGGGAGATGTGGGAGGTTTTTTTAAGCAAGTAAAACCTCTACAAATGTGGTAAAATCGAATTTTAACAAAATATTAACGCTTACAATTTCCTGATGCGGTATTTTCTCCTTACGCATCTGTGCGGTATTTCACACCGCATACGCGGATCTGCGCAGCACCATGGCCTGAAATAACCTCTGAAAGAGGAACTTGGTTAGGTACCTTCTGAGGCGGAAAGAACCAGCTGTGGAATGTGTGTCAGTTAGGGTGTGGAAAGTCCCCAGGCTCCCCAGCAGGCAGAAGTATGCAAAGCATGCATCTCAATTAGTCAGCAACCAGGTGTGGAAAGTCCCCAGGCTCCCCAGCAGGCAGAAGTATGCAAAGCATGCATCTCAATTAGTCAGCAACCATAGTCCCGCCCCTAACTCCGCCCATCCCGCCCCTAACTCCGCCCAGTTCCGCCCATTCTCCGCCCCATGGCTGACTAAATTTTTTTATTTATGCAGAGGCCGAGNCGCCTCGGCCTCTGAGCTATTCCAGAGTAGTGAGGAGGCCTTTTTGGAGGCTTAGCTTTTGCAAAAGCTGATTCTTCTGAANNNACAGTCTCGNNNNNCTGNAGCCACCATGNNTCNNGNGTACGACCCCGANNNACGNNNNNATGATCACTNNNNCAANNNNGGNCTCGCTGCAAGGCCAATGAACNNNGCCN

>L5Y6304-1-GLO-F_E01.ab1NNNNGNGNNACGTGGACTATGTGGCCAGCCAGGTTACAACCGCCAAGAAGCTGCGCGGTGGTGTTGTGTTCGTGGACGAGGTGCCTAAAGGACTGACCGGCAAGTTGGACGCCCGCAAGATCCGCGAGATTCTCATTAAGGCCAAGAAGGGCGGCAAGATCGCCGTGTAATTCTAGTTGTTTAAACGAGCTC***TGTATGTTTAGAATGCTGAAATGTTTTTGAAGTTAAATAAAGTCATAATCATTTTTAAAA***CTCGAGTCTAGAGTCGACCTGCAGGCATGCAAGCTGATCCGGCTGCTAACAAAGCCCGAAAGGAAGCTGAGTTGGCTGCTGCCACCGCTGAGCAATAACTAGCATAACCCCTTGGGGCGGCCGCTTCGAGCAGACATGATAAGATACATTGATGAGTTTGGACAAACCACAACTAGAATGCAGTGAAAAAAATGCTTTATTTGTGAAATTTGTGATGCTATTGCTTTATTTGTAACCATTATAAGCTGCAATAAACAAGTTAACAACAACAATTGCATTCATTTTATGTTTCAGGTTCAGGGGGAGATGTGGGAGGTTTTTTTAAGCAAGTAAAACCTCTACAAATGTGGTAAAATCGAATTTTAACAAAATATTAACGCTTACAATTTCCTGATGCGGTATTTTCTCCTTACGCATCTGTGCGGTATTTCACACCGCATACGCGGATCTGCGCAGCACCATGGCCTGAAATAACCTCTGAAAGAGGAACTTGGTTAGGTACCTTCTGAGGCGGAAAGAACCAGCTGTGGAATGTGTGTCAGTTAGGGTGTGGAAAGTCCCCAGGCTCCCCAGCAGGCAGAAGTATGCAAAGCATGCATCTCAATTAGTCAGCAACCAGGTGTGGAAAGTCCCCAGGCTCCCCAGCAGGCAGAAGTATGCAAAGCATGCATCTCAATTAGTCAGCAACCATAGTCCCGCCCCTAACTCCGCCCATCCCGCCCCTAACTCCGCCCAGTTCCGCCCATTCTCCGCCCCATGGCTGACTAATTTTTTTATTTATGCAGAGGCCGAGGCCGCCTCGGCCTCTGAGCTATTCAGAGTAGTGAGAGCTTTTTTGGAGGNNAGCTTTGCAAAAGCTGATTCTTCTGACNNAACAGTCTCGNNNNNNTGGAGCACATGNNTCANNGTACGACCGANCACGNNNNNCATGATCACNGNNNTCAGTGGNGGNNTNGCCTNCANNNN

>L5Y6305-2-GLO-F_B02.ab1NNNNNNAAATCGTGGACTATGTGGCCAGCCAGGTTACAACCGCCAAGAAGCTGCGCGGTGGTGTTGTGTTCGTGGACGAGGTGCCTAAAGGACTGACCGGCAAGTTGGACGCCCGCAAGATCCGCGAGATTCTCATTAAGGCCAAGAAGGGCGGCAAGATCGCCGTGTAATTCTAGTTGTTTAAACGAGCTC***TAATACTGCCTGGTAATGATGAC***AGCT***TAATACTGCCTGGTAATGATGAC***CTCGAGTCTAGAGTCGACCTGCAGGCATGCAAGCTGATCCGGCTGCTAACAAAGCCCGAAAGGAAGCTGAGTTGGCTGCTGCCACCGCTGAGCAATAACTAGCATAACCCCTTGGGGCGGCCGCTTCGAGCAGACATGATAAGATACATTGATGAGTTTGGACAAACCACAACTAGAATGCAGTGAAAAAAATGCTTTATTTGTGAAATTTGTGATGCTATTGCTTTATTTGTAACCATTATAAGCTGCAATAAACAAGTTAACAACAACAATTGCATTCATTTTATGTTTCAGGTTCAGGGGGAGATGTGGGAGGTTTTTTTAAGCAAGTAAAACCTCTACAAATGTGGTAAAATCGAATTTTAACAAAATATTAACGCTTACAATTTCCTGATGCGGTATTTTCTCCTTACGCATCTGTGCGGTATTTCACACCGCATACGCGGATCTGCGCAGCACCATGGCCTGAAATAACCTCTGAAAGAGGAACTTGGTTAGGTACCTTCTGAGGCGGAAAGAACCAGCTGTGGAATGTGTGTCAGTTAGGGTGTGGAAAGTCCCCAGGCTCCCCAGCAGGCAGAAGTATGCAAAGCATGCATCTCAATTAGTCAGCAACCAGGTGTGGAAAGTCCCCAGGCTCCCCAGCAGGCAGAAGTATGCAAAGCATGCATCTCAATTAGTCAGCAACCATAGTCCCGCCCCTAACTCCGCCCATCCCGCCCCTAACTCCGCCCAGTTCCGCCCATTCTCCGCCCCATGGCTGACTAATTTTTTTTATTTATGCAGAGGCCGAGGCCGCCTCGGCCTCTGAGCTATTCCAGAAGTAGTGAGAGCTTTTTGGAGNCTAGCTTTTGCAAAAGCTGATCTCTGACACAACAGTCTCGANNANNTGNNCCANCATGGCTTCAGNGTACGACCCGAGCACGNNNANNATGATCNCNGNNNTNANNNNNNNNTCGCNGCCAGNNAATGAACGGTGCCTNNNNNNN
