# Supplementary material for: MiR-200b in heme oxygenase-1-modified bone marrow mesenchymal stem cell-derived exosomes alleviates inflammatory injury of intestinal epithelial cells by targeting high mobility group box 3
Source: Cell Death Dis. 2020 Jun 25;11(6):480. doi: 10.1038/s41419-020-2685-8 (PMC7316799; doi:10.1038/s41419-020-2685-8)
Supplement: Supplementary file 2 — Supplemental figure legends [file 41419_2020_2685_MOESM2_ESM.docx]

**Supplemental figure legends**

**Supplemental figure 1. Related to Fig. 1. A** The cell morphology of the 3rd generation BMMSCs as observed under light microscopy. **B-C** BMMSCs could be induced to differentiate into osteoblasts (**B**) and adipoblasts (**C**). **D** CD29, CD34, CD45, CD90, RT1-A, and RT1-B were labeled as surface molecular markers of BMMSCs. **E** The expression of GFP was observed under a fluorescence microscope at 48 h after transfection of empty *Gfp*-adenovirus and *Ho-1*-overexpression adenovirus. BMMSCs; Bone marrow mesenchymal stem cells; CD29, integrin subunit beta 1; CD34, CD34 molecule; CD45, protein tyrosine phosphatase receptor type C; CD90, Thy-1 cell surface antigen; GFP, green fluorescent protein; HO-1, heme oxygenase-1; RT1-A, soluble MHC class I protein A; RT1-B, soluble MHC class I protein B.

**Supplemental figure 2. Related to Fig. 1F.** The GO functional enrichment path diagram (**A**) and GO functional enrichment chord diagram (**B**) based on DEGs between HO-1/BMMSCs and GFP/BMMSCs. BMMSCs; Bone marrow mesenchymal stem cells; DEGs: differentially expressed genes; HO-1, heme oxygenase-1; GFP, green fluorescent protein; GO: gene ontology.

**Supplemental figure 3. GO analysis and KEGG analysis of DAPs from IECs between the BM-exo-treated group and the TNF-α-exo-treated group (Relative to Fig. 4D).** BMMSCs, Bone marrow mesenchymal stem cells; BM-exo, BMMSCs co-culture system exosomes; DAPs, differentially abundant proteins; GO, gene ontology; IECs; intestinal epithelial cells; KEGG, Kyoto Encyclopedia of gene and genomes; TNF-α, tumor necrosis factor alpha; TNF-α-exo; TNF-α-treated IEC-6 cell system exosomes.

**Supplemental figure 4. GO analysis and KEGG analysis of DAPs from IECs between the HBM-exo-treated group and the BM-exo-treated group (Relative to Fig. 4D).** BMMSCs, Bone marrow mesenchymal stem cells; BM-exo, BMMSCs co-culture system exosomes; DAPs, differentially abundant proteins; GO, gene ontology; HO-1, heme oxygenase-1; HBM-exo, HO-1/BMMSCs co-culture system exosomes; IECs; intestinal epithelial cells; KEGG, Kyoto Encyclopedia of gene and genomes.

**Supplemental figure 5. GO analysis and KEGG analysis of DAPs from IECs between the HBM-exo-treated group and the TNF-α-exo-treated group (Relative to Fig. 4D).** BMMSCs, Bone marrow mesenchymal stem cells; BM-exo, BMMSCs co-culture system exosomes; DAPs, differentially abundant proteins; GO, gene ontology; HO-1, heme oxygenase-1; HBM-exo, HO-1/BMMSCs co-culture system exosomes; IECs; intestinal epithelial cells; KEGG, Kyoto Encyclopedia of gene and genomes; TNF-α, tumor necrosis factor alpha; TNF-α-exo; TNF-α-treated IEC-6 cell system exosomes.

**Supplemental figure 6. Related to Fig. 6E. A** The map of vector construction. **B** The expression of the green fluorescent protein (GFP) was observed under a fluorescence microscope. HMGB3, high mobility group box 3; MUT, mutant-type; WT, wild-type.

**Supplemental figure 7. Related to Fig. 7A. A** Immune rejection score of intestinal histopathology in rats in each group (Instant and 14 d post-surgery, n = 6, median). **B-C** The intestinal histopathological changes of small bowel transplantation (SBTx) rats in the Sham group and each group, as observed by hematoxylin and eosin staining.

**Supplemental figure 8. Quantitative analysis of proteins from western blotting (part 1). A** Related to Fig. 1B (fold change relative to BMMSCs). **B** Related to Fig. 2G (fold change relative to Mock group). **C** Related to Fig. 3J (fold change relative to Mock group). **D** Related to Fig. 4E (fold change relative to Mock group).

**Supplemental figure 9. Quantitative analysis of proteins from western blotting (part 2). A** Related to Fig. 5G (fold change relative to the Mock group). **B** Related to Fig. 6G (fold change relative to the Mock group). **C** Related to Fig. 6I (fold change relative to the Mock group). **D** Related to Fig. 7G (fold change relative to the Sham group).
