# Supplementary figures and images for: MiR-200b in heme oxygenase-1-modified bone marrow mesenchymal stem cell-derived exosomes alleviates inflammatory injury of intestinal epithelial cells by targeting high mobility group box 3
Source: Cell Death Dis. 2020 Jun 25;11(6):480. doi: 10.1038/s41419-020-2685-8 (PMC7316799; doi:10.1038/s41419-020-2685-8)

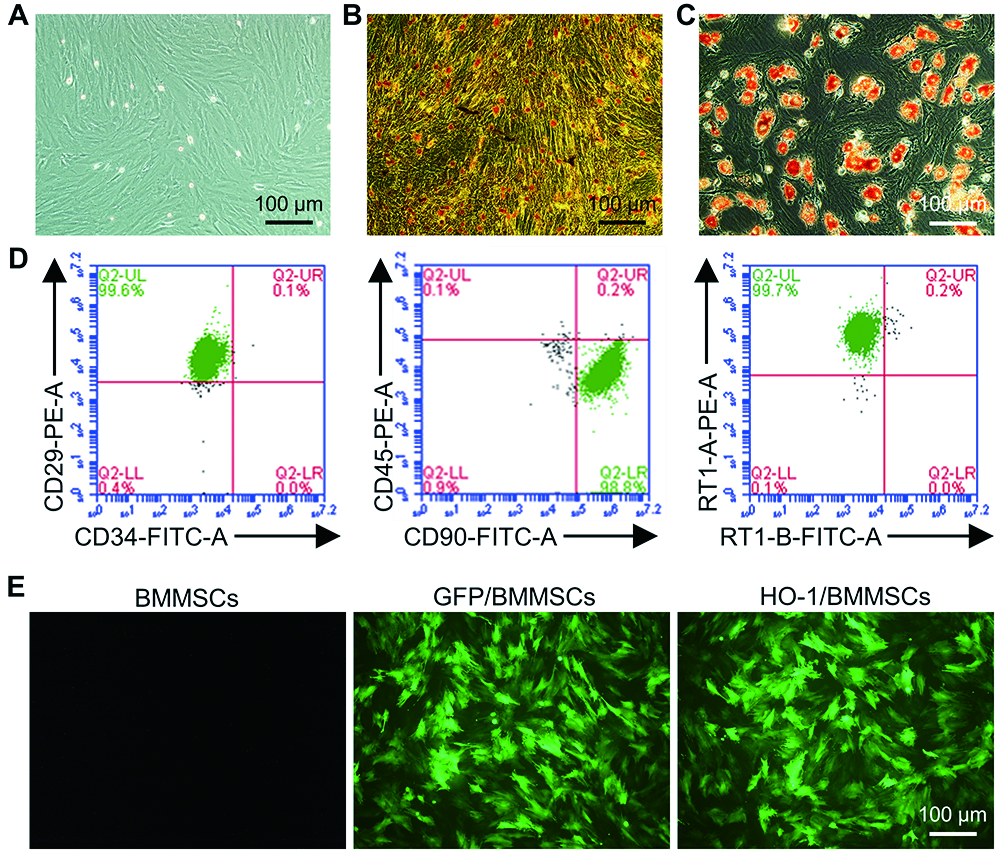

Supplement: Supplementary file 3 — Supplemental figure 1 [file 41419_2020_2685_MOESM3_ESM.tif]

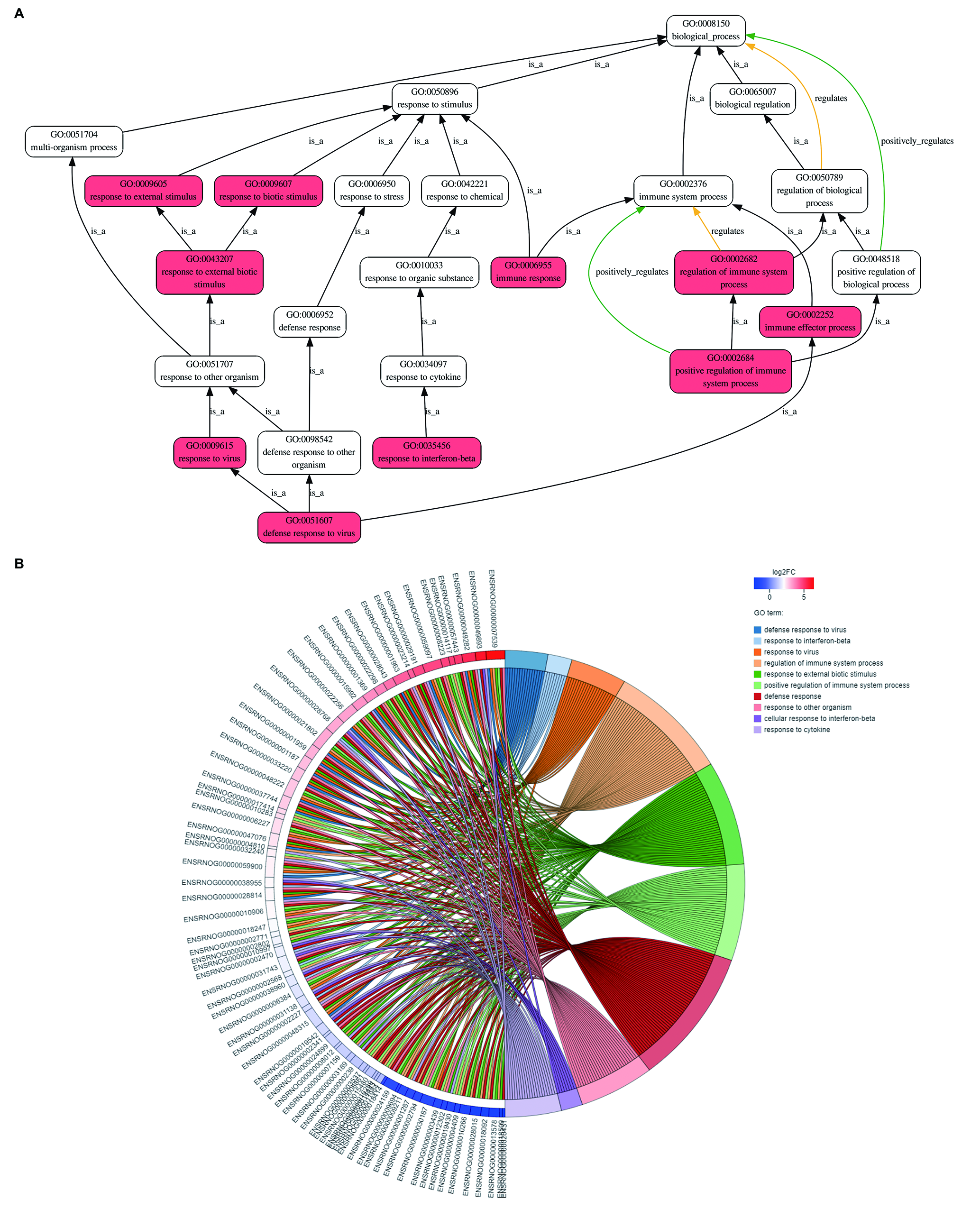

Supplement: Supplementary file 4 — Supplemental figure 2 [file 41419_2020_2685_MOESM4_ESM.tif]

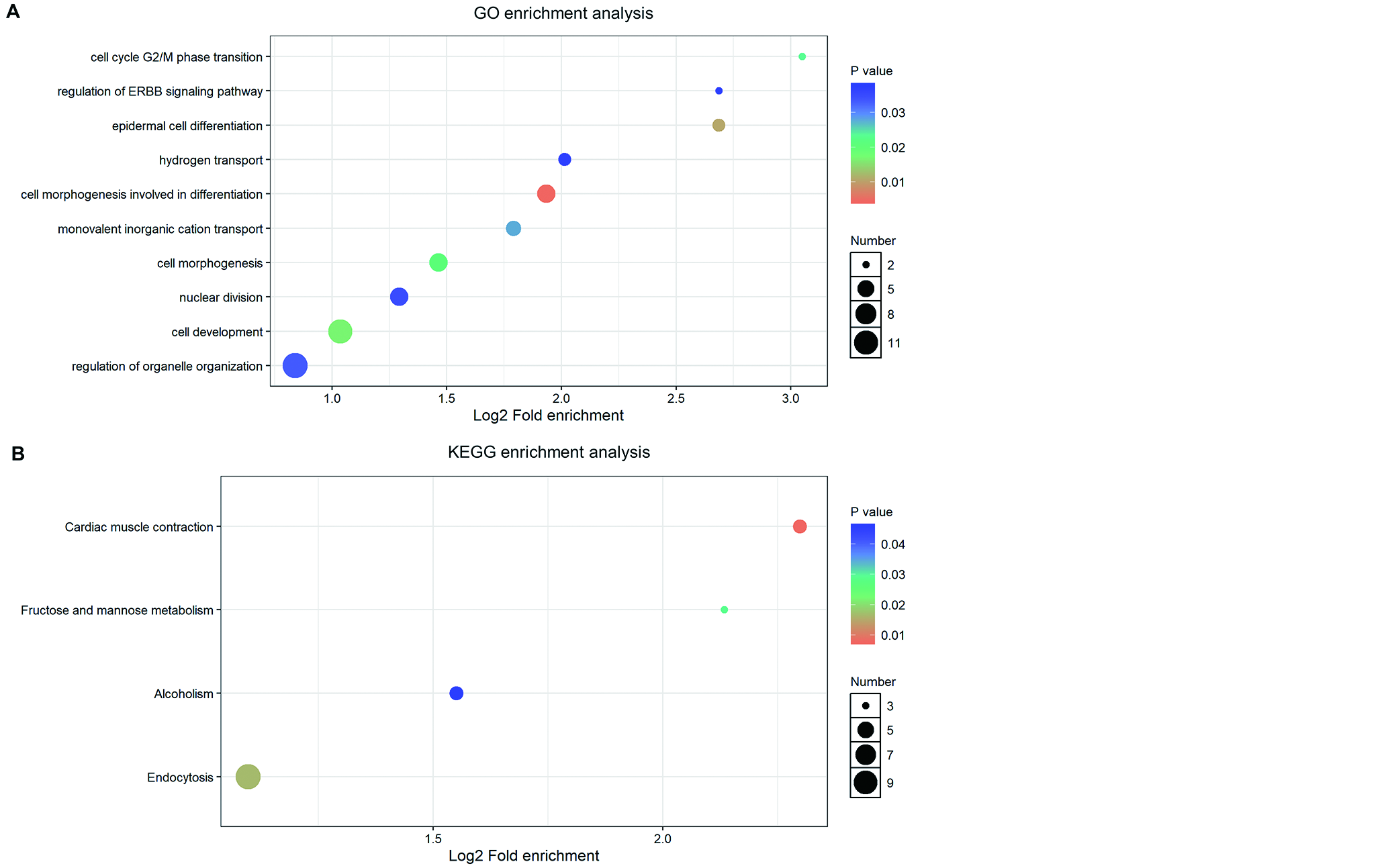

Supplement: Supplementary file 5 — Supplemental figure 3 [file 41419_2020_2685_MOESM5_ESM.tif]

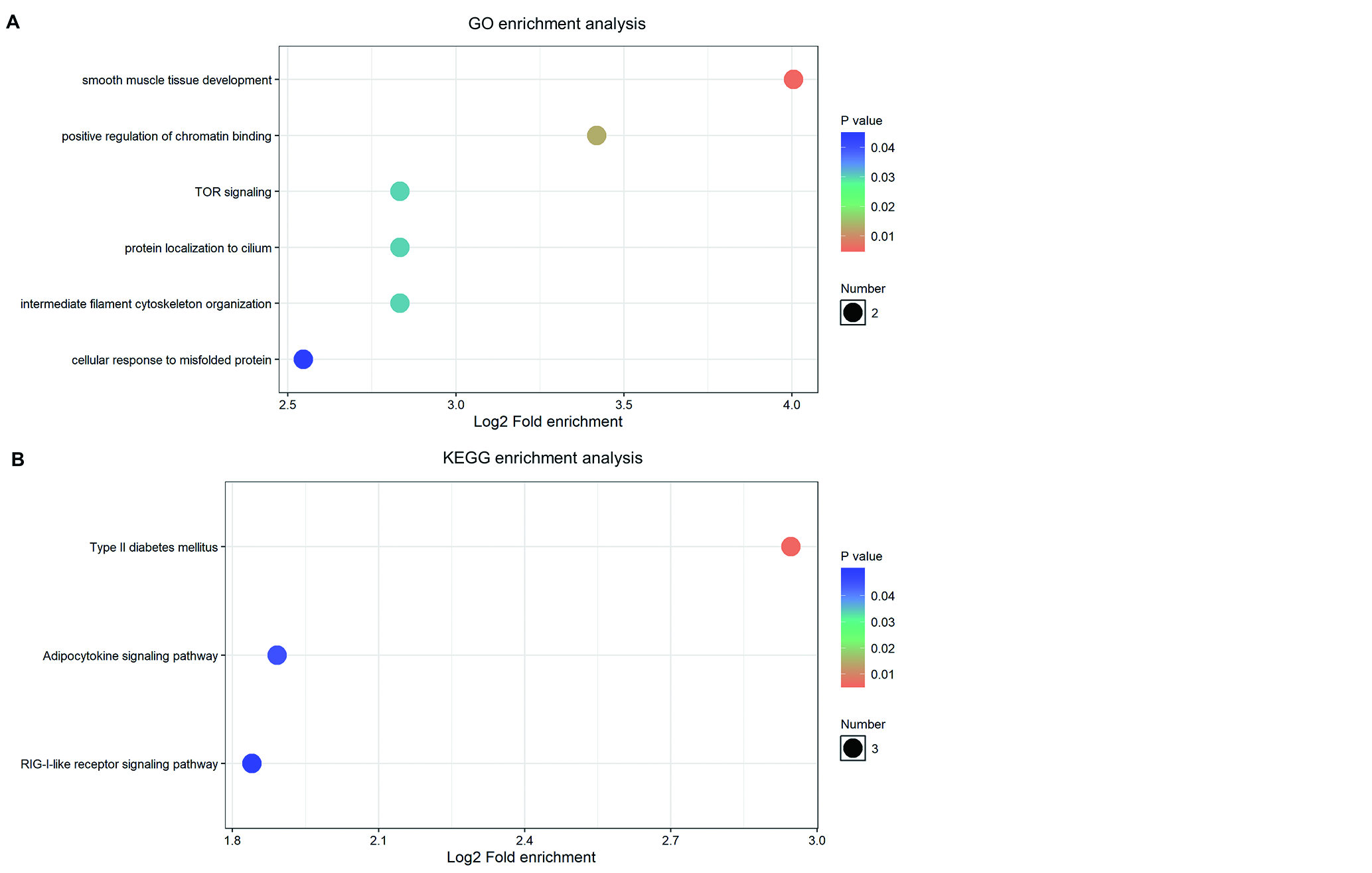

Supplement: Supplementary file 6 — Supplemental figure 4 [file 41419_2020_2685_MOESM6_ESM.tif]

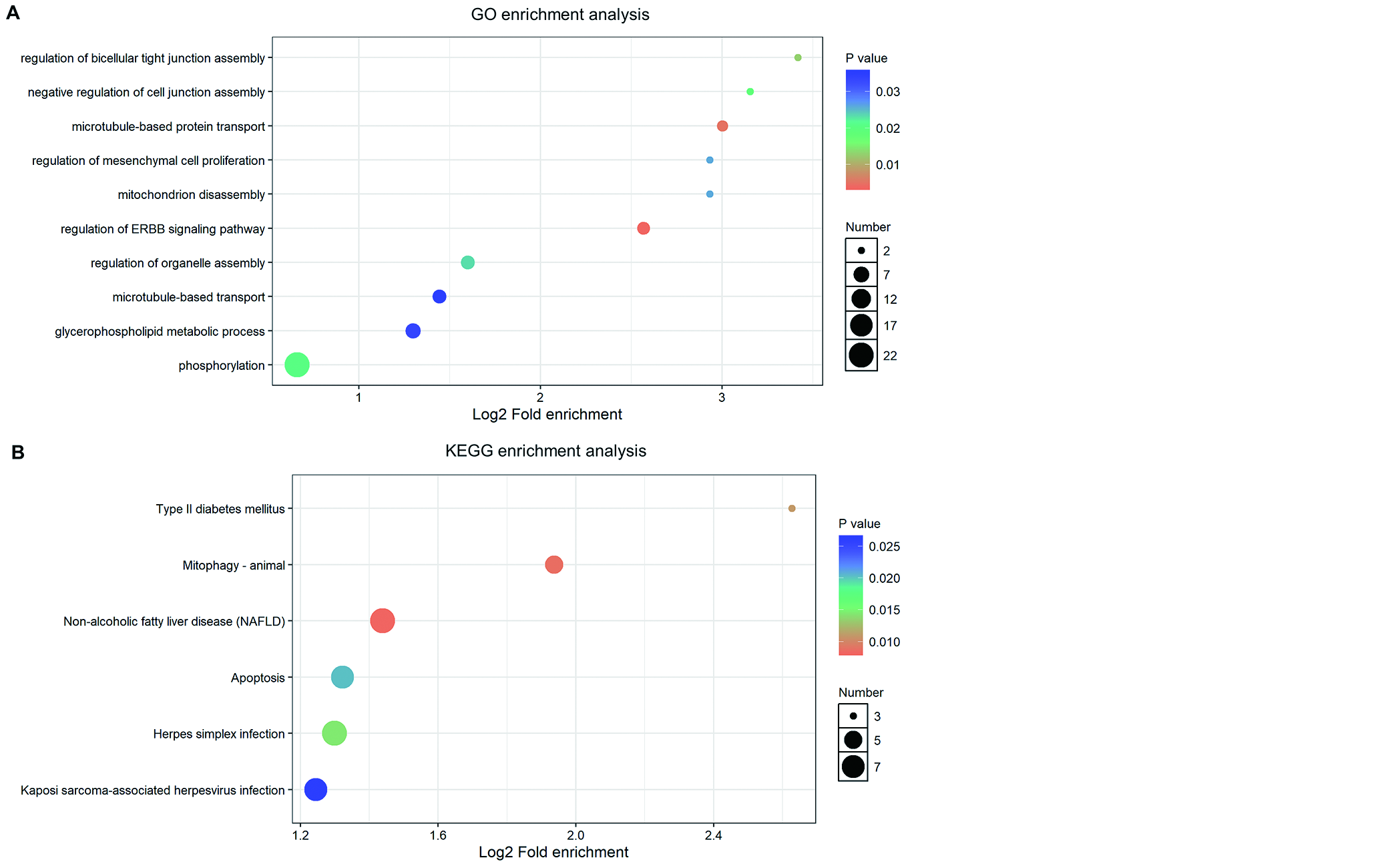

Supplement: Supplementary file 7 — Supplemental figure 5 [file 41419_2020_2685_MOESM7_ESM.tif]

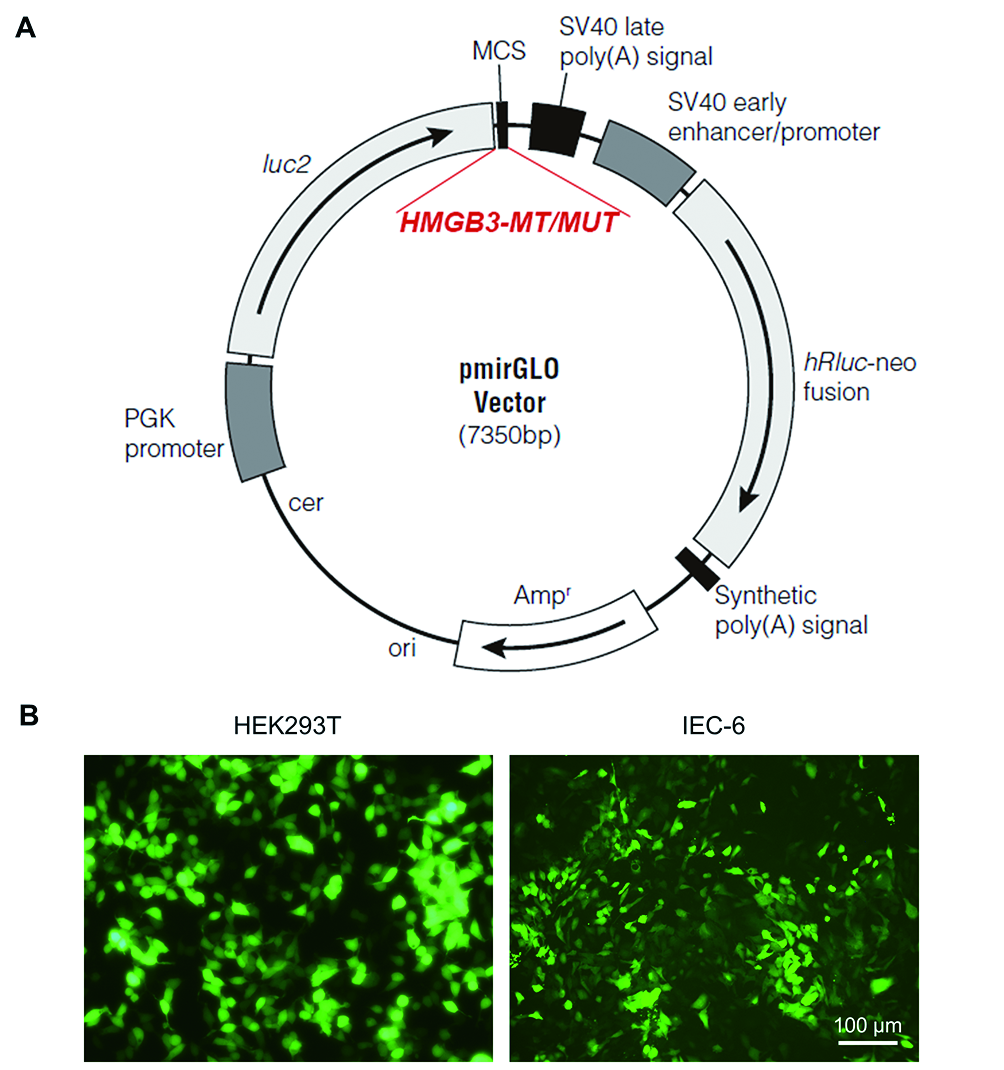

Supplement: Supplementary file 8 — Supplemental figure 6 [file 41419_2020_2685_MOESM8_ESM.tif]

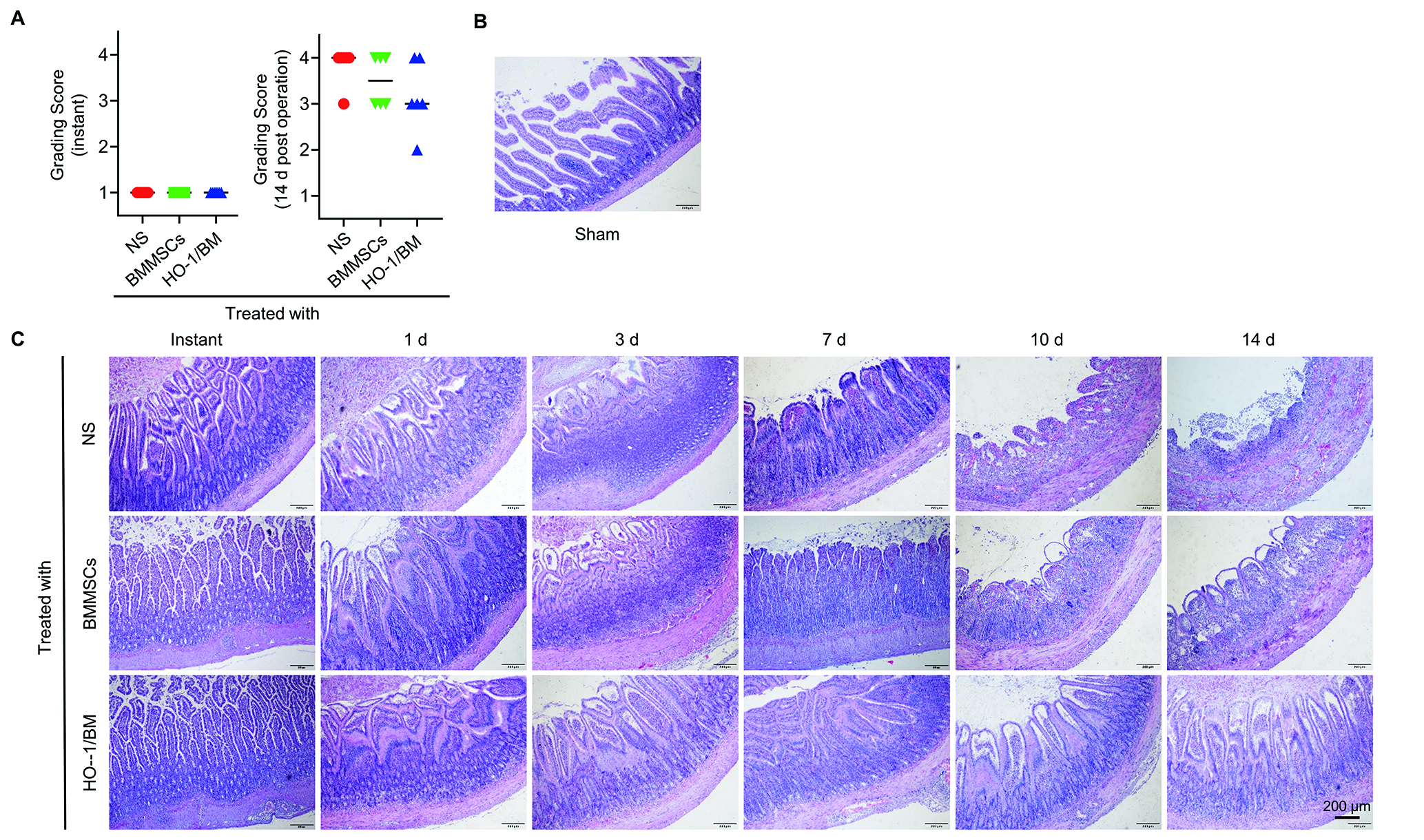

Supplement: Supplementary file 9 — Supplemental figure 7 [file 41419_2020_2685_MOESM9_ESM.tif]

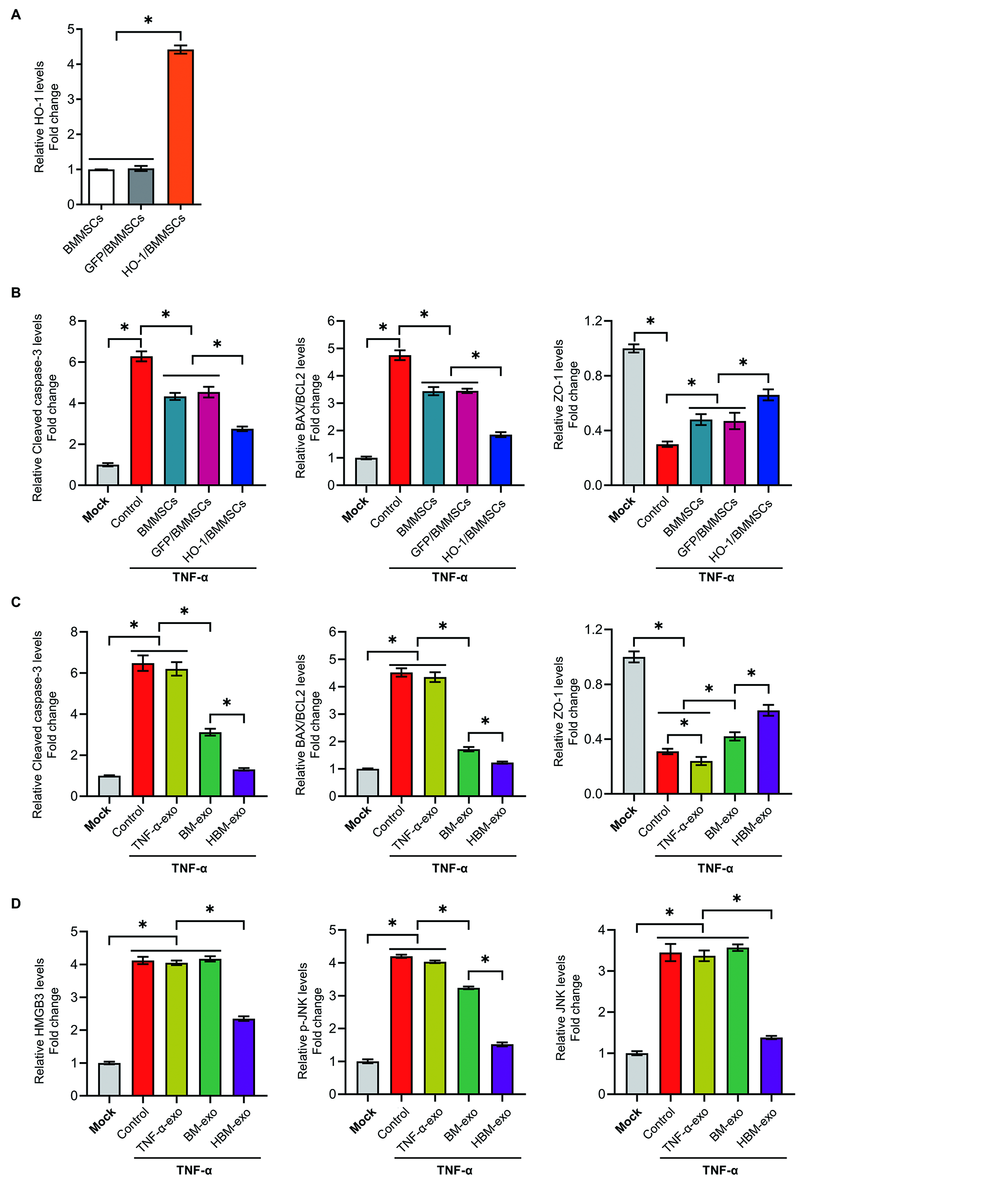

Supplement: Supplementary file 10 — Supplemental figure 8 [file 41419_2020_2685_MOESM10_ESM.tif]

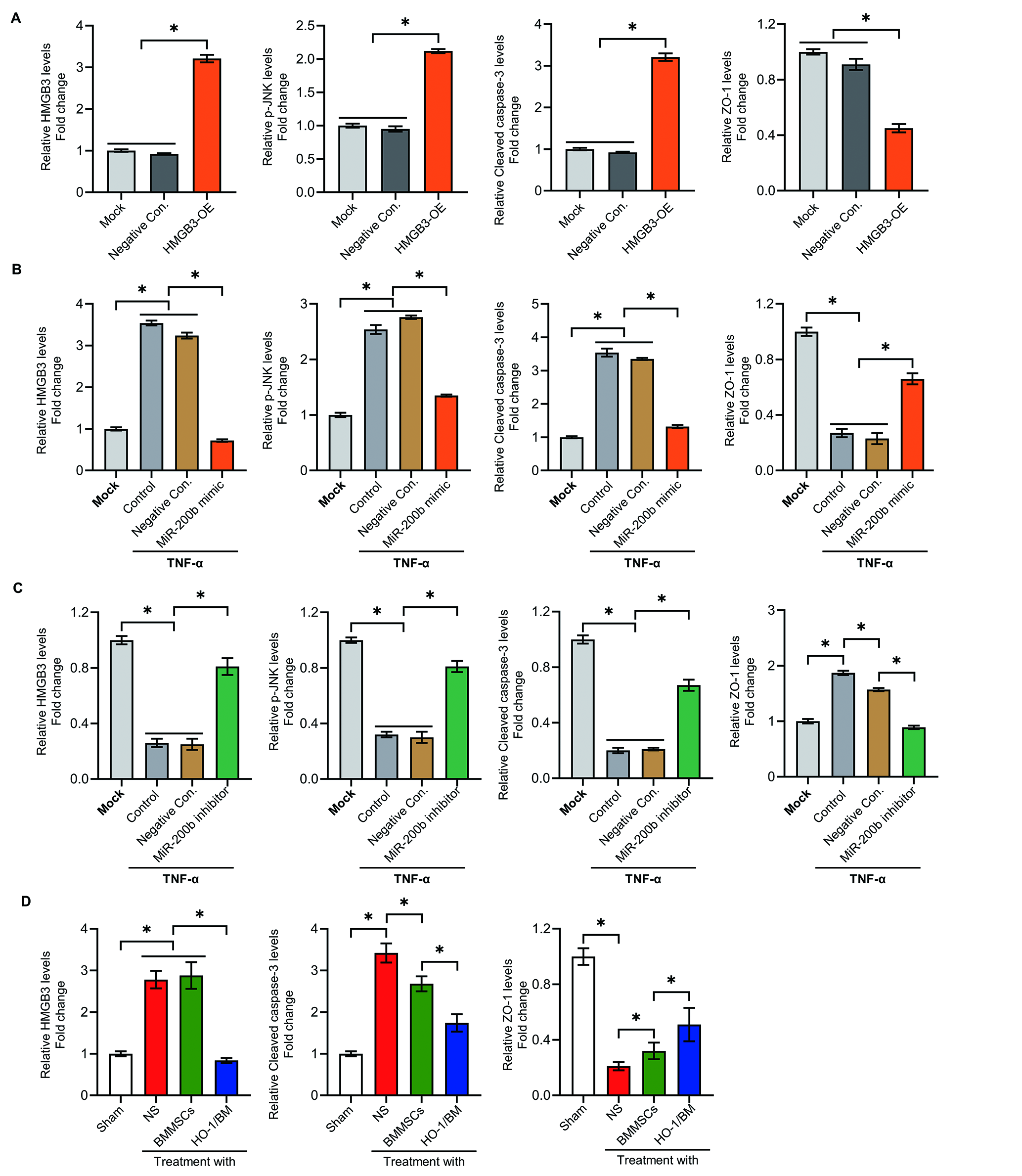

Supplement: Supplementary file 11 — Supplemental figure 9 [file 41419_2020_2685_MOESM11_ESM.tif]
